# Creating performance intelligence for primary health care strengthening in Europe

**DOI:** 10.1186/s12913-019-4853-z

**Published:** 2019-12-27

**Authors:** Erica Barbazza, Dionne Kringos, Ioana Kruse, Niek S. Klazinga, Juan E. Tello

**Affiliations:** 1 Amsterdam UMC, Department of Public Health, University of Amsterdam, Amsterdam Public Health research institute, Meibergdreef 9, 1105 AZ Amsterdam, the Netherlands; 2 WHO European Centre for Primary Health Care, Health Services Delivery Programme, Division of Health Systems and Public Health, Tole Bi 88, Almaty, Kazakhstan 050000; 3 Integrated Prevention and Control of NCDs Programme, Division of NCDs and Promoting Health through the Life-Course, WHO Regional Office for Europe, Marmorvej 51, 2100 Copenhagen, Denmark

**Keywords:** Primary health care, Performance assessment, Health systems, Measurement, Primary care, Europe

## Abstract

**Background:**

Primary health care and its strengthening through performance measurement is essential for sustainably working towards universal health coverage. Existing performance frameworks and indicators to measure primary health care capture system functions like governance, financing and resourcing but to a lesser extent the function of services delivery and its heterogeneous nature. Moreover, most frameworks have weak links with routine information systems and national health priorities, especially in the context of high- and middle-income countries. This paper presents the development of a tool that responds to this context with the aim to create primary health care performance intelligence for the 53 countries of the WHO European Region.

**Methods:**

The work builds-off of an existing systematic review on primary care and draws on priorities of current European health policies and available (inter)national information systems. Its development included: (i) reviewing and classifying features of primary care; (ii) constructing a set of tracer conditions; and (iii) mapping existing indicators in the framework resulting from (i). The analysis was validated through a series of reviews: in-person meetings with country-nominated focal points and primary care experts; at-distance expert reviews; and, preliminary testing with country informants.

**Results:**

The resulting framework applies a *performance* continuum in the classical approach of structures-processes-outcomes spanning 6 domains – primary care structures, model of primary care, care contact, primary care outputs, health system outcomes, and health outcomes – that are further classified by 26 subdomains and 63 features of primary care. A *care* continuum was developed using a set of 12 tracer conditions. A total of 139 indicators were mapped to the classification, each with an identified data source to safeguard measurability. Individual indicator passports and a glossary of terms were developed to support the standardization of the findings.

**Conclusion:**

The resulting framework and suite of indicators, coined the Primary Health Care Impact, Performance and Capacity Tool (PHC-IMPACT), has the potential to be applied in Europe, closing the gap on existing data collection, analysis and use of performance intelligence for decision-making towards primary health care strengthening.

## Background

Four decades following the adoption of the Declaration of Alma-Ata, the evidence base for primary health care (PHC) as the most inclusive, effective and efficient approach to make progress towards universal health coverage (UHC) and enhance population health [1–4] has solidified a PHC approach as the ambition of countries worldwide [5]. In spite of progress made to strengthen PHC, in 2019 the global health community is confronted with the work still to be done. From a European perspective this includes widening inequities and gender differences for noncommunicable disease (NCD) outcomes, the substantial burden of mental illness, rapid population ageing and the global threat of antimicrobial resistance [6–8]. The region also faces persisting quality deficiencies, increasing vulnerable groups, and impoverishing levels of out-of-pocket payments [7, 9, 10].

With the 2030 Sustainable Development Goals (SDGs) on the horizon there is renewed impetus and political commitment for PHC strengthening as an accelerator towards UHC [5]. For this, measuring the performance of primary care services has a fundamental role.

A PHC approach from a services delivery perspective can be characterized as primary care: the continuum of first contact promotive, preventive, diagnostic, curative, rehabilitative and palliative care services delivered across the life-course [5]. How countries select, design, organize, manage and improve their primary care services is heterogeneous. As a result, there is considerable diversity in the scope of primary care services, types and profiles of health professionals like nurses, general practitioners, social workers, public health professionals, narrow specialists, paediatricians or occupational therapists, and settings of care like single offices of general practitioners, group practices, multi-profile teams or polyclinics.

Without primary care performance measurement sensitive to these differences, countries often lack the necessary information to monitor and evaluate their options for improvement. Despite the numerous initiatives to strengthen primary care measurement [11–15], the lack of comparable data on primary care in Europe continues to limit performance intelligence for decision-making. Of the factors contributing to this we highlight three.

First, there is no single approach to provide basic, up-to-date information on the organization and delivery of primary care. This is in contrast to internationally comparable information on financial resources (e.g. System of Health Accounts) or professional classifications (e.g. International Labour Standards) that define a more standardized approach. Importantly, even these standards face constraints to capture primary care, such as for making international comparisons on its costs and workforce. The challenge of comparability is especially relevant in the context of the WHO European Region as member countries range from western, eastern and southern Europe, the Baltic countries, central Asia and the Caucasus.

Second, frameworks defined for global use are strained to measure variations for health outcomes that matter most to European countries. The World Health Organization’s (WHO) global UHC service coverage index illustrates this point. According to 2017 reporting, nearly 40% of European countries had an average coverage score of 77 or more on the index; the highest globally [16]. However, the inclusion of tracer conditions and services like malaria and sanitation limits the sensitivity of the index to high- and middle-income countries. Global frameworks are also strained to capture European policy priorities, like the importance the region’s member countries have weighted to people-centred services [17] and measurement of patient experiences [18, 19].

Third, most primary care frameworks and performance assessments have weak links with routine information systems and national health priorities [20]. This is despite the wide uptake of electronic information systems and health records in primary care across Europe [21, 22]. It means primary care monitoring efforts have yet to fully leverage and integrate existing data infrastructure to best support evidence-informed decision-making [23, 24].

This paper describes the development of a new tool for monitoring PHC performance across the 53 member countries of the WHO European Region. Our research was guided by the aim to create robust performance intelligence in Europe that captures the ability of primary care to respond to population health needs. Specifically, this work responds to the policy commitment of the WHO European Region member countries enacted in 2016, calling for intensified regional monitoring on health services delivery [25], and is supplementary to global monitoring efforts, like monitoring UHC [16] and foreseen monitoring framework for the implementation of the Astana Declaration [5].

## Methods

The following details the processes we undertook between mid-2016 and 2018 in a three-staged approach: first, reviewing and classifying features of primary care, second, constructing a set of tracer conditions and third, mapping existing indicators in the framework devised in the first stage.

### Targeted literature review of features of primary care

As a starting point, we reviewed the literature for characteristics of primary care in existing frameworks, tools and surveys. We took as a basis a study by Kringos et al. (2010) being the most comprehensive review on the core dimensions of primary care to-date [26]. To update the review, we extracted priority areas and strategies of a contemporary European policy: the WHO European Framework for Action on Integrated Health Services Delivery [25]. The policy was developed and endorsed by countries and aligns with current European policies [18, 27] making it highly attuned to policy priorities in the region.

A literature review search strategy was developed to target recently published scientific and grey literature on frameworks and tools for health services delivery in general, and primary care in particular [28]. We conducted initial searches between October 2016 and May 2017 using PubMed to identify scientific literature published since 2010. We brought the existing systematic review up-to-date by searching new key terms including: avoidable hospitalization; chronic disease management; community-based care; drugs and medical devices; financial incentives; information systems; integrated care; job satisfaction; patient-centredness; patients with complex needs; population health management; responsible use of medicines; role of nurses; shared care plans; task-shifting; technology assisted care; unmet need; waste and appropriateness of care; and workload.

We hand-searched websites and databases of key international actors active in monitoring PHC, namely WHO using WHOLIS, the World Bank, Organisation for Economic Co-operation and Development (OECD) and European Commission and collated sources already known to the authors. Reference lists of relevant work identified were also reviewed and titles were searched in a snowballing approach. With the exception of global frameworks and a recent PHC initiative for low- and middle-income countries [13], work from the European context was prioritized.

Two authors (EB,DK) completed the initial document review. For each framework identified, the features of primary care, their respective classification and key definitions were extracted and logged in an Excel spreadsheet. The authors jointly carried out an analysis of the review findings to identify crosscutting themes.

### Review of tracer conditions

To tailor the framework to the European context, we used the method of tracer conditions [29], like applied to monitoring UHC globally [16, 30]. Tracer conditions have been used in health services research on the premise that a carefully selected set of health problems makes it possible to profile the strengths and weakness of services delivery and health systems [31]. On this basis, we sought to construct a set of tracer conditions to inform the selection of indicators that–when analysed together– could serve to gauge the ability of primary care to respond to a range of health needs individually and concurrently as multimorbidities, while also measuring across population groups and life stages.

The selection of tracer conditions prioritized the following: relevance to the burden of disease in Europe; responsiveness to the strength of primary care; and representativeness of primary care’s functions. The final selection also gave consideration to the measurability of conditions and the parsimony of the set, weighing together the balanced representation of different target populations and life stages, gender importance and types of services. The overall manageability of the set was also prioritized for the selection of a core group of tracers that could serve the purpose of scoping the tool to high-priority health improvement areas.

Current global and European health policies were reviewed by two authors (EB,IK) as a proxy for priority health improvement areas. To prioritize conditions amendable to primary care, European lists of ambulatory care sensitive conditions were consulted [32], together with priority conditions included in an earlier study on the strength of primary care in Europe [12]. To achieve a comprehensive and holistic view to primary care, the type of condition (e.g. acute, chronic), relation to the life-course, gender importance and function of primary care (e.g. prevention, detection, treatment, management) were also considered. For each possible tracer condition, findings for the criteria considered were recorded.

### Identifying existing indicators

To identify existing, internationally standardized indicators, we searched by features of the framework resulting from the first stage, in databases of international organizations (WHO, OECD, European Commission) and topic-specific databases of research consortiums e.g. cancer, medicines and tuberculosis. We reviewed existing global and European surveys related to health services delivery, patient-reported experience and outcome measures or conditions amendable to the strength of primary care. Standardized country reports by international organizations were also reviewed through searches on their respective websites. Indicators from the initial literature review and health strategies used to select tracer conditions were also extracted.

To select indicators for the framework, we prioritized the following:

- measurability through an existing or feasible data source;
- available internationally standardized indicators and survey questions;
- relevance to the European context;
- balanced coverage across the framework and its classification; and,
- balanced representation of perspectives e.g. patients, practitioners, policy-makers.

We prioritized the first criterion on data availability in our selection to align to information systems and make use of the vast amount of available data, also minimizing the burden of new data collection. We undertook a detailed process of identifying sources by drawing on an existing study on health information systems [22]. We further expanded upon this to scan the availability of health services delivery data across Europe. The findings from this scan have been published elsewhere [21] and were used to ensure existing data sources are drawn from. Alternative sources where applicable, such as national documents (e.g. health policies, directives or prikaz, guidelines) or key informants, were also considered as part of asserting the measurability of indicators to merit their selection.

Indicators were mapped to the classification resulting from stage one. For the purposes of this mapping, indicator passports were developed to clearly define the scope and measurement of each individual indicator. The indicator passports draw from the literature reviewed and existing international standards where available to detail the following: alignment to the framework (domain, subdomain, feature), indicator/question title, indicator/question definition, numerator/denominator or answer choices, unit of measurement, rationale, relevant definitions, disaggregation, known limitations and possible data sources. A glossary of terms accompanies the indicator passports with emphasis put to capturing different terminology used in the European countries. The terms and definitions draw from existing international classifications including the International Classification for Health Accounts, International Standard Classification of Occupations and International Standard Classification of Education.

### Validation of the framework, tracers and indicators

Country-nominated focal points representing ministries of health, health insurance funds, centres on health services or similar from 30 countries assessed face and content validity of the framework and selection of tracer conditions at a meeting in June 2017 [33]. The set of tracer conditions was also presented and validated with country representatives. They were asked to consider the relevance and comprehensiveness of the framework and tracer conditions in relation to their systems. In the same period, the framework was presented to members of WHO’s European Primary Health Care Advisory Group – a group of appointed experts to support the continued advancement of PHC [34]. All comments and discussion points were recorded and adjustments to features were made for a revised version of the framework that was then applied for the mapping and selection of indicators.

To review the indicators identified, we engaged more than 40 experts between November 2017 and June 2018. Reviewers spanned three profiles: (i) staff of WHO; (ii) experts in relevant fields from academia, think tanks and international organizations; and (iii) representatives of professional and patient associations, as well as practising clinicians.

Attention was put to ensure that reviewers were representative of countries across the region and included a range of language skills – with approximately one quarter (23%) being native Russian-speakers – and gender balance (49% females, 51% males). Nearly half of reviewers (42%) were trained medical doctors. Reviewers also included information specialists on European and central Asian countries.

Each reviewer received a written request for their feedback on a subset of indicators. Reviewers were provided the indicator passport, background on the framework and selection criteria. They were asked to score the indicator’s overall quality, provide comments or amendments and/or suggest an alternative indicator or source. One author (IK) consolidated the feedback from all reviewers. Indicators that were rated of poor quality, too vague or not meaningful for analysis were removed. For others, comments were used to revise the indicator passports. Comments included important feedback for updating indicators to current international standards as well as making explicit the limitations of indicators where known and for identifying alternative sources of data if available.

Further to technical reviews, we also conducted a preliminary test of the framework and indicators as an initial validation of its use in practice. The indicators were translated to Russian and applied in Kazakhstan on the basis of testing their applicability in the context of a Russian-speaking country and the characteristics and cultural nuances of health systems in central Asia and Caucasus countries. This process extended from December 2017 to June 2018 and included a series of workshops with national centres responsible for data collection. Further revisions were incorporated into the indicator passports and the glossary of terms.

## Findings

**Fig. 1:**
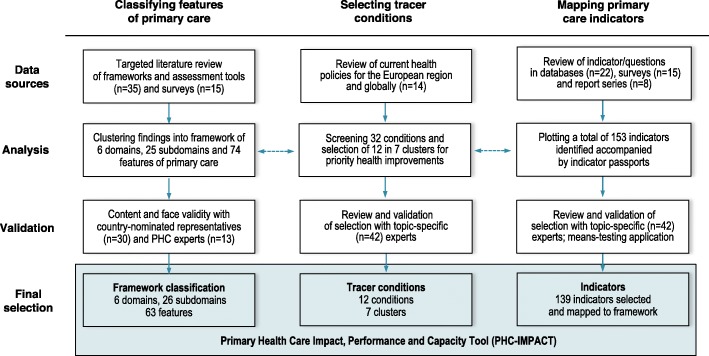
Summary of findings

The findings across the three stages of our research process are summarized in Fig. 1 and described to follow. Supplementary files of the literature reviewed, selection of tracers, individual indicator passports and glossary of key terms is available electronically [35].

### Classifying features of primary care

We found a total of 50 different frameworks, assessment tools and survey instruments, recorded in Additional file 1 [35]. From the literature reviewed, we identified approximately 50 domains and 100 features of primary care. We further analysed these findings to identify crosscutting themes and to cluster related features. We noted several areas of clear consensus in the literature. For example, the contribution of primary care to first contact access, comprehensive services and continuity and coordination of care is recognized across frameworks [12, 14, 33, 36].

We developed a hierarchy for the classification of findings, adopting the classical framework of Donabedian’s structure-process-outcome logic model [37]. These components were classified and sequenced as the capacity, performance, and impact of primary care. We defined a taxonomy to describe these components from broad to specific as domains, subdomains, and features [35].

Under *primary care capacity*, we put attention to disaggregate the unique yet often over-looked resource needs of primary care, including the primary care workforce, medicines, diagnostics, facility infrastructure, and information system. We were also attentive to differentiate the system’s capacity, as the enabling *system structures* underpinning primary care, from the function of services delivery. We classified this as the *model of care*: the result of deliberate decisions taken that determine the contents, design, organization, management and quality improvement elements of services delivery.

This classification is a point of departure from existing frameworks. It captures the less prevalent ‘software’ of primary care’s capacity [13], such as managerial autonomy for determining staffing, budgeting and strategic planning in primary care facilities, prescribing authority of general practitioners to initiate or refill prescriptions and the existence and scale of quality improvement mechanisms, like practice audits, patient complaint systems or peer review teams. The taxonomy around the selection of services spans from identifying needs, to the selection of preventive care, diagnostic procedures, treatment and disease management services as well as services for supporting self-management. The model of care also bridges between the system on one side, and the provision of services and perspective of patients, on the other.

This domain, at the intersection with the *performance of primary care*, is captured as *care contact*. It is novel in distinguishing the ‘structural’ from its implications on performance. For example, we find skill-mix and multidisciplinary teams an agreed upon feature of primary care [12, 14, 33, 38, 39]. However, it has typically been classified as a feature of coordination. We argue the setup and structure of teams captures how providers are organized and their resulting level of interaction and joint-work rather as a measure of coordination.

Importantly, while in the reviewed literature there was clear consensus on the policy importance of capturing the perspective of patients, to a lesser extent had this priority area been translated into monitoring frameworks. The domain of *care contact* collocates patient experience as a core feature of overall primary care performance and is found an important distinction from earlier classifications.

**Fig. 2:**
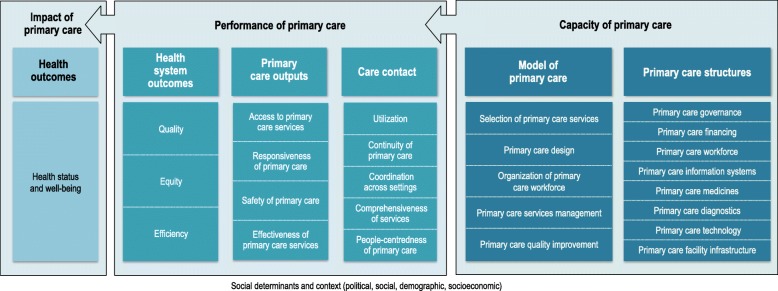
Monitoring framework

In line with existing system frameworks, *outputs* via services delivery (access, responsiveness, safety, effectiveness) and *health system outcomes* (quality, equity, efficiency) were classified from a services delivery perspective, linking to the final component of *impact* on health outcomes (health status and well-being). Importantly, in distinguishing between *outputs* from *outcomes*, we recognize that suboptimal outcomes can be attributed to features of the health system beyond the scope of primary care services and have labelled it as such. Figure 2 illustrates the resulting framework. Like other frameworks, we acknowledge that primary care performance lies within larger socio-political contexts, though these factors are outside the scope of health systems [12, 19, 36].

Adopting the approach of people-centred systems, the framework begins with health outcomes. In this way, health outcomes are the lens through which capacity and performance are monitored. By cascading the framework’s domains, features and subdomains as shown, the taxonomy applies system thinking principles to associate connections between capacity, performance and impact. Importantly, as the field of systems thinking has revealed, changes to systems can have unpredictable and multi-direction results [40].

### Selecting tracer conditions

Fourteen disease-specific strategies actively being implemented in Europe and globally were identified and reviewed (Additional file 2). We screened 32 conditions, identified first and foremost as priority health improvement areas in Europe and selected a set of 12 grouped in 7 clusters based on our selection criteria. These conditions span: reproductive, maternal, neonatal and child health; communicable diseases; cardiovascular diseases; diabetes; respiratory diseases; cancer; and mental health (Table 1).

**Table 1:** Selected tracer conditions

Cluster		Condition or services	Classification	Target population/life-stage^1^	Gender importance	Type of service^2^
1	Reproductive, maternal, neonatal and child health	post-natal care	service	infant; adolescents; adults	women and infants	T, M
2	Communicable	influenza	vaccine-preventable	children older adults	both	P
		tuberculosis	chronic	all	both	P, D, T, M
3	Cardiovascular diseases	hypertension	chronic	adults; older adults	both	P, D, T, M
		heart disease	chronic	adults; older adults	both	P, D, T, M
4	Diabetes	diabetes type II	chronic	adults; older adults	both	P, D, T, M
5	Respiratory	chronic obstructive pulmonary disease	chronic	adults; older adults	both	P, D, T, M
		asthma	chronic	childhood – onwards	both	P, D, T, M
6	Cancer	breast	chronic	adults	women	D, M
		cervical	vaccine-preventable	adolescents	women	P, D, M
		colorectal	chronic	older adults	men	D, M
7	Mental health	depression	chronic	adolescents – onwards	both	P, D, T, M

^1^Life-stage translated to age ranges: infant (0 to 1 year); children (1 to 10 years); adolescent (11 to 19 years); adults (20 to 59 years); older adults (60+ years)

^2^Type of service – *P* prevention; *D* detection; *T* treatment; *M* management

The selection process included a review within and across clustered to assess the conditions in combination in order to gauge the balance across the criteria applied and the manageability of the set. Guided by our primary aim to select a core set of tracers for profiling primary care, we prioritized one or two conditions per cluster, with one exception (cancer). In a last stage, we also considered the measurability of conditions in primary care [21], resulting in the exclusion of those specific to ageing, like dementia. Nonetheless, ensuring the representativeness of varied population groups and life stages was among the core selection criteria applied. For example, a range of primary care services for older adults are included such as, influenza vaccines (prevention), colorectal cancer (detection, management) and cardiovascular diseases (prevention, detection, treatment, management).

### Mapping primary care indicators

More than 20 international databases were reviewed to extract existing and reported on indicators. Fifteen global and European surveys were also reviewed on topics including medicines and e-health, patient experience, primary care doctors, and on tracer conditions like influenza and NCDs. An additional 8 standardized country reports were identified e.g. Health Systems in Transition series, OECD country reviews and WHO country assessments.

From this, we consolidated a total 243 indicators/questions. We plotted the indicators in our classification of subdomains/features. When mapped, we found the largest number of indicators for the structure domain (health workforce, financing) and health impact (morbidity, mortality), consistent with their wide use in health system performance frameworks [20]. The model of care domain was the least populated from this initial mapping and further targeted reviews were conducted to identify indicators for its features. In a final stage of development, we reassessed the initial classification with the indicators identified for refinements at the feature-level. Following this, three features remained without assigned indicators: acceptability, equity, and responsiveness.

We applied the tracer conditions to scope the selection and explicitly link indicators across the framework using theory of change principles as described [41]. In doing so, health outcomes are preceded by related performance and capacity indicators. For example, impact indicators on diabetes link to preceding diabetes-related performance indicators e.g. hospitalizations, managed insulin-levels, and capacity indicators e.g. prevention services for diabetes, existence of patient registries. Indicators that measured conditions outside the scope of the selected tracer conditions were excluded. A balance in the number of indicators for each tracer condition was also sought. We retained relevant disaggregations, such as public-private mix and rural-urban status for analysis purposes. The full set of indicator passports is available electronically in Additional file 3 and related glossary of terms in Additional file 4 [35].

### Final selection and new tool

We consolidated our findings in a tool we refer to as the Primary Health Care Impact, Performance and Capacity Tool (PHC-IMPACT). The tool includes a total of 139 indicators mapped to a detailed framework in a hierarchy of domains (6), subdomains (26), and features (63) as summarized in Table 2 and expanded in Table 3.

**Table 2:** Overview of final framework and suite of indicators

	Subtotals across domains						Totals
Domains	Health outcomes	Health system outcomes	Primary care outputs	Care contact	Model of primary care	Primary care structures	6
Subdomains	1	3	4	5	5	8	26
Features	2	4	6	11	21	19	63
Indicators	7	8	13	29	40	42	139

**Table 3:** Overview of framework for monitoring the impact, performance and capacity of PHC, indicators and data sources

Domain	Subdomain	Feature	Indicator title	Expected data sources
Primary care structures	Primary care governance (GOV)	GOV1. Primary care priorities	Primary care strategy	policy; key informant
		GOV2. Accountability arrangements	Primary care mandate	key informant
			Primary care resources	key informant
			Public health services mandate	report; key informant
		GOV3. Stakeholder participation and engagement	Roles of professional associations of generalist medical practitioners	key informant
			Roles of professional associations of nurses and midwives in primary care	key informant
			Roles of patient and/or consumer groups	report; survey; key informant
		GOV4. Quality assurance mechanisms	Quality assurance of health professionals	policy; key informant
			Quality assurance of facilities	policy; key informant
			Development of primary care clinical practice guidelines	policy; survey; key informant
			Patient rights and choice	policy; report; key informant
	Primary care financing (FIN)	FIN1. Primary care expenditure	Total primary health care expenditure as a share of total health expenditure	database
			Domestic primary health care expenditure	database
			Capital and recurrent expenditure arrangements	key informant
		FIN2. Payment methods in primary care	Provider payments	report; survey; key informant
			Employment status and remuneration of generalist medical practitioners	survey; report; key informant
			Pay-for-performance	survey; key informant
			Support for caregivers/family carers	report; key informant
		Benefit package	Services included in the health benefit package	report; survey; key informant
	Primary care workforce (WRK)	WRK1. Primary care workforce planning	Type of primary care health professionals	policy; key informant
			Scope of practice for primary care health professionals	policy; key informant
			Incentives for recruitment and retention in underserved areas	policy; key informant
			Retraining programme for specialist medical practitioners/narrow specialists	key informant; database
			Workforce registry with information on primary care professionals	survey; key informant
		WRK2. Financial status of general practitioners	Relative financial status of generalist medical practitioners	database
		WRK3. Primary care workforce availability	Age distribution of generalist medical practitioners	database
		WRK4. Academic status of primary care	General practice/family medicine undergraduate/bachelor education	key informant
			General practice/family medicine postgraduate education	key informant
			General practice/family medicine postgraduate clinical practice	key informant
			General practice/family medicine specialization among medical students	database
			Nurses working in primary care undergraduate/bachelor and postgraduate education	key informant
			Professional journal on general practice/family medicine	key informant
	Primary care information system (INF)	INF1. Data capture	Electronic health records system	survey; key informant
			Electronic health record system linked to clinical systems	survey; key informant
		INF2. Aggregation of data	Patient registries	survey; key informant
		INF3. Patient platforms	Use of mHealth in primary care	survey; key informant
	Primary care medicines (MED)	MED1. Availability of medicines	Reimbursement eligibility scheme for outpatient medicines	policy; survey; key informant
			Availability of essential medicines for primary care	survey; database; expert consensus
	Primary care diagnostics (DGN)	DGN1. Laboratory	Availability of laboratory tests in primary care	survey; database; expert consensus
		DGN2. Imaging	Availability of diagnostic imaging in primary care	survey; database; expert consensus
	Primary care technologies (TCH)	TCH1. Basic technology	Availability of equipment in primary care	survey; database; expert consensus
	Primary care facility infrastructure (STR)	STR1. Amenities	General service readiness at facility-level	key informant
Model of primary care	Primary care selection of services (SEL)	SEL1. Identifying needs	Population stratification	key informant
		SEL2. Preventive care	Counselling services	key informant
			Population based screening	key informant
			Individual risk assessments/stratification	key informant
			Vaccination services	survey; report; key informant
		SEL3. Diagnostic procedures	Diagnostic exams	key informant
			Final diagnosis in primary care	key informant
		SEL4. Treatment	Prescribing authority of generalist medical practitioners	policy; report; key informant
		SEL5. Management of diseases	Follow-up services in primary care	key informant
			Other services	key informant
		SEL6. Patient engagement	Self-management and health literacy in primary care	key informant
	Primary care design (DES)	DES1. Referral system	Gatekeeping system	survey; report; key informant
			Referral protocol from primary care to higher levels of care	policy; survey; key informant
			Reply and discharge protocol from higher levels of care to primary care	policy; key informant
		DES2. Care pathways	Shared care pathways	report; policy; key informant
		DES3. Flexible access modes	Different access modes	survey; expert consensus
		DES4. Shared care plans	Developing shared care plans	survey; expert consensus
	Primary care workforce organization (ORG)	ORG1. Practice population	Choice of generalist medical practitioner	survey; report; key informant
			Patient list system	policy; report; key informant
			Primary care health professionals’ density	database; report
			Caseload of generalist medical practitioner	database; survey
		ORG2. Out-of-hours care	Opening hours in primary care	report; policy; key informant
			Out-of-hours in primary care	survey; key informant
		ORG3. Primary care teams	Types of primary care facilities	policy; key informant
			Shared practices in primary care	policy; report; database
			Coordination within primary care	policy; survey; report; expert consensus
			Existence of care coordinator	policy; survey; report; expert consensus
		ORG4. Collaboration of primary care with other professionals	Cooperation with specialist medical practitioners	survey; expert consensus
			Coordination across sectors	policy; survey; report; expert consensus
	Primary care services management (MAN)	MAN1. Primary care staffing	Autonomy in staffing of medical staff	key informant
		MAN2. Managing primary care facilities	Degree of autonomy in budgeting	key informant
			Health care technology management	survey; key informant
		MAN3. Strategic planning	Population health management	key informant
	Primary care quality improvement (IMP)	IMP1. National or regional primary care performance assessment	Accountability for performance	policy; survey; report; key informant
			Patient experience measures	policy; survey; report; key informant
			Job satisfaction	survey; key informant
		IMP2. Practice-level quality improvement mechanisms	Quality of care processes	policy; report; key informant
			Safety incidents reporting	policy; report; key informant
		IMP3. External accountability for quality of care	External accountability for quality of care delivered by generalist medical practitioners	key informant
		IMP4. Continuous professional development	Continuous professional development opportunities	database; expert consensus
Care contact	Utilization (UTL)	UTL1. Consultation rate	Overall utilization of primary care services	database; survey
		UTL2. Preventive care and diagnostic services	Influenza vaccination coverage	database; report; expert consensus
			HPV vaccination coverage	database; survey; expert consensus
			Diabetic education	database; survey; expert consensus
			Counselling services for tobacco cessation	survey
			National cancer screening programmes targeting the general population	database; survey; expert consensus
			Individual risk assessments	database; expert consensus
			Tuberculosis preventive care and diagnostic services	database
			WHO recommended rapid test as the initial diagnostic test for tuberculosis	database
	Continuity of primary care (CON)	CON1. Treatment	Hypertension treatment coverage	database; survey; expert consensus
			Tuberculosis treatment coverage	database
			Depression treatment coverage	database; expert consensus
		CON2. Follow-up care	Hypertension follow-up	database; survey; expert consensus
			Diabetes monitoring	database; expert consensus
			Chronic obstructive pulmonary disease follow-up	database; expert consensus
			Post-natal care	database; expert consensus
			Depression treatment follow-up	database; expert consensus
		CON3. Longitudinal continuity of care	Stability of patient–generalist medical practitioner relationship	survey
		CON4. Informational continuity of care	Medical record keeping	survey; expert consensus
			Incoming clinical information procedures	survey; expert consensus
			Generalist–specialist medical practitioner communication	survey; expert consensus
			Generalist medical practitioner-social services	survey; expert consensus
	Coordination of care across settings (COR)	COR1. Transition management	Referral feedback to primary care	survey; expert consensus
	Comprehensiveness of primary care (COP)	COP1. Resolution capacity of generalist medical practitioner	General medical practitioner consultations without referral	database; expert consensus
	People-centredness of primary care (PCC)	PCC1. Patient experience	Patient satisfaction	report; survey
		PCC2. Shared decision-making	Care and treatment shared decision-making	database; survey; report
		PCC3. Patient engagement	Patient reporting opportunity to ask questions	database; survey; report
			Patient reporting enough time with doctor	database; survey; report
			Patient reporting easy to understand explanations	database; survey; report
Primary care outputs	Access to primary care services (ACC)	ACC1. Availability and affordability of primary care services	Same day appointments	survey
			Waiting time for appointment	survey; report
			Access barriers due to treatment costs	database; survey; report
			Access to medicines	database
		ACC2. Acceptability	Patient reported acceptability of primary care services	survey
	Responsiveness of primary care (RES)	RES1. Resolving capacity of primary care	Composite measure	database; survey; report; key informant
	Safety of primary care (SAF)	SAF1. Medical errors	Correct diagnosis	reports; expert consensus
			Incident reporting	reports; expert consensus
		SAF2. Medicine safety	Overall volume of antibiotics prescribed	database
			Medication review	survey; expert consensus
	Effectiveness of primary care services (EFF)	EFF1. Effective management and control of diseases	Control of blood pressure among people treated for hypertension	database; expert consensus
			Control of blood glucose among people treated for diabetes	database; expert consensus
			Tuberculosis detection and treatment	database
			Cancer survival rates	database
Health system outcomes	Quality (QLY)	QLY1. Quality of care for chronic conditions	Hospital admissions for chronic conditions	database
			Avoidable complications	database
			Notified tuberculosis cases lost to follow-up	report
			Stage at diagnosis for cancer	database
		QLY2. Prescribing in primary care	Secondary prevention/high-risk control	survey; expert consensus
			Tuberculosis and rifampicin/multidrug resistant tuberculosis treatment in primary care	database
			Access to palliative care	report; database
	Equity (EQT)	EQT1. Equitable delivery of primary care services	Composite measure	database; survey; report; key informant
	Efficiency (EFC)	EFC1. Unnecessary procedures	Unnecessary duplication of medical tests	survey; expert consensus
Health outcomes	Health status and well-being (HSW)	HSW1. Burden of disease and risk factors	Risk factors – smoking	database
			Risk factors – alcohol	database
			Risk factors – overweight and obesity	database
			Morbidity	database; survey
			Disability adjusted life years	database
		HSW2. Mortality	Standardized death rates	database
			Premature mortality	database

The measurability of each indicator has been safeguarded through an extensive process of identifying a possible source for each. More than half (61%) of the indicators can be sourced from more than one type of data source, increasing the potential measurability across countries. The range of possible data sources include: national and international databases (43 indicators); existing surveys (11 indicators); policies (27 indicators) and reports (10 indicators); and key informants (62 indicators) (Table 3).

## Discussion

### Creating performance intelligence with PHC-IMPACT

This research set out with the aim to create robust performance intelligence for PHC strengthening in Europe. Through the three-staged research process described, the tool has been tailored for a classification, set of tracer conditions and selection of indicators that are sensitive to primary care, policy priorities and information systems in Europe.

The broad suite of indicators is intentional in order to allow the possible tailoring of the indicators, functioning as a menu of options to be selected on the basis of a country’s policy priorities. The customization of the tool is among its core advantages and an important feature to increase the tool’s responsiveness within and transferability across countries. Other unique features are found to include the following: diversified data sources accommodate a range of perspectives for a more holistic view to primary care; the translation and means testing of the tool in Russian attempts to redress context-specific classification challenges unique to the membership of the WHO European Region; the cascading of the taxonomy developed facilitates varied entry points to analysis and a uniquely detailed approach to capture the model of primary care; and, the indicator passports and glossary of key terms developed are a practical resources for data collection.

Prior to the use of the tool, a review of the indicators for further tailoring to the context of a specific country should be conducted. The selection of tracer conditions should also be reviewed, with the possibility to adjust this list given a country’s health priorities. This process of customizing the tool’s suite of indicators should rely on the meaningful engagement of key stakeholders for the full benefits of co-designing with the intended users.

### Addressing classification limitations

The relevance of existing indicators for use in monitoring primary care was a significant limitation. For example, nurses in primary care are not defined in existing databases, surveys or reporting, ultimately limiting the extent to which the number, profile, role and performance of nurses in primary care can be assessed. We have attempted to redress these limitations with adjustments to standard indicators using existing definitions and classifications to improve their sensitivity to primary care. In particular, we have harmonized the varied terminology for the primary care workforce, types of facilities and levels of education that have previously limited the relevance of frameworks and tools across Europe. The glossary of terms developed supports the classification defined [35].

We also found metrics for equity and responsiveness to be limited. A similar finding was reported in a recent review on health system performance assessment frameworks [20]. We recognize the need to operationalize both in a more comprehensive manner, suggesting the use of composite indicators and highlight acceptability as a feature in need of further development. The disaggregation of indicators existing in international databases was retained where possible and includes gender, age, socioeconomic status and rural–urban classifications. These variables are found especially relevant for the analysis of equity. An approach that links the relevant indicators and disaggregations is suggested for a more holistic appraisal of equity considerations.

The tool is found novel in its attempt to capture the capacity of primary care beyond system structures, with indicators like prescribing authority to assess the ability of general medical practitioners to issue initial prescriptions and/or refills for treatment and the autonomy of managers on planning, staffing and budgeting. These features of the model of care, together with the domain on care contact, are found important specifications for depicting primary care that allow for further analysis and comparisons across countries.

To further improve the sensitivity of the tool, answer choices where applicable were disaggregated to capture responses as *country-wide*, *in some regions* or *pilot status*.

### Aligning to information systems and other sources of data for applying PHC-IMPACT

Collecting data should rely as much as possible on existing international databases, surveys and country reporting. The development of an electronic data processing system is already being explored to build linkages to existing databases and create a common living platform for interactive analysis. For other indicators, there is untapped potential to uptake data from national information systems, in particular on hospitalizations for ambulatory sensitive conditions where reporting for many non-OECD, non-EU countries of Europe is out-dated. For these countries, strengthening linkages with national systems should be prioritized.

An electronic questionnaire is being built to serve as a data collection tool for qualitative indicators. Where key informants are a data source, we suggest diversified informants to capture varied profiles (policy, managerial, clinical) for accurate responses on the tool’s range of topics. By soliciting a range of informants with complementary knowledge, this approach also facilitates multi-stakeholder engagement while ultimately yielding more objective and reliable results.

A modular approach could also be taken to data collection for a focus on topics like mental health, maternal and child health, and out-of-hours services based on a country’s priorities. While the tool has prioritized available data, the extent to which indicators are measurable across countries varies making some features more ambitious and future-oriented in some contexts. In instances where preferred databases or survey sources are not available, and the indicator cannot be answered by one informant, we suggest a pragmatic approach is taken using the method of expert consensus. This method is applicable for one quarter (27%) of the total indicator set. Drawing from established group-based methods, a Delphi technique followed by a consensus workshop could be used to generate estimates. The highly structured method preserves anonymity while capturing a range of perspectives. This data could support meaningful analytics as countries aspire to and work in parallel on advancing the use and alignment of data in their national information systems. Lessons on expert consensus methods in health services research could be explored for use here [42–45].

### Policy relevance and research implications

PHC-IMPACT has been designed with view to the information needs of decision-makers in the WHO European Region both for international monitoring and country-specific priority-setting. The merit of a regional approach to monitoring has already been advocated for SDG 3 in the scope of financial protection [46]. We have excluded conditions or services considered a basic expectation or fundamental to primary care, such as family planning and childhood vaccination. The exclusion of these services is a prioritization of the tool’s sensitivity to disaggregate performance in high- and middle-income countries rather than a statement of their importance. Moreover, disease- or service-specific monitoring tools and instruments are in use for this purpose.

The comprehensiveness of the tool’s taxonomy brings depth to the analysis for country-specific use. It is seen as a vehicle for identifying priority areas for improvement, while also shedding light on the information landscape and overall availability of data and actors involved. For analysis purposes, the tool facilitates linkages along different continuums: a clinical continuum, linking prevention, diagnosis, treatment and disease management for tracer conditions; a performance continuum in the classical structure-process-outcome sequence; and a continuum of stakeholders cascading the delivery of services from the micro-level (health professionals, patients), to meso- (managers, regional health authorities) and macro-level (policy-makers, health insurers). These relations between indicators have strong analytical potential to improve the coherence of PHC strengthening and signalling of policy opportunities to accelerate performance gains.

For example, the availability and provision of services in primary care can be assessed across to the care continuum to gauge the extent to which the full continuum of services are available and if not, where gaps in services provision lie (i.e. lack of risk assessment services for cardiovascular diseases or mental health; limited role of primary care in follow-up for tuberculosis. This analysis draws on the indicators related to the selection of services. In a similar approach, the role of different primary care practitioners can be profiled, clustering the provision of services by general medical practitioners, nurses, or specialists working in primary care, among others. This analysis has the potential to shed light on the different roles and scope or practice of primary care practitioners, including important insights into the potential for general medical practitioners to confirm an initial diagnosis or role nurses in risk assessment or follow-up services in primary care. Other thematic clusters of indicators could aggregate indicators by policy-relevant themes such as patient engagement, out-of-hours primary care, integrated health and social care, prescribing practices in primary care, among others still.

The tool should be treated as a living resource to be adjusted and improved upon as new types and sources of data become available. This includes the continuous improvement and development of indicators that are defined according to global standards such as total primary health care expenditure and the UHC services coverage index. It requires piloting beyond the initial country-validation described to test its approach and robustness for country-specific, cross-country comparisons and use overtime. Ultimately, despite its attempt to be comprehensive, not all complexities can be accounted for.

Further to piloting PHC-IMPACT, areas for continued development include: developing composite indicators for features like equity and responsiveness and priority policy areas like scope of practice; selecting a core set of indicators for use in dashboards for the purposes of international benchmarking; improving metrics for hard-to-measure topics, like medicines, primary care workforce and acceptability; exploring methods of expert consensus; and intensifying data collection from existing sources and for newer indicators like patient reported experience measures in primary care. Patient experiences measures are found an important area for further research and development to allow cross-country comparisons with the necessary adjustments for their wider use in eastern European and central Asian countries. Tools developed in the scope of the Patient-Reported Indicators Survey (PaRIS) Initiative of the OECD are one platform that could be coordinated with and tools adapted from for use in the context of countries across the European Region [47].

## Conclusion

Performance intelligence on the ability of primary care to respond to health needs is vital for systems to evolve rather than react to health needs and make sustainable progress towards UHC targets. This paper presents a tool for creating performance intelligence sensitive to the WHO European Region. The tool addresses limitations of existing classifications to better capture primary care, improve linkages with (inter)national information systems, and ensure specificity to high- and middle-income countries. The framework and suite of indicators consolidated have promising analytical power that merits further development through its application.

## Supplementary information

**Additional file 1.** Literature reviewed

**Additional file 2.** Tracer conditions

**Additional file 3.** Indicator passports

**Additional file 4.** Glossary of terms

## Data Availability

All data generated or analysed during this study are included in this published article and its supplementary information files [35].

## Abbreviations

NCDs: noncommunicable diseases
OECD: Organisation for Economic Co-operation and Development
PHC: primary health care
PHC-IMPACT: Primary Health Care Impact, Performance and Capacity Tool
SDGs: Sustainable Development Goals
UHC: universal health coverage
WHO: World Health Organization
