# Supplementary material for: Creating performance intelligence for primary health care strengthening in Europe
Source: BMC Health Serv Res. 2019 Dec 27;19:1006. doi: 10.1186/s12913-019-4853-z (PMC6935208; doi:10.1186/s12913-019-4853-z)
Supplement: Supplementary file 1 — Additional file 1. Literature reviewed [file 12913_2019_4853_MOESM1_ESM.docx]

**Supplementary file 1: Literature reviewed**

**Frameworks and assessment tools**

| **#** | **Short title** |
| --- | --- |
| 1 | Chronic Care Model [1] |
| 2 | Commonwealth Fund framework for a high performance system [2] |
| 3 | Components of Primary Care Instrument (CPCI) [3] |
| 4 | Control knobs framework [4] |
| 5 | Framework for assessing behavioural health care [5] |
| 6 | Framework for describing health care delivery organizations and systems [6] |
| 7 | Health services delivery processes [7] |
| 8 | International Health Partners framework for health [8] |
| 9 | OECD Health Care Quality Indicators Project Framework [9] |
| 10 | Patient centred medical home model [10] |
| 11 | Performance measurement in OECD health systems [11] |
| 12 | Primary Care Assessment Tool (PCAT) [12] |
| 13 | Primary Care Systems Profiles and Performance (PRIMASYS) [13] |
| 14 | Primary Health Care Activity Monitor for Europe (PHAMEU) [14] |
| 15 | Primary Health Care Performance Initiative (PHCPI) [15] |
| 16 | Rapid diagnostic tool with performance measures for services delivery [16] |
| 17 | The Bellagio Model [17] |
| 18 | USAID health systems strengthening assessment tool [18] |
|  | **WHO and regional offices** |
| 19 | European approach to assessing health services delivery performance with ambulatory care sensitive conditions [19] |
| 20 | European framework for health systems strengthening for better noncommunicable disease outcomes [20] |
| 21 | European operational approach to health systems strengthening [21] |
| 22 | European priorities for people-centred health systems [22] |
| 23 | European self-assessment tool for the evaluation of essential public health operations [23] |
| 24 | Framework of indicators and targets for laboratory strengthening under the end TB strategy [24] |
| 25 | Framework on integrated, people-centred health services [25] |
| 26 | Health system performance framework [26] |
| 27 | Health systems building blocks [27] |
| 28 | Health Systems in Transition series framework [28] |
| 29 | Package of essential noncommunicable disease interventions for primary health care [29] |
| 30 | Pan American health Organization framework for integrated health services delivery networks [30] |
| 31 | Performance assessment tool for quality improvement in hospitals (PATH) [31] |
| 32 | Primary Care Evaluation Tool (PCET) [32] |
| 33 | Proposal for monitoring health services assessment personal and non-personal services [33] |
| 34 | Systems-thinking for systems strengthening [34] |
| 35 | Universal health coverage index [35] |

**Surveys**

| **#** | **Short title** |
| --- | --- |
| 1 | Commonwealth Fund International Survey of Primary Care Doctors [36] |
| 2 | Data scanning survey: health services delivery data in the WHO European Region [37] |
| 3 | European Centre for Disease Prevention and Control and Vaccine European New Integrated Collaboration Effort survey on seasonal influenza vaccination [38] |
| 4 | Global survey on eHealth [39] |
| 5 | Health Systems Performance Assessment Working Group on Primary Care Questionnaire [40] |
| 6 | OECD strengthening health information infrastructure for health care quality governance [41] |
| 7 | OECD survey on electronic health records system development and data use [42] |
| 8 | OECD survey on health systems characteristics questionnaire [43] |
| 9 | Patient-Reported Indicators Survey [44] [45, 46] |
| 10 | Quality and Costs of Primary Care in Europe survey (QUALICOPC) [47] |
| 11 | Service availability and readiness assessment [48] [49] |
| 12 | Study on medicines reimbursement policies in Europe [50] |
| 13 | Survey on the assessment of primary care (EU Expert Group on HSPA) [40] |
| 14 | WHO global country capacity and response on noncommunicable disease survey [51] |
| 15 | WHO STEPwise approach to surveillance [52] |
|  |  |

**Databases**

| **#** | **Short title** |
| --- | --- |
| 1 | Antimicrobial medicines consumption network data [53] |
| 2 | CONCORD global surveillance of cancer survival [54] |
| 3 | European Commission European Core Health Indicators (EC-ECHI) [55] |
| 4 | European Commission Eurostat database [56] |
| 5 | European database on human and technical resources for health (HlthRes-DB) [57] |
| 6 | European Detailed Mortality Database (DMDB) [58] |
| 8 | European hospital mortality database (HMDB) [58] |
| 9 | Global health estimates database [11] [59] |
| 10 | Global Health Expenditure database (GHED) [60] |
| 11 | Global Health Observatory [61] [62] |
| 12 | Global reporting on narcotic drugs of the International Narcotics Control Board [63] |
| 13 | Global reporting on tuberculosis [64] |
| 14 | Health 2020 database [65] |
| 15 | Health for All (HFA-DB) [66] |
| 16 | Health Systems and Policy Monitor (HSPM) |
| 17 | International Labour Organization database on earnings and labour costs (ILOSTAT) [67] |
| 18 | OECD health statistics [68] |
| 19 | System of health accounts [69] |
| 20 | Universal health coverage data portal [70] |
| 21 | WHO Essential Medicines and Health Products Price and Availability Monitoring (WHO EMP MedMon) [71] |
| 22 | World Population Prospects Database [72] |

**Report series**

| **#** | **Short title** |
| --- | --- |
| 1 | Health Systems in Transition series [73] |
| 2 | OECD country reviews on health systems [74] |
| 3 | OECD country reviews on national health care quality [75] |
| 4 | WHO European country assessments on ambulatory care sensitive conditions [19] |
| 5 | WHO European country assessments on health systems strengthening for better NCD outcomes [76] |
| 6 | WHO European financial protection country reviews [77] |
| 7 | WHO European series on the organization and provision of primary care [78] |
| 8 | WHO noncommunicable disease country profiles [79] |

**WHO health and development strategies**

| **#** | **Short title** |
| --- | --- |
| 1 | Action plan for sexual and reproductive health [80] |
| 2 | Action plan for the health sector response to HIV in the WHO European Region 2016–2021 [81] |
| 3 | Action plan for the health sector response to viral hepatitis in the WHO European Region 2016–2021 [82] |
| 4 | Action plan for the prevention and control of noncommunicable diseases in the WHO European Region 2016–2025 [83] |
| 5 | European Mental Health Action Plan 2013–2020 [84] |
| 6 | European Vaccine Action Plan 2015–2020 [85] |
| 7 | Global Action Plan for the Prevention and Control of NCDs 2013–2020 [86] |
| 8 | Global Strategy on Human Resources for Health: workforce 2030 |
| 9 | Health 2020: European policy framework for health and well-being [87] |
| 10 | Investing in children: the European child and adolescent health strategy 2015–2020 [88] |
| 11 | Strategy and action plan for healthy ageing in Europe 2012–2020 [89] |
| 12 | Strategy on women’s health and well-being in the WHO European Region 2017–2021 [90] |
| 13 | Sustainable Development Goals [91] |
| 14 | Tuberculosis action plan for the WHO European Region 2016–2020 [92] |
