# Supplementary material for: Creating performance intelligence for primary health care strengthening in Europe
Source: BMC Health Serv Res. 2019 Dec 27;19:1006. doi: 10.1186/s12913-019-4853-z (PMC6935208; doi:10.1186/s12913-019-4853-z)
Supplement: Supplementary file 2 — Additional file 2. Tracer conditions [file 12913_2019_4853_MOESM2_ESM.docx]

**Supplementary file 2: tracer conditions**

| **List of possible tracer conditions** | | | **(i) Priority health area in Europe** | **(ii) Responsiveness to primary health care** | | **(iii) Representativeness of the actions of primary health care (PHC) services across population groups and the life-course** | | | | **Final selection** |
| --- | --- | --- | --- | --- | --- | --- | --- | --- | --- | --- |
| **#** | **Cluster** | **Condition or service** | Priority in current health and development strategies reviewed | Inclusion in PCAMEU^1^ [14] | Classification as ACSC^2^ [19] | **Classification**  Type of condition or service | **Target population group/life stage** | **Gender impor-tance** | **Type of service**  P: prevention; D: detection; T: treatment; M: management | Justification of selection. Rows shaded grey denote conditions proposed for inclusion in framework. |
| 1 | Cancer | cervical | [80, 81, 86, 87, 91] | yes | no | vaccine-preventable | adolescents | women | P, D, M | Selected on basis of prevention-orientation and target to adolescents. |
|  |  | colorectal | [82, 86] | no | no | chronic | older adults | men | D, M | Selected on basis of gender importance in combination with other cancers considered. |
|  |  | breast | [80, 83, 86] | yes | no | chronic | adults | women | D, M | Selected on basis of importance of early detection. |
| 2 | Diabetes | diabetes type II | [83, 86, 87, 91] | yes | yes | chronic | all | both | P, D, T, M | Selected on basis of epidemiological importance and well-established role of PHC across care continuum. |
| 3 | Cardiovascular diseases (CVD) | hypertension | [83, 91, 93] | yes | yes | chronic | adults  older adults | both | P, D, T, M | Selected on basis of epidemiological importance and broad scope of PHC, including status as ACSCs. |
|  |  | angina | [83, 86, 91, 93] | no | yes | chronic | adults  older adults | both | P, D, T, M | Excluded on basis of parsimony of cluster selection and relevant services captured by other selected CVD conditions. |
|  |  | ischemic heart disease | [83, 86, 91, 93] | no | no | chronic | adults  older adults | both | P, D, T, M | Selected on basis of epidemiological importance and relevance to treatment in primary care. |
|  |  | stroke | [83, 86, 91, 93] | no | no | acute | adults  older adults | both | P | Relevant prevention services are captured in the scope of other selected tracer conditions. |
|  |  | congestive heart failure | [83, 86, 91, 93] | no | yes | chronic | adults  older adults | both | P, D, T, M | Excluded on the basis of parsimony and relevant services captured by other CVD conditions. |
| 4 | Vaccine-preventable | influenza | [85, 88, 91, 93] | yes | yes | vaccine-preventable | children  older adults | both | P | Selected on basis of target populations. |
|  |  | hepatitis B | [80-83, 85, 91] | yes | yes | vaccine-preventable | infants | both | P | Excluded on basis of focus on infants, with this target pop. captured elsewhere and priority weighted to childhood. |
| 5 | Communicable diseases | tuberculosis | [81, 86, 91-93] | no | yes | chronic | all | both | P, D, T, M | Selected on basis of ACSC status. |
|  |  | HIV | [80, 81, 91, 92] | yes | no | chronic | all | both | P, D, T, M | Excluded on basis of non-ACSC status and parsimony of cluster selection. |
| 6 | Respiratory | pneumonia | - | no | yes | vaccine-preventable | children  older adults | both | P, D | Excluded on basis of no explicit policy commitment identified despite ACSC status and varied population focus. |
|  |  | chronic obstructive pulmonary disease | [86, 91, 93] | yes | yes | chronic | adults | both | P, D, T, M | Selected on basis of epidemiological importance and overall relevance to PHC. |
|  |  | asthma | [83, 86] | yes | yes | chronic | childhood - onwards | both | P, D, T, M | Selected on basis of epidemiological importance, relevance to PHC, and relevance in childhood. |
| 7 | Ulcers | bleeding or perforated ulcer | - | no | yes | acute | adults | both | P, D | Excluded on basis of no explicit policy commitment identified despite ACSC status. |
|  |  | peptic ulcer | - | yes | no | acute | adults | both | P, D | Excluded on basis of no explicit policy commitment identified. |
| 8 | Mental health | mood disorders, depression and self-harm | [84, 88, 91, 93] | no | yes | chronic | adolescents - onwards | both | P, D, T, M | Excluded on basis of cluster parsimony and challenges of measurability in PHC. |
|  |  | mild depression | [83, 84] | yes | yes | chronic | adolescents - onwards | both | P, D, T, M | Selected on basis of epidemiological importance and overall relevance to PHC. |
| 9 | Ageing | unintended injuries | [89] | no | no | urgent | older adults | both | T | Excluded on basis of narrow scope of PHC services and measurability. |
|  |  | falls ­– prevention | [93] | no | no | service | older adults | both | P | Excluded on basis of narrow scope of PHC services and measurability. |
|  |  | hip replacement | [89] | no | no | service | older adults | both | F | Excluded on basis narrow scope of PHC services and measurability. |
|  |  | dementia | [84, 89] | no | no | chronic | older adults | both | D, T, M | Excluded on basis of measurability. |
|  |  | multiple chronic conditions | [89] | no | no | chronic | older adults | both | P, D, T, M | Excluded on basis of measurability. |
| 10 | Sexual and reproductive health (SRH) | anti-natal care | [80, 91, 93] | yes | no | service | adolescents/  adults | women | P, D, T, M | Excluded on basis of selection of cluster parsimony and priority weighted to service found likely to discern SRH PHC performance in the European context. |
|  |  | family planning | [80, 88, 91] | yes | no | service | adolescents/  adults | both | P | Excluded on basis of selection of cluster parsimony and priority weighted to service found likely to discern SRH PHC performance in the European context. |
|  |  | adolescent health | [80, 88, 91] | no | no | service | adolescents | both | P, D, T, M | Excluded on basis of selection of cluster parsimony and priority weighted to service found likely to discern SRH PHC performance in the European context. |
|  |  | post-natal care | [80] | no | no | service | infant  adolescents/ adults | women infants | T, M | Included on basis of potential to discern PHC performance on SRH services in the context of European countries. |
| 11 | Other | rheumatoid arthritis | - | yes | yes | chronic | older adults | both | P, D, T, M | Excluded on basis of no explicit health priority. |
|  |  | kidney/ urinary tract infections | - | yes | yes | acute | adults | women | P, D, M | Excluded on basis of no explicit health priority. |
|  |  | pelvic inflammatory disease | - | yes | yes | acute | adults | women | P, D, M | Excluded on basis of no explicit health priority. |
|  |  | iron deficiency anaemia | - | yes | yes | chronic | infants  adolescents  adults | Women and infants | P, D, T, M | Excluded on basis of no explicit health priority. |

^1^PHAMEU – Primary Health Care Activity Monitor for Europe; ^2^ACSCs – ambulatory care sensitive conditions
