# Supplementary material for: Creating performance intelligence for primary health care strengthening in Europe
Source: BMC Health Serv Res. 2019 Dec 27;19:1006. doi: 10.1186/s12913-019-4853-z (PMC6935208; doi:10.1186/s12913-019-4853-z)
Supplement: Supplementary file 3 — Additional file 3. Indicator passports [file 12913_2019_4853_MOESM3_ESM.docx]

**Supplementary file 3: Indicator passports**

Note: a definition for underlined terms can be found in Supplementary file 4.

| Domain | Primary care structures |
| --- | --- |
| Subdomain | Primary care governance |
| Feature | Primary care priorities |
| **Indicator/question title** | **Primary care strategy (gov1q1)** |
| Indicator/question definition or question | a. Is there a national primary care strategy? (select all that apply) |
| Numerator/denominator or answer choices | - yes, already published as part of an overall health strategy - yes, already published as a stand-alone strategy - yes, under development as part of an overall health strategy - yes, under development as a stand-alone strategy - no, does not exist or cannot be assessed (exclusive choice) - do not know (exclusive choice) |
| Unit of measurement | categorical |
| Indicator/question definition or question | b. If it has been already published, please provide the weblink and/ or upload the relevant document. |
| Numerator/denominator or answer choices | - weblink |
| Unit of measurement | document upload |
| Indicator/question definition or question | c. If it has already been published, are the goals and targets set out in the strategy being monitored? (select one) |
| Numerator/denominator or answer choices | - yes - no - do not know |
| Unit of measurement | categorical |
| Rationale | Formulating national policies and strategies is a basic function of governments. The task of formulating and implementing a health policy falls within the remit of the Ministry of Health. An explicit primary care strategy signals if primary care is high on the political agenda. It defines the vision for the future and should outline priorities and the expected roles of different actors, inform and build consensus, and estimate the resources required to achieve goals and priorities. Primary care supportive governmental policies are positively associated with access, continuity and coordination of care, the delivery of a wide range of services (in particular preventive care), and better overall health outcomes [1]. |
| Preferred data sources | - review of national health policies - key informant |
| Disaggregation | none specified |
| Limitations | The indicator evaluates whether a policy has been formulated, but not its implementation and/or effectiveness. |
|  |  |
| Domain | Primary care structures |
| Subdomain | Primary care governance |
| Feature | Accountability arrangements |
| **Indicator/question title** | **Primary care mandate (gov2q2)** |
| Indicator/question definition or question | a. Is there a national actor exclusively mandated to support the development of primary care? (select all that apply) |
| Numerator/denominator or answer choices | - yes, a unit/department within the ministry of health (specify name in comments) - yes, a national centre (specify name in comments) - yes, a unit/department within a national centre (specify name in comments) - no (exclusive choice) - do not know (exclusive choice)   comments and clarifications |
| Unit of measurement | categorical |
| Indicator/question definition or question | b. Are there subnational actors mandated to support the development of primary care?   - regional/oblast level (select one) - district level (select one) - municipal level (select one) - other, please specify (select one) |
| Numerator/denominator or answer choices | - yes - no - not applicable - do not know |
| Unit of measurement | categorical |
| Rationale | The creation of a separate primary care unit/department within the ministry of health contributes to a clear mandate for primary care within the ministry nationally and other levels of the health system. Assigning a clear mandate is recognized as a core component of accountability arrangements [2]. Strengthening accountability arrangements nationally can give primary care priority within the ministry, improve relations with other ministries and provide a more systematic and integrated working arrangement [3]. |
| Preferred data sources | - key informant |
| Disaggregation | none specified |
| Limitations | The indicator evaluates whether a unit/department exists but not its impact. |

| Domain | Primary care structures |
| --- | --- |
| Subdomain | Primary care governance |
| Feature | Accountability arrangements |
| **Indicator/question title** | **Primary care resources (gov2q3)** |
| Indicator/question definition or question | a. At the national level, does primary care have a budget that can be distinguished from other levels of care, such as specialist care? (select one) |
| Numerator/denominator or answer choices | yes  no  do not know  comments or clarifications |
| Unit of measurement | categorical |
| Indicator/question definition or question | b. Do sub-national levels have discretion over budgetary decisions/allocations for primary care? (select one) |
| Numerator/denominator or answer choices | yes, at the regional/oblast level  yes, at the district level  yes, at the municipal level  yes, other arrangement, please specify  no  do not know  comments and clarifications |
| Unit of measurement | categorical |
| Rationale | The process of accountability has also been defined beyond the delegation of authority, to include the allocation of resources to carry out the assigned task [4]. The indicator evaluates whether there is local autonomy in terms of authority and financial responsibility for health services [3]. |
| Preferred data sources | - key informant |
| Disaggregation | none specified |
| Limitations | The indicator evaluates whether decentralization is in place however, decentralization pertains to the country’s political situation and varies to a great extent on the country’s size. |

| Domain | Primary care structures |
| --- | --- |
| Subdomain | Primary care governance |
| Feature | Accountability arrangements |
| **Indicator/question title** | **Public health services mandate (gov2q4)** |
| Indicator/question definition or question | Is there an institute/agency carryout the following public health functions?   - surveillance of population health and wellbeing (select one) - monitoring and response to health hazards and emergencies (select one) - health protection including environmental occupational, food safety and others (select one) - health promotion including action to address social determinants and health inequity (select one) - disease prevention, including early detection of illness (select one) - advocacy communication and social mobilization for health (select one) - advancing public health research to inform policy and practice (select one) |
| Numerator/denominator or answer choices | - yes, name of agency/institute - no - do not know |
| Unit of measurement | categorical |
| Rationale | These are core components of the Essential Public Health Operations of the WHO European Action Plan for Strengthening Public Health Capacities and Services [5]. |
| Preferred data sources | - WHO Essential Public Health Operations - key informant |
| Disaggregation | none specified |
| Limitations | The indicator assesses only the scope of the intended core functions but not their actual implementation. |

| Domain | Primary care structures |
| --- | --- |
| Subdomain | Primary care governance |
| Feature | Stakeholder participation and engagement |
| **Indicator/question title** | Roles of professional associations of generalist medical practitioners (gov3q5) |
| Indicator/question definition or question | a. Do legally recognized health professional associations specifically for generalist medical practitioners/family medicine/primary care doctors exist? (select one) |
| Numerator/denominator or answer choices | yes  no  do not know |
| Unit of measurement | categorical |
| Indicator/question definition or question | b. If yes, please provide the name(s) of the association(s), specify at which level the association is active and provide the approximate number of generalist medical practitioners who are members in each of them  Note: if there is more than one association, please answer this question for the three largest |
| Numerator/denominator or answer choices | - name(s) of association - weblink - active at which level: central/federal or state/local? - approximate number of members |
| Unit of measurement | free answer |
| Indicator/question definition or question | c. Were any of these associations involved in the following activities during the previous year?   - national health policy development (select one) - negotiations on pay and working conditions of members (select one) - continuous professional development (select one) - development of undergraduate/bachelor’s education curricula (select one) - development of post-graduate education curricula (select one) - development of clinical practice guidelines and protocols for primary care (select one) |
| Numerator/denominator or answer choices | - yes - no - not applicable - do not know |
| Unit of measurement | categorical |
| Rationale | The existence of organized associations of primary care health professionals (generalist medical practitioners and nurses) is important to advance the development of the profession, to set standards for the quality of services delivery and to safeguard the financial and material interests of the primary care health professionals [94]. Importantly, professional associations refer here to those organizations that represent the interest of health professionals. This is distinguished from health professional regulators representing the interests of patients. To achieve a broad acceptance of primary care reforms, it is important to involve stakeholders in to the policy process and its implementation [95]. |
| Preferred data sources | - key informant |
| Disaggregation | none specified |
| Limitations | There are in many countries a multitude of professional associations that deliver different functions and have different legal status. This measure is limited to legally recognized associations to capture the role of those most prominent in the country. |
|  |  |
| Domain | Primary care structures |
| Subdomain | Primary care governance |
| Feature | Stakeholder participation and engagement |
| **Indicator/question title** | Roles of professional associations of nurses and midwives in primary care (gov3q6) |
| Indicator/question definition or question | a. Do legally recognized health professional associations specifically for nurses and midwives exist? (select one) |
| Numerator/denominator or answer choices | yes, specifically for nurses and midwives in primary care  yes, nurses and midwives in general  yes, both  no  do not know |
| Unit of measurement | categorical |
| Indicator/question definition or question | b. If yes, please provide the name(s) of the association(s), and approximate number of nurses who are members in each of them.  Note: if there is more than one association, please answer this question for the three largest ones |
| Numerator/denominator or answer choices | name(s) of association(s)  weblink  approximate number of members |
| Unit of measurement | categorical |
| Indicator/question definition or question | c. Were any of these associations involved in the following activities during the previous year?  health policy development (select one)  negotiations on pay and working conditions of members (select one)  continuous professional development (select one)  development of undergraduate/bachelor’s education curricula (select one)  development of post-graduate education curricula (select one)  development of clinical practice guidelines and clinical protocols for primary care (select one) |
| Numerator/denominator or answer choices | yes  no  not applicable  do not know |
| Unit of measurement | categorical |
| Rationale | The existence of organized associations or colleges of primary care health professionals (generalist medical practitioners and nurses) is important to advance the development of the profession, to set standards for the quality of health services delivery and to safeguard the financial and material interests of the primary care health professionals [94]. To achieve a broad acceptance of primary care reforms, it is important to involve stakeholders in to the policy process and its implementation [95]. |
| Preferred data sources | - key informant |
| Disaggregation | none specified |
| Limitations | There are in many countries a multitude of professional associations that deliver different functions and have different legal status. This measure is limited to legally recognized associations to capture the role of those most prominent in the country. |

| Domain | Primary care structures |
| --- | --- |
| Subdomain | Primary care governance |
| Feature | Stakeholder participation and engagement |
| **Indicator/question title** | **Roles of patient and/or consumer groups (gov3q7)** |
| Indicator/question definition or question | a. Do any of the following patient and/or consumer health-related groups (associations/organizations) exist as legally recognized entities?  general health-related patient group (select one)  heart disease-specific patient group (select one)  cancer-specific patient group (select one)  diabetes-specific patient group (select one)  tuberculosis-specific patient group (select one)  mental health specific patient group (select one)  consumer group (select one) |
| Numerator/denominator or answer choices | yes  no  do not know |
| Unit of measurement | categorical |
| Indicator/question definition or question | b. Is there a formal role for citizen or patient representatives in the following areas?  health needs assessment and priority setting (select one)  health policy discourse and debate (select one)  licensing of pharmaceuticals (select one)  health technology assessment (select one)  trainings for patients (select one)  membership in primary care advisory boards at the community level (e.g. council boards) (select one)  membership in supervisory boards of primary care facilities (select one) |
| Numerator/denominator or answer choices | yes  no  do not know |
| Unit of measurement | categorical |
| Rationale | Patient engagement is increasingly recognized as an integral part of health services and a critical component of people-centred care. Engaged patients are better able to make informed decisions about their care options. When organized, patients and families can effectively engage in: (i) the design and development of patient-centred processes and system; [61] the development and dissemination of tools, information and educational materials; and (iii) research as a source of data, or co-researchers while contributing to research design or the planning and execution of research [9]. |
| Preferred data sources | - European Patients’ Forum - International Alliance of Patients’ Organizations - OECD Health Systems Characteristics Survey [43] - key informant |
| Disaggregation | none specified |
| Limitations | This indicator measures the existence and intended role but not the actual involvement of patient or consumer associations/organizations/coalitions. |

| Domain | Primary care structures |
| --- | --- |
| Subdomain | Primary care governance |
| Feature | Quality assurance mechanisms |
| **Indicator/question title** | Quality assurance of health professionals (gov4q8) |
| Indicator/question definition or question | a. Who issues licenses/entry to practice for primary care health professionals?  generalist medical practitioners (select one)  nurses (select one) |
| Numerator/denominator or answer choices | government  university  professional regulatory group/body  no mandatory licensure exists  do not know |
| Unit of measurement | categorical |
| Indicator/question definition or question | b. How often is the license renewed?  generalist medical practitioners (select one)  nurses (select one) |
| Numerator/denominator or answer choices | number of years, please specify  it is not time bound  do not know  comments or clarifications |
| Unit of measurement | number of years |
| Indicator/question definition or question | c. If licensure is time bound, which of the following is a requirement for renewal?  generalist medical practitioners (select all that apply)  nurses (select all that apply) |
| Numerator/denominator or answer choices | continuous professional development, please specify number of credit hours in comments  test/examination, please specify frequency in comments  other, please specify  do not know  comments or clarifications |
| Rationale | Recruiting a health workforce based on competencies ensures the selection of candidates with the optimal potential to continuously meet desired competencies and ultimately, the delivery of quality services. Licenses to practice are widely recognized as a mechanism for ensuring quality and strengthening health workforce competencies [94]. For health professionals, it offers a systematic incentive to keep up pre-defined standards of quality, while for the population it provides assurance of health professionals’ competence to practice [95]. |
| Preferred data sources | - review of national health policies - key informant |
| Disaggregation | none specified |
| Limitations | The indicator provides information on the existence of professional licensing but not on the standards of such schemes. |

| Domain | Primary care structures |
| --- | --- |
| Subdomain | Primary care governance |
| Feature | Quality assurance mechanisms |
| **Indicator/question title** | **Quality assurance of facilities (gov4q9)** |
| Indicator/question definition or question | Do the following mechanisms exist for primary care facilities to operate?  licensure (select one)  accreditation (select one)  certification (select one) |
| Numerator/denominator or answer choices | yes, mandatory  yes, voluntary  no  do not know |
| Unit of measurement | categorical |
| Rationale | Licensure, accreditation and certification schemes are key mechanisms for quality improvement of a health system. For the health facilities, they offer a defined minimum standard of quality, while for the population they provide assurance that these minimum standards have been met [3]. |
| Preferred data sources | - review of national health policies - key informant |
| Disaggregation | none specified |
| Limitations | The indicator provides information on the existence of licensure, accreditation and certification but not on the standards of such schemes or their implementation. |

| Domain | Primary care structures |
| --- | --- |
| Subdomain | Primary care governance |
| Feature | Quality assurance mechanisms |
| **Indicator/question title** | Development of primary care clinical practice guidelines (gov4q10) |
| Indicator/question definition or question | a. Are evidence-based national clinical practice guidelines/clinical protocols/standards available for the management (diagnosis and treatment) of the following conditions through a primary health care approach recognized/approved by government or competent authorities?   - cardiovascular disease (select one) - diabetes (select one) - cancer (select one) - chronic respiratory disease (select one) - tuberculosis and latent tuberculosis infection (select one) - mental health condition (select one) |
| Numerator/denominator or answer choices | - yes - no - do not know |
| Unit of measurement | categorical |
| Indicator/question definition or question | b. Where clinical practice guidelines/clinical protocols/standards are available, please indicate whether they contain standard criteria for the referral/referral guidelines from primary care to a higher level of care (secondary/tertiary)?   - cardiovascular disease (select one) - diabetes (select one) - cancer (select one) - chronic respiratory disease (select one) - tuberculosis (select one) - mental health conditions (select one) |
| Numerator/denominator or answer choices | - yes - no - do not know |
| Unit of measurement | categorical |
| Rationale | Clinical protocols and guidelines are systematically developed, evidence-based recommendations that support health professionals and patients to make decisions about the most appropriate, efficient care in specific clinical circumstances [96]. Developing standards and guidelines to support generalist medical practitioners is one of the crucial tools in achieving quality primary care. Guidelines are more likely to be appropriately applied when they are the product of one’s own profession [95]. |
| Preferred data sources | - Country Capacity and Response Survey on Noncommunicable Diseases - review of national health policies - key informant |
| Disaggregation | none specified |
| Limitations | The indicator provides information on the existence of clinical practice guidelines but not on the quality of such guidelines or their use. |

| Domain | Primary care structures |
| --- | --- |
| Subdomain | Primary care governance |
| Feature | Quality assurance mechanisms |
| **Indicator/question title** | **Patient rights and choice (gov4q11)** |
| Indicator/question definition or question | a. Is there a formal definition of patients’ rights at the national level? (select one) |
| Numerator/denominator or answer choices | yes  no  do not know |
| Unit of measurement | categorical |
| Indicator/question definition or question | b. Does the definition include the following rights?  to consent to or to refuse treatment (select one)  to the confidentiality of medical information (select one)  to be informed about relevant risk of medical procedures (select one)  to a second medical opinion (select one)  to access to own medical files (select one)  to raise patient complaints in primary care facilities (select one) |
| Numerator/denominator or answer choices | yes  no  do not know |
| Unit of measurement | categorical |
| Indicator/question definition or question | c. If yes, please provide the weblink and/or the relevant document: |
| Numerator/denominator or answer choices | - weblink |
| Unit of measurement | document upload |
| Rationale | Legislation regarding patients’ rights is important to protect individuals and communities from harm and to safeguard an agreed level of service quality [3]. Patients' rights vary by country and in different jurisdictions, often depending upon prevailing cultural and social norms. Different models of the patient-physician relationship, which can also represent the citizen-state relationship, have been developed, and these have informed the rights to which patients are entitled. There is growing international consensus that all patients have a fundamental right to privacy, to the confidentiality of their medical information, to consent to or to refuse treatment, and to be informed about relevant risk to them of medical procedures [11]. |
| Preferred data sources | OECD Health Care Quality Indicators  review of national health policies  key informant |
| Disaggregation | none specified |
| Limitations | The indicator is not specific to primary care, but the assumption is that patients’ rights are universal to the health system and thus, across levels of care. |

| Domain | Primary care structures |
| --- | --- |
| Subdomain | Primary care financing |
| Feature | Primary care expenditure |
| **Indicator/question title** | **Total primary health care expenditure as a share of total health expenditure (fin1q12)** |
| Indicator/question definition or question | Primary health care expenditure as percent current health expenditure |
| Numerator/denominator or answer choices | as reported in the Global Health Expenditure Database, WHO  PHC%CHE  The numerator includes government and non-government health expenditures, and it is the sum of:   - general outpatient curative care, HC.1.3.1 - dental outpatient curative care, HC.1.3.2 - outpatient curative care, not specified, HC.1.3.nec - home-based curative care, HC.1.4 - outpatient long-term health care, HC.3.3 - home-based long-term health care, HC.3.4 - preventive care, HC.6 - medical goods, HC.5 – 80% - governance, and health system and financing administration HC.7 – 80%   The denominator is the current health expenditures. |
| Unit of measurement | percent |
| Rationale | As a core indicator of health financing systems, this indicator contributes to an understanding of the prioritization in health financing [97]. |
| Preferred data sources | - Global Health Expenditure Database, GHED |
| Disaggregation | none specified |
| Limitations | The System of Health Accounts 2011 standards were not designed to explicitly collect primary health care expenditure information and there is no primary health care expenditure category in its data set. Thus, the estimates are based on the definition for primary health care expenditure based on the System of Health Accounts 2011 expenditure codes of health care functions used in the WHO Global Health Expenditure Database and the limitations of this definition are detailed in that indicator passport. According to the System of Health Accounts 2011, total health expenditure is split into current and capital expenditures. The focus is given to total current expenditures for the purpose of comparison because the capacity to have capital investments varies across countries. Therefore, for this indicator, total current health expenditure is proposed to use for denominator. |
|  |  |
| Domain | Primary care structures |
| Subdomain | Primary care financing |
| Feature | Primary care expenditure |
| **Indicator/question title** | **Domestic primary health care expenditure (fin1q13)** |
| Indicator/question definition or question | a. Domestic general government expenditure on primary health care as a share of overall primary health care expenditure |
| Numerator/denominator or answer choices | as reported in the Global Health Expenditure Database, WHO  GGHE-D_PHC%PHC  The numerator covers expenditure by all domestic public and compulsory sources on primary health care.  Spending on primary care is calculated as the the sum of:   - general outpatient curative care, HC.1.3.1 - dental outpatient curative care, HC.1.3.2 - outpatient curative care, not specified, HC.1.nec - home-based curative care, HC.1.4 - outpatient long-term health care, HC.3.3 - home-based long-term health care, HC.3.4 - preventive care, HC.6 - medical goods, HC.5 – 80% - governance, and health system and financing administration HC.7 – 80%   The denominator is the overall primary health care spending. |
| Unit of measurement | percent |
| Indicator/question definition or question | b. Domestic general government expenditure on primary health care as a share of domestic general government health expenditure |
| Numerator/denominator or answer choices | as reported in the Global Health Expenditure Database, WHO  GGHE-D_PHC%GGHE-D  The indicator covers expenditure by all domestic public and compulsory sources on primary health care.  The numerator is the sum of:   - general outpatient curative care, HC.1.3.1 - dental outpatient curative care, HC.1.3.2 - outpatient curative care, not specified, HC.1.3.nec - home-based curative care, HC.1.4 - outpatient long-term health care, HC.3.3 - home-based long-term health care, HC.3.4 - preventive care, HC.6 - medical goods, HC.5 – 80% - governance, and health system and financing administration, HC.7– 80%   The denominator is the overall domestic general government expenditure on health. |
| Unit of measurement | percent |
| Rationale | Poor financial investment is an impediment to the delivery of primary care [95]. This core health financing indicator reflects a government’s investment in and commitment to primary health care and enables increased accountability of governments to primary health care [97]. It contributes to understanding government prioritization of and commitment to primary health care. |
| Preferred data sources | - System of Health Accounts |
| Disaggregation | none specified |
| Limitations | The System of Health Accounts 2011 standards were not designed to explicitly collect primary health care expenditure information and there is no primary health care expenditure category in its data set. In effect, the estimates generated are based on the definition for primary health care expenditure defined in the System of Health Accounts 2011 expenditure codes of health care functions used in the WHO Global Health Expenditure Database. The limitations this definition are detailed in the indicator passport for the measure: Total primary health care expenditure as a share of total health expenditure (fin1q12). |

| Domain | Primary care structures |
| --- | --- |
| Subdomain | Primary care financing |
| Feature | Primary care expenditure |
| **Indicator/question title** | **Capital and recurrent expenditure arrangements (fin1q14)** |
| Indicator/question definition or question | a. Are there dedicated budget lines for the following type of expenditures?  capital expenditure for primary care (select one)  recurrent expenditure: operations and maintenance for primary care (select one) |
| Numerator/denominator or answer choices | yes  no  do not know |
| Unit of measurement | N/A |
| Indicator/question definition or question | b. What is the level of spending authority for each of the following budget lines?  capital expenditure for primary care (select all that apply)  recurrent expenditure: operations and maintenance for primary care (select all that apply) |
| Numerator/denominator or answer choices | central government  regional/oblast government  district government  municipal government  facility  other  do not know  comments or clarifications |
| Unit of measurement | categorical |
| Indicator/question definition or question | c. Are these allocations earmarked/ring-fenced?  capital expenditure for primary care (select one)  recurrent expenditure: operations and maintenance for primary care (select one) |
| Numerator/denominator or answer choices | yes, for the purchase of specific goods/services  yes, within specific categories of expenditure  no, funds can be (re)allocated without constraint  no, funds can be (re)allocated within certain limits  do not know |
| Unit of measurement | categorical |
| Rationale | Equipping the system with the optimal resources is central to ensuring basic infrastructure, settings, and channels essential to the provision of services are available [14]. The services delivery function relies on the system to support both long-term assets (e.g. facilities, equipment) and short-term operating costs including ordinary repair and maintenance. The availability of these resources is an enabler to the managerial capacity of the services delivery function [15]. |
| Preferred data sources | - key informant |
| Disaggregation | none specified |
| Limitations | none specified |

| Domain | Primary care structures |
| --- | --- |
| Subdomain | Primary care financing |
| Feature | Payment methods in primary care |
| **Indicator/question title** | **Provider payments (fin2q15)** |
| Indicator/question definition or question | a. In which type of settings are primary care services predominantly provided? (select one)  Note: please select only one answer. A similar set of questions follows for the second significant form of services provision, if needed. |
| Numerator/denominator or answer choices | public nurse and midwife office (e.g. health post)  public office of a generalist medical practitioner  public ambulatory generalist practitioners group practice  public ambulatory multi-profile group practice (e.g. polyclinic)  outpatient departments of public hospitals  private nurse and midwife office (e.g. health post)  private office of a generalist medical practitioner  private ambulatory generalist practitioners group practice  private ambulatory multi-profile group practice (e.g. polyclinic)  outpatient departments of private hospitals  other, please specify |
| Unit of measurement | categorical |
| Indicator/question definition or question | b. Do purchasers pay these providers through the following means?  capitation (select one)  fee-for-service (select one)  pay-for-performance (select one)  global budget (select one)  bundled payments (linked to conditions) (select one)  other, please specify in comments (select one) |
| Numerator/denominator or answer choices | yes, country-wide  yes, in some regions (please specify in comments)  yes, is only being piloted  no  do not know  comments or clarifications |
| Unit of measurement | categorical |
| Indicator/question definition or question | c. If capitation is one component of payment, are the following risk factors used for adjustment?  age (select one)  gender (select one)  health status (e.g. measured by prevalence of specific conditions) (select one)  prior use of services (select one)  it is not adjusted (select one)  other, please specify in comments |
| Numerator/denominator or answer choices | yes, country-wide  yes, in some regions (please specify in comments)  yes, is only being piloted  no  do not know  comments or clarifications |
| Unit of measurement | categorical |
| Indicator/question definition or question | d. Please indicate the second most predominant form of services provision: (select one) |
| Numerator/denominator or answer choices | public nurse and midwife office (e.g. health post)  public office of a generalist medical practitioner  public ambulatory generalist practitioners group practice  public ambulatory multi-profile group practice (e.g. polyclinic)  outpatient departments of public hospitals  private nurse and midwife office (e.g. health post)  private office of a generalist medical practitioner  private ambulatory generalist practitioners group practice  private ambulatory multi-profile group practice (e.g. polyclinic)  outpatient departments of private hospitals  other, please specify  there is no second significant form of service provision |
| Unit of measurement | categorical |
| Indicator/question definition or question | e. Do purchasers pay these providers through the following means?  capitation (select one)  fee-for-service (select one)  pay-for-performance (select one)  global budget (select one)  bundled payments (linked to conditions) (select one)  other, please specify in comments (select one) |
| Numerator/denominator or answer choices | yes, country-wide  yes, in some regions (please specify)  yes, is only being piloted  no  do not know  comments or clarifications |
| Unit of measurement | categorical |
| Indicator/question definition or question | f. If capitation is one component of payment, are the following risk factors used for adjustment?  age (select one)  gender (select one)  health status (e.g. measured by prevalence of specific conditions) (select one)  prior use of services (select one)  it is not adjusted (select one)  other, please specify in comments |
| Numerator/denominator or answer choices | yes, country-wide  yes, in some regions (please specify)  yes, is only being piloted  no  do not know  comments or clarifications |
| Unit of measurement | categorical |
| Rationale | The organisation of health services resources has the potential to influence the accessibility of health services, their effectiveness, efficiency and quality, as well as health professionals’ and patients’ satisfaction. Generally, group practices are deemed to increase patient accessibility and professional working conditions, as well as the effectiveness and efficiency of health care delivery as several health professionals work together in collaboration. Furthermore, the public/private mix of institutions delivering health services is often considered to be an important feature of the health systems since: (i) they respond to different motivations and face distinct constrains leading to variations in efficiency in the delivery of care; and [61] integrated public health services may be more receptive to command-and-control regulation from public authorities [16]. Flexible blended payment methods produce a desirable mix of incentives that can change professional behaviour, improve the quality of care and reduce inequalities in the delivery of services [3]. |
| Preferred data sources | OECD Health Systems Characteristics Survey  Health Systems in Transition series  key informant |
| Disaggregation | - - rural-urban |
| Limitations | none specified |
|  |  |
| Domain | Primary care structures |
| Subdomain | Primary care financing |
| Feature | Payment methods in primary care |
| **Indicator/question title** | **Employment status and remuneration of generalist medical practitioners (fin2q16)** |
| Indicator/question definition or question | a. What is the predominant employment status of the generalist medical practitioners supplying primary care services? (select one) |
| Numerator/denominator or answer choices | self-employed  employed in the public sector  privately employed  do not know |
| Unit of measurement | categorical |
| Indicator/question definition or question | b. How are these generalist medical practitioners remunerated? (select one) |
| Numerator/denominator or answer choices | salary  fee-for-services  capitation  mix of salary and capitation  mix of fee-for-service and capitation  mix of fee-for-service and salary  mix of salary, fee-for-service and capitation  do not know |
| Unit of measurement | categorical |
| Rationale | Flexible blended payment methods produce a desirable mix of incentives that can change professional behaviour, improve the quality of care and reduce inequalities in delivery of services [3]. Provider payment arrangements affect the quantity, quality and efficiency of health services, each payment scheme providing specific incentives. For example, fee-for-services favours both quantity and quality, but can lead to supplier-induced demand. Whereas, prospective payments and capitation can lead providers to reduce their effort, select healthier patients and over-refer to other sectors of care [16], [17]. |
| Preferred data sources | OECD Health Systems Characteristics Survey  Health Systems in Transition series  key informant |
| Disaggregation | none specified |
| Limitations | none specified |
|  |  |
| Domain | Primary care structures |
| Subdomain | Primary care financing |
| Feature | Payment methods in primary care |
| **Indicator/question title** | **Pay-for-performance (fin2q17)** |
| Indicator/question definition or question | a. Can primary care providers (health professionals or practices) get a bonus payment for achieving targets (pay-for-performance)? (select one) |
| Numerator/denominator or answer choices | - yes, country-wide - yes, in some regions (please specify) - yes, is only being piloted - no - do not know |
| Unit of measurement | categorical |
| Indicator/question definition or question | If yes, please provide information for the largest pay-for-performance scheme for items b-e: |
| Indicator/question definition or question | b. Is participation mandatory or voluntary? (select one) |
| Numerator/denominator or answer choices | - mandatory for all primary care providers country-wide - mandatory for subset of primary care providers (e.g. a region, rural, pilot) - voluntary and open to all primary care providers - voluntary but subject to some conditions (e.g. accreditation, practice size, geography) - do not know |
| Unit of measurement | categorical |
| Indicator/question definition or question | c. For those providers participating in the programme(s), if targets apply to receive bonus/payment, please specify the criteria (e.g. targets for screening or vaccination rate, the follow-up of individuals with chronic diseases, referral rates below a certain level, patient satisfaction, share of generics in prescriptions, etc.) |
| Numerator/denominator or answer choices | comment |
| Unit of measurement | free answer |
| Indicator/question definition or question | d. Who is the bonus/payment normally paid to? (select one) |
| Numerator/denominator or answer choices | - directly to individual health professionals - provider institutions, which then have a large degree of freedom to determine how payments are used (primary care facility) - other, please specify - do not know |
| Unit of measurement | categorical |
| Rationale | While rigorous systematic reviews of pay-for-performance programmes show that pay-for-performance does not lead to 'breakthrough' quality improvements, and measures and other key building blocks of the programmes can be highly inadequate, pay-for-performance can have a boarder role serving as an instrument for improving health system governance and strategic health purchasing, and an impact on the relationship between purchasers and providers by supporting discussion of provider payment reform, quality measurement, and accountability for outcomes [98],[18]. |
| Preferred data sources | - OECD Health Systems Characteristics Survey - key informant |
| Disaggregation | none specified |
| Limitations | none specified |
|  |  |
| Domain | Primary care structures |
| Subdomain | Primary care financing |
| Feature | Payment methods in primary care |
| **Indicator/question title** | **Support for caregivers/family carers (informal sector) (fin2a18)** |
| Indicator/question definition or question | Is the following support available for carers/family carers?   - in cash (e.g. care allowance, paid care leave, attendance allowance) (select one) - in kind (e.g. vouchers, respite services, social insurance contributions, unpaid care leave, day/night care services, community care services in general) (select one) |
| Numerator/denominator or answer choices | - yes, country-wide - yes, in some regions (please specify) - yes, is only being piloted - no - do not know |
| Unit of measurement | categorical |
| Rationale | Putting an appropriate mix of services in place, including support for informal care, is key to making health and long-term care systems sustainable in the future. Supporting informal caregivers, including providing training and protecting their physical and mental well-being contributes positively to outcomes for the health of caregivers and the people for whom they care. Financial support and social security benefits to these caregivers have been recognized as a means to support carers/family carers [18], [19]. |
| Preferred data sources | - Health Systems in Transition series - key informant |
| Disaggregation | none specified |
| Limitations | Comparability across settings may be challenging if, for example, monetary benefits and reimbursement schemes vary widely, so some unit of standardisation might be needed. The indicator assesses on some of the known mechanisms to support informal caregivers/family carers. |
|  |  |
| Domain | Primary care structures |
| Subdomain | Primary care coverage of services |
| Feature | Benefit package |
| **Indicator/question title** | **Services included in the health benefit package (fin3q19)** |
| Indicator/question definition or question | a. Are the following services included in the health benefit package?   - outpatient consultations/visits: generalist medical practitioners office consultations/visits (select one) - outpatient consultations/visits: generalist medical practitioners home consultations/visits (select one) - outpatient consultations/visits: allied health professionals (select one) - outpatient consultations/visits: specialist medical practitioners (select one) - diagnostic tests: laboratory tests (select one) - diagnostic tests: imaging (select one) - outpatient prescription medicines – prescribed in primary care (select one) |
| Numerator/denominator or answer choices | - yes, free at the point of care - yes, subject to a fixed co-payment per service - yes, subject to a co-payment as a percentage of the price of the service - no, are not part of the benefit package - do not know   comments or clarifications |
| Unit of measurement | categorical |
| Indicator/question definition or question | b. If the service is not free at the point of care, for which of the following segments of the population are there exemptions?   - outpatient consultations/visits: generalist medical practitioners office consultations/visits (select all that apply) - outpatient consultations/visits: generalist medical practitioners home consultations/visits (select all that apply) - outpatient consultations/visits: allied health professionals (select all that apply) - outpatient consultations/visits: specialist medical practitioners (select all that apply) - diagnostic tests: laboratory tests (select all that apply) - diagnostic tests: imaging (select all that apply) - outpatient prescription medicines – prescribed in primary care (select all that apply) |
| Numerator/denominator or answer choices | - those with certain medical conditions - those with disabilities - low-income people - beneficiaries of social benefits - seniors - children under a specific age, please specify - pregnant women - unemployed, please specify conditions - families of unemployed, please specify conditions - others, please specify |
| Unit of measurement | categorical |
| Rationale | Formulating a service package and defining entitlements is a basic process of the health services delivery function [7]. The exercise of specifying a core package of entitlements is a value-laden process, looking to decision-makers and system stewards to establish a strategic policy position and equitable framework for protected access to health services when faced with competing priorities. |
| Preferred data sources | - OECD Health Systems Characteristics Survey - WHO Regional Office for Europe: Can people afford to pay for health care series - key informant |
| Disaggregation | none specified |
| Limitations | none specified |

| Domain | Primary care structures |
| --- | --- |
| Subdomain | Primary care workforce |
| Feature | Primary care workforce planning |
| **Indicator/question title** | Type of primary care health professionals (wrk1q20) |
| Indicator/question definition or question | 1. Does a regulation specifying the health professionals working in primary health care exist? (select one) |
| Numerator/denominator or answer choices | - yes, please specify name, number, weblink in comments - no - do not know   comments or clarifications |
| Unit of measure | categorical |
| Indicator/question definition or question | 1. According to this regulation, do the following health professionals work in primary care? If there is no regulation in place, please specify in general.  - general medical practitioner/family medicine doctor (select one) - district therapeutist (select one) - district paediatric doctor (as a generalist medical practitioner) (select one) - feldscher (select one) - midwife (health professional / associate professional) (select one) - nurse (health professional / associate professional) (select one) (please specify) - social worker (select one) - psychologist (select one) - narrow specialist (select one) - paediatrician (specialist) (select one) - specialist medical practitioner (select one) (please specify) - physiotherapist in ambulatory settings (select one) - dietician and nutritionist (select one) - occupational therapist (select one) - speech therapist (select one) - dentist (select one) - pharmacist (select one) - public health professional (please specify) (select one) - other (select one) (please specify)   comments or clarifications (please specify if in practice, not bound by regulation, any other health professionals work in primary care) |
| Numerator/denominator or answer choices | yes  no  do not know |
| Unit of measurement | categorical |
| Rationale | Having a general medical practitioner rather than a specialist medical practitioner as a regular source of care has been associated with better health outcomes and lower health care costs. Greater supply of specialty physicians is consistently associated with better health outcomes. Nursing disciplines and allied health professionals perform services that address health risk behaviours more often than physicians [95]. |
| Preferred data sources | - review of national health policies - key informant |
| Disaggregation | none specified |
| Limitations | The indicator does not provide information on the exact duties outlined for primary care health professionals. |
|  |  |
| Domain | Primary care structures |
| Subdomain | Primary care workforce |
| Feature | Primary care workforce planning |
| **Indicator/question title** | **Scope of practice for primary care health professionals (wrk1q21)** |
| Indicator/question definition or question | a. Have tasks/duties been formally defined, by the government or professional bodies, for the following primary care health professionals?  generalist medical practitioner (select one)  nurse (health professional) (select one)  nurse (associate professional) (select one)  feldscher/paramedical practitioner (select one) |
| Numerator/denominator or answer choices | yes  no  not applicable  do not know  comments or clarifications |
| Unit of measurement | categorical |
| Indicator/question definition or question | b. If yes, please provide the weblink and/or upload the relevant document. |
| Numerator/denominator or answer choices | weblink |
| Unit of measurement | document upload |
| Rationale | Legal reference to the tasks/duties of generalist medical practitioners gives formal recognition to the profession as a specific discipline and influences the position it takes in a health system [95]. |
| Preferred data sources | review of national health policies  key informant |
| Disaggregation | none specified |
| Limitations | The indicator does not provide information on the exact duties outlined for primary care health professionals. |

| Domain | Primary care structures |
| --- | --- |
| Subdomain | Primary care workforce |
| Feature | Primary care workforce planning |
| **Indicator/question title** | Incentives for recruitment and retention in underserved areas (wrk1q22) |
| Indicator/question definition or question | a. Do the following mechanisms to encourage generalist medical practitioners to work in underserved, remote and/or rural areas exist?   - compulsory service requirements in rural and remote areas (select one) - scholarships, bursaries or other education subsidies with enforceable agreements of return of service in rural or remote areas (select one) - financial incentives (e.g. hardship allowances, grants for housing, free transportation, paid vacation, grants for education of dependents) to outweigh the opportunity costs associated with working in rural areas (select one) - other, please specify in comments |
| Numerator/denominator or answer choices | yes, country-wide  yes, in some regions (please specify)  yes, is only being piloted  no  do not know  comments or clarifications |
| Unit of measurement | categorical |
| Indicator/question definition or question | b. Do the following mechanisms to encourage nurses with a post-graduate degree (practicing in primary care) to work in underserved, remote and/or rural areas exist?   - compulsory service requirements in rural and remote areas (select one) - scholarships, bursaries or other education subsidies with enforceable agreements of return of service in rural or remote areas (select one) - financial incentives (e.g. hardship allowances, grants for housing, free transportation, paid vacation, grants for education of dependents) to outweigh the opportunity costs associated with working in rural areas (select one) - other, please specify in comments |
| Numerator/denominator or answer choices | yes, country-wide  yes, in some regions (please specify)  yes, is only being piloted  no  do not know  comments or clarifications |
| Unit of measurement | categorical |
| Rationale | One of the most consistent policy characteristics in countries with a strong primary care system is the government’s attempts to distribute resources equitably [95]. Resolution WHA64.6 calls "to develop strategies and policies to increase the availability of motivated and skilled health professionals in remote and rural areas, with reference to WHO global policy recommendations on increasing access to health professionals in remote and rural areas through improved retention of the health workforce" [99]. These are a set of evidence-based WHO recommendations on how to improve the recruitment and retention of health professionals in underserved areas [100]. |
| Preferred data sources | - review of national health policies - key informant |
| Disaggregation | This indicator is part of the equity component. |
| Limitations | none specified |

| Domain | Primary care structures |
| --- | --- |
| Subdomain | Primary care workforce |
| Feature | Primary care workforce planning |
| **Indicator/question title** | **Retraining programme for specialist medical practitioners/narrow specialists (wrk1q23)** |
| Indicator/question definition or question | a. Is there a retraining programme for specialist medical practitioners/narrow specialists to work as generalist medical practitioners? (select one) |
| Numerator/denominator or answer choices | - yes, part of a regular program   yes, according to assessments/needs/planning  no  do not know |
| Unit of measurement | categorical |
| Indicator/question definition or question | b. If yes, how long is the retraining programme (full-time equivalent)? |
| Numerator/denominator or answer choices | number of months  do not know |
| Unit of measurement | number of months |
| Indicator/question definition or question | c. If yes, how many specialist medical practitioners have been retrained into generalist medical practitioners in the most recent year? |
| Numerator/denominator or answer choices | number of physicians  do not know |
| Unit of measurement | number of physicians |
| Rationale | A health workforce in sufficient quantity and equipped with adequate competencies is critical for improving outcomes for patients and populations [101]. Health workforce planning and forecasting and training programmes are an integral process for anticipating a workforce capable of performing tasks that meet future health demands [102]. |
| Preferred data sources | key informant  database |
| Disaggregation | none specified |
| Limitations | none specified |

| Domain | Primary care structures |
| --- | --- |
| Subdomain | Primary care workforce |
| Feature | Primary care workforce planning |
| **Indicator/question title** | Workforce registry with information on primary care professionals (wrk1q24) |
| Indicator/question definition or question | Do health workforce registries currently exist with information specifically for:  generalist medical practitioners? (select one)  nurses specifically working in primary care? (select one) |
| Numerator/denominator or answer choices | yes, electronic  yes, paper  no  do not know |
| Unit of measurement | categorical |
| Rationale | A workforce registry contributes accurate and timely health workforce data which is crucial for health workforce planning, training, improving regulation of practice, quality of care and easy access to information on the production, distribution and utilization of health professionals [103]. The global strategy on human resources for health: Workforce 2030 calls for all Member States to have health professional registers by year 2030 [104]. |
| Preferred data sources | - Availability of national health services delivery data across the WHO European Region: scanning survey results [37] - key informant |
| Disaggregation | none specified |
| Limitations | The indicator measures the existence of a registry and not its quality regarding accuracy, completion etc. |

| Domain | Primary care structures |
| --- | --- |
| Subdomain | Primary care workforce |
| Feature | Financial status of generalist medical practitioners |
| **Indicator/question title** | **Relative financial status of generalist medical practitioners (wrk2q25)** |
| Indicator/question definition or question | a. Relative financial status of generalist medical practitioners vs. average gross annual income of employees in the economy |
| Numerator/denominator or answer choices | **Numerator:** average gross annual income (full-time equivalent) of generalist medical practitioners including social security contributions and income taxes payable by the employee (exclude practice expenses for self-employed doctors)  **Denominator:** average gross annual income (full-time equivalent) of employees in the economy in local currency |
| Unit of measurement | ratio |
| Indicator/question definition or question | b. Relative financial status of generalist medical practitioners vs. specialist medical practitioners |
| Numerator/denominator or answer choices | **Numerator:** average gross annual income (full-time equivalent, in local currency) of generalist medical practitioners including social security contributions and income taxes payable by the employee (exclude practice expenses for self-employed doctors)  **Denominator:** average gross annual income (full-time equivalent, in local currency) of specialist medical practitioner/cardiologist, including social security contributions and income taxes payable by the employee (exclude practice expenses for self-employed doctors |
| Unit of measurement | ratio |
| Rationale | The ratio of average gross annual income of generalist medical practitioner to (i) average wage of full-time employees in all sectors in the country, and [61] specialist medical practitioner, can be used to evaluate the financial attractiveness of a generalist medical practitioner. In many countries, governments influence the level and structure of physician remuneration by being one of the main employers of physicians or purchaser of their services, or by regulating their fees [105]. Poor financial investment and discouraging health professional salaries are among the impediments to delivery of primary care. |
| Preferred data sources | - International Labour Organization for average gross annual income of employees in the economy - OECD – StatHealth (13 countries, dataset: health care resources, remuneration of general practitioners, remuneration of specialists, no disaggregation) - national database – human resources |
| Disaggregation | - rural-urban - gender |
| Limitations | none specified |
|  |  |
| Domain | Primary care structures |
| Subdomain | Primary care workforce |
| Feature | Primary care workforce availability |
| **Indicator/question title** | **Age distribution of generalist medical practitioners (wrk3q26)** |
| Indicator/question definition or question | Age distribution of practising generalist medical practitioners |
| Numerator/denominator or answer choices | **Numerator:** number of practising generalist medical practitioners with a given characteristic:  <34  35-44  45-54  55-64  >=65  **Denominator:** total number of practising generalist medical practitioners (the number should be at the end of the calendar year)  Note: the data should be provided for practising generalist medical practitioners, if not possible the data can be reported for professionally active generalist medical practitioners or generalist medical practitioners licensed to practise. |
| Unit of measurement | percent |
| Rationale | The key to maintaining a sufficient workforce, in the face of the impending retirement of the ‘baby boom’ generation, is to educate, recruit and retain young practitioners while reinvesting in a mature workforce [95]. This indicator is included among core health workforce indicators of the framework ‘Monitoring the Building Blocks of Health Systems’ [106]. |
| Preferred data sources | - registries of health professionals - health facility staffing routine data |
| Disaggregation | none specified |
| Limitations | none specified |

| Domain | Primary care structures |
| --- | --- |
| Subdomain | Primary care workforce |
| Feature | Academic status of primary care |
| **Indicator/question title** | General practice/family medicine undergraduate/bachelor education (wrk4q27) |
| Indicator/question definition or question | Is there a mandatory full course on general practice/family medicine as part of the undergraduate/bachelor’s medical education curriculum for all students? (select one) |
| Numerator/denominator or answer choices | yes, please specify number of hours  no  do not know |
| Unit of measurement | categorical |
| Rationale | Despite the well-recognized importance of general practice/family medicine in medical education, undergraduate training remains widely based on disciplines other than general practice/family medicine [107],[108]. Increasing training in undergraduate medical education on general practice/family medicine ensures the exposure of students to the discipline and ultimately, contributes to the availability of skilled and qualified health professionals [95]. |
| Preferred data sources | - key informant |
| Disaggregation | none specified |
| Limitations | This indicator determines the existence of training and its length but does not consider the actual contents or quality of the training provided. |
|  |  |
| Domain | Primary care structures |
| Subdomain | Primary care workforce |
| Feature | Academic status of primary care |
| **Indicator/question title** | General practice/family medicine postgraduate education (wrk4q28) |
| Indicator/question definition or question | Is there a postgraduate specialization (specialty) in general practice/family medicine? (select one) |
| Numerator/denominator or answer choices | yes, please specify the duration of the programme in years  no  do not know |
| Unit of measurement | categorical |
| Rationale | The establishment of general practice/family medicine postgraduate training works to strengthen the position of general practice/family medicine in academics and the overall development of the discipline [95]. To this end, international standards for postgraduate general practice/family medicine education have been developed [109]. |
| Preferred data sources | - key informant |
| Disaggregation | none specified |
| Limitations | This indicator determines the existence of training and its length but does not consider the actual contents or quality of the training provided. |
|  |  |
| Domain | Primary care structures |
| Subdomain | Primary care workforce |
| Feature | Academic status of primary care |
| **Indicator/question title** | General practice/family medicine postgraduate clinical practice (wrk4q29) |
| Indicator/question definition or question | Do general practice/family medicine trainees spend time practicing in a primary care facility during postgraduate education programme? (select one) |
| Numerator/denominator or answer choices | yes, please specify the duration of the practice in hours  no  do not know |
| Unit of measurement | categorical |
| Rationale | During initial education, students should apply the competencies that they will be required use in clinical settings. It is well recognized that while students learn by abstraction and through lectures, they should also practice in clinical settings under the supervision of certified and practicing health professionals [94]. This exposure and evaluation of required competencies during initial education should be an important criterion for certification and professional registration prior to entering the workforce. |
| Preferred data sources | - key informant |
| Disaggregation | none specified |
| Limitations | none specified |

| Domain | Primary care structures |
| --- | --- |
| Subdomain | Primary care workforce |
| Feature | Academic status of primary care |
| **Indicator/question title** | **General practice/family medicine specialization among medical students (wrk4q30)** |
| Indicator/question definition or question | Percent of students graduating from an undergraduate/bachelor’s programme in medicine that enrol in general practice/family medicine specialization |
| Numerator/denominator or answer choices | **Numerator:** number of individuals in the denominator that choose a general practice/family medicine specialization  **Denominator:** total number of students graduating from an undergraduate/bachelor’s programme in a reference year |
| Unit of measurement | percent |
| Rationale | A greater supply of primary care providers, as opposed to a greater supply of specialty physicians, is consistently associated with better health outcomes [95]. |
| Preferred data sources | - routine administrative records of education institutions |
| Disaggregation | none specified |
| Limitations | none specified |

| Domain | Primary care structures |
| --- | --- |
| Subdomain | Primary care workforce |
| Feature | Academic status of primary care |
| **Indicator/question title** | Nurses working in primary care undergraduate/bachelor and postgraduate education (wrk4q31) |
| Indicator/question definition or question | a. Do the following degree programmes exist for nurses? (select one)  vocational training  undergraduate/bachelor’s programme  undergraduate/bachelor’s programme + 1 year postgraduate education programme  undergraduate/bachelor’s programme + 2 years or more postgraduate education programme |
| Numerator/denominator or answer choices | yes  no  do not know |
| Unit of measurement | categorical |
| Indicator/question definition or question | b. If yes, can students specialize in primary care during the following education programmes? (select one)  vocational training  undergraduate/bachelor’s programme  undergraduate/bachelor’s programme + 1 year postgraduate education programme  undergraduate/bachelor’s programme + 2 years or more postgraduate education programme |
| Numerator/denominator or answer choices | yes  no  do not know |
| Unit of measurement | categorical |
| Rationale | The existence of a undergraduate and post-graduate programme in nursing/midwifery contributes to the availability of skilled and qualified health care providers which is a key quality determinant [95]. Appropriately educated nurses working in advanced practice have been shown to provide services of equal quality to physicians [110]. |
| Preferred data sources | - key informant |
| Disaggregation | none specified |
| Limitations | none specified |
|  |  |
| Domain | Primary care structures |
| Subdomain | Primary care workforce |
| Feature | Academic status of primary care |
| **Indicator/question title** | Professional journal on general practice/family medicine (wrk4q32) |
| Indicator/question definition or question | Is there a peer-reviewed journal on general practice/family medicine/primary health care, recognized as a scientific journal in the country and being published in one of your country’s official languages? (select one) |
| Numerator/denominator or answer choices | yes, name and weblink  no  do not know |
| Unit of measurement | categorical |
| Rationale | The existence of a peer reviewed journal is an important contributor to the successful scientific progress of primary care [95]. |
| Preferred data sources | - key informant |
| Disaggregation | none specified |
| Limitations | none specified |

| Domain | Primary care structures |
| --- | --- |
| Subdomain | Primary care information systems |
| Feature | Data capture |
| **Indicator/question title** | Electronic health records system (inf1q33) |
| Indicator/question definition or question | a. Does the health information system contain individual records for primary care services? (select one) |
| Numerator/denominator or answer choices | yes, currently electronic  yes, currently in transition from paper-based to electronic  yes, currently paper-based  no  do not know |
| Unit of measurement | categorical |
| Indicator/question definition or question | b. Do individual records contain information on socio-economic determinants? (e.g. education, employment status, family status, etc.) (select one) |
| Numerator/denominator or answer choices | yes  no  do not know |
| Unit of measurement | categorical |
| Indicator/question definition or question | c. Is a unique patient identification number used in primary care? (select one) |
| Numerator/denominator or answer choices | yes  no  do not know |
| Unit of measurement | categorical |
| Rationale | Electronic health record systems can enable individuals to have an electronic record of their key characteristics and health concerns, as well as their history of encounters with the health system and the treatments that they have received from a variety of health providers. This record can then be shared with health providers to support the provision of the most appropriate care. The existence of such records opens a promising new frontier for advancing patient care, in the same way that advancements in the use of information technologies have revolutionised most other industries. Unique patient identifiers are crucial to the development of longitudinal electronic health records, to ensure that the data within the record is complete and accurate, as patients move among health care providers, health insurers, and regions within their country and over time. They are also important for statistical purposes to identify unique patients and to conduct, where approved, linkages of data across more than one data source [111]. |
| Preferred data sources | - Strengthening health information infrastructure for health care quality governance [41] - Availability of national health services delivery data across the WHO European Region: scanning survey results [37] - key informant |
| Disaggregation | none specified |
| Limitations | none specified |
|  |  |
| - Domain | Primary care structures |
| Subdomain | Primary care information systems |
| Feature | Data capture |
| **Indicator/question title** | Electronic health record system linked to clinical systems (inf1q34) |
| Indicator/question definition or question | Do electronic health records link to any of the following?  automatic vaccination alerting systems (select one)  pathology information systems (select one)  picture archiving and communication systems (select one)  pharmacy information systems (select one)  laboratory information systems (select one) |
| Numerator/denominator or answer choices | yes, country-wide  yes, in some regions (please specify)  yes, in some facilities  yes, is only being piloted  no  do not know |
| Unit of measurement | categorical |
| Rationale | Computerization of practices is becoming increasingly important in primary care for the practice of evidence-based medicine, learning and knowledge management and quality improvement processes. Effective use of computerization applications is beneficial for the efficiency and quality of care [95]. |
| Preferred data sources | - WHO global survey on eHealth [39] - key informant |
| Disaggregation | none specified |
| Limitations | none specified |
|  |  |
| Domain | Primary care structures |
| Subdomain | Primary care information systems |
| Feature | Aggregation of data |
| **Indicator/question title** | Patient registries (inf2q35) |
| Indicator/question definition or question | a. Do the following national patient registries exist?  cardiovascular disease (select one)  cancer (select one)  diabetes (select one)  respiratory disease (select one)  tuberculosis (select one) |
| Numerator/denominator or answer choices | yes, electronic  yes, paper-based  no  do not know |
| Unit of measurement | categorical |
| Indicator/question definition or question | b. Do individual records contain information on socio-economic determinants? (e.g. education, employment status, family status, etc.)  cardiovascular disease (select one)  cancer (select one)  diabetes (select one)  respiratory disease (select one)  tuberculosis (select one) |
| Numerator/denominator or answer choices | yes  no  do not know |
| Unit of measurement | categorical |
| Indicator/question definition or question | c. Is a unique patient identification number used in registries?  cardiovascular disease (select one)  cancer (select one)  diabetes (select one)  respiratory disease (select one)  tuberculosis (select one) |
| Numerator/denominator or answer choices | yes  no  do not know |
| Unit of measurement | categorical |
| Rationale | Information technology is commanding an increasingly important role in the health care arena. Electronic patient registries can signal and update the workforce about care plans, remind them of outreach efforts, and help monitor responses to treatment. Even simple information systems, if designed properly, can serve the same basic functions as sophisticated systems by monitoring the incidence and prevalence of conditions in the clinical population, monitoring individual patients’ treatment and outcomes, and reminding providers about care plans [112]. |
| Preferred data sources | - WHO Global Country Capacity and Response Survey on Noncommunicable Diseases Survey 2017 - Availability of national health services delivery data across the WHO European Region: scanning survey results [37] - key informant |
| Disaggregation | none specified |
| Limitations | The indicator determines the existence of patient registries in general and therefore is not specific to primary care. |
|  |  |
| Domain | Primary care structures |
| Subdomain | Primary care information systems |
| Feature | Patient platforms |
| **Indicator/question title** | Use of mHealth in primary care (inf3q36) |
| Indicator/question definition or question | Are the following mobile health (mHealth) services used in primary care?  medication reminders (select one)  appointment reminders (select one)  patient monitoring (select one) |
| Numerator/denominator or answer choices | yes, the programme is established (the programme has been running for at least two years, and is expected to continue for at least another two years)  yes, the programme is a pilot (the programme is tested and evaluated in specific situations)  yes, the programme exists at an informal level (there is an early adoption in the country, but no formal processes or policies are available)  no  do not know |
| Unit of measurement | categorical |
| Rationale | mHealth facilitates patients’ engagement in their health care and allows for better coordination of care. mHealth offers the ability to actively engage individuals in health care in ways that previously have not been possible [113]. |
| Preferred data sources | - WHO global survey on eHealth [39] - key informant |
| Disaggregation | none specified |
| Limitations | The indicator does not provide information on whether patients use these platforms. |

| Domain | Primary care structures |
| --- | --- |
| Subdomain | Primary care medicines |
| Feature | Availability of medicines |
| **Indicator/question title** | Reimbursement eligibility scheme for outpatient medicines (med1q37) |
| Indicator/question definition or question | a. Which is the key scheme for eligibility for reimbursement coverage for pharmaceuticals? (select one) |
| Numerator/denominator or answer choices | - product-specific reimbursement - disease-specific - population-groups-specific - consumption-based - no information |
| Unit of measurement | categorical |
| Indicator/question definition or question | b. Are there any other supplementary schemes for eligibility for pharmaceutical reimbursement? (select all that apply) |
| Numerator/denominator or answer choices | - product-specific - disease-specific - population-groups-specific - consumption-based - no other scheme - no information |
| Unit of measurement | categorical |
| Rationale | Eligibility for reimbursement coverage contributes to the understanding of universal health coverage in general and accessibility of medicines, a Sustainable Development Goal. The supply and prescription of primary care medicines must reflect appropriate evidence-based standards. Limits and imperfections in the system of medicine supply and financing can disrupt access to quality medicines [114]. |
| Preferred data sources | - WHO survey of the Pharmaceutical Pricing and Reimbursement Information Network [50] |
| Disaggregation | none specified |
| Limitations | non specified |

| Domain | Primary care structures |
| --- | --- |
| Subdomain | Primary care medicines |
| Feature | Availability of medicines in primary care |
| **Indicator/question title** | Availability of essential medicines for primary care (med1q156) |
| Indicator/question definition or question | Proportion of health facilities that have a core set of relevant essential medicines available on a sustainable basis |
| Numerator/Denominator or answer choices | As calculated for the reporting on SDG 3.b.3 indicator which captures not only the availability but also the affordability of a basket of essential medicines.  For detailed computation method and methodology please refer to the metadata of indicator SDG 3.b.3 [115]. |
| Unit of measurement | percent |
| Rationale | This is indicator is part of the SDG 3.b.3 which evaluates the access to medicines at health facilities and a detailed rational can be found in its metadata [115]. Access to medicines is an integral part of the universal health coverage movement and indispensable to the delivery of quality health care. Measuring and monitoring access to medicines is integral to understanding whether essential medicines are available and affordable. While the accessibility indicator combines both dimensions, availability and affordability, into a single evaluation, understanding only whether the basket of medicines is available at the facility level is important in evaluating the gaps in delivery of services. |
| Preferred data sources | as reported to the SDG monitoring (data collection through Health Action International Project supported by the WHO, The Service Availability and Readiness Assessment survey or the WHO Medicines Price and Availability Monitoring mobile application) |
| Disaggregation | as reported to the SDG; the calculation proposed for the SDG 3.b.3 allows for the following disaggregation:   - public/private facilities - geography – rural/urban areas - therapeutic group - facility type (pharmacy/hospital) - medicine |
| Limitations | The calculation for availability alone may not be readily available as the SDG 3.b.3 indicator combines availability and affordability. The 28 medicines identified for the SDG indicator cover tracers conditions relevant to the PHC-IMPACT (non-communicable diseases, mental health conditions, palliative care and anti-infective) as well as mother and child health, and antiretroviral, therefore a disaggregation by therapeutic group, if available, should be reported. For further limitations to this indicator please refer to the metadata of SDG 3.b.3 [115] |

| Domain | Primary care structures |
| --- | --- |
| Subdomain | Primary care diagnostics |
| Feature | Laboratory |
| **Indicator/question title** | **Availability of laboratory tests in primary care (dgn1q38)** |
| Indicator/question definition or question | Is laboratory medical equipment available in primary care facilities to carry out the following tests?   - blood glucose measurement - oral glucose tolerance test - HbA1c, diabetes testing - urine test glucose/sugar - urine test ketone bodies - total cholesterol measurement - urine strips for albumin assay - fecal occult blood test - PAP smear (cervical cytology) - HPV test - rapid tuberculosis diagnosis using WHO recommended rapid test such as Xpert MTB/RIF - rapid streptococcal test for throat swap |
| Numerator/denominator or answer choices | **Numerator:** number of facilities in the denominator that have available and functional the medical equipment on-site or the specimen can be collected at the facility and sent out by the staff  **Denominator:** number of primary care facilities surveyed  Alternate answer choices if exact data is not available:  generally available (in 50% or more facilities)  generally not available (in less than 50% of facilities)  not available |
| Unit of measurement | category |
| Rationale | The availability of timely diagnostic testing following screening and prevention services, as well as appropriate treatment as needed, have been recognized to contribute to the comprehensive delivery of services in primary care [116]. New technologies and testing processes can help to identify those in need of treatment early in the disease process [117]. A wide array of laboratory tests is utilized for the management of noncommunicable diseases. Selecting the appropriate mix of the most cost-effective technological applications is particularly challenging when investment is inadequate [96], [118]. For tuberculosis, this indicator is in line with the objective of increasing access to rapid and accurate WHO recommended rapid tests, and monitors whether countries aim to phase out microscopy as an initial diagnostic test which should be done by no later than 2025. Countries should not invest in establishing additional microscopy facilities. Countries that have positioned a WHO recommended rapid test as the initial diagnostic test for all people with signs and symptoms of tuberculosis and that have established reliable WHO recommended rapid tests supply systems and specimen referral systems, may create referral hubs for microscopy for treatment monitoring [119]. |
| Preferred data sources | - WHO Global Country Capacity and Response Survey on Noncommunicable Diseases Survey 2017 - health facility database - expert consensus |
| Disaggregation | public/private |
| Limitations | none specified |
|  |  |
| Domain | Primary care structures |
| Subdomain | Primary care diagnostics |
| Feature | Imaging |
| **Indicator/question title** | **Availability of diagnostic imaging in primary care (dgn2q39)** |
| Indicator/question definition or question | Is medical equipment available in primary care facilities to carry out the following diagnostic imaging?   - x-ray - electrocardiography - regular ultrasound - Doppler ultrasound (for foot vascular status) - sigmoidoscopy |
| Numerator/denominator or answer choices | **Numerator:** number of facilities in the denominator that have available and functional all the medical equipment on-site  **Denominator:** number of primary care facilities surveyed  Alternate answer choices if exact data is not available:  generally available (in 50% or more facilities)  generally not available (in less than 50% of facilities)  not available |
| Unit of measurement | category |
| Rationale | New technologies and testing processes can help to identify those in need of treatment early in the disease process and facilitate self-management [117]. The availability of timely diagnostic testing following screening and prevention services, as well as appropriate treatment as needed, have been recognized to contribute to the comprehensive delivery of services in primary care [116]. |
| Preferred data sources | - WHO Global Country Capacity and Response Survey on Noncommunicable Diseases Survey 2017 - health facility database - expert consensus |
| Disaggregation | public/private |
| Limitations | Availability of laboratory equipment/technology does not indicate that the services are necessarily being offered in primary care. The data source for these structures question is the WHO country capacity survey which does not distinguish between availability of technology, and offer of services [120]. |
|  |  |
| Domain | Primary care structures |
| Subdomain | Primary care technologies |
| Feature | Basic technology |
| **Indicator/question title** | **Availability of equipment in primary care (tch1q40)** |
| Indicator/question definition or question | Are the following medical devices/equipment available in primary care facilities?   - bag valve mask for manual resuscitation (e.g. Ambu bag) - blood pressure instruments - defibrillator - height scale - ophthalmoscope - peak flow meter/spirometer - tuning fork - weighing machine |
| Numerator/denominator or answer choices | **Numerator:** number of facilities in the denominator that have available and functional all the medical devices/equipment on-site  **Denominator:** number of primary care facilities surveyed  Alternate answer choices if exact data is not available:   - generally available (in 50% or more facilities) - generally not available (in less than 50% of facilities) - not available |
| Unit of measurement | exact percent if available, otherwise categorical |
| Rationale | To effectively provide essential health services, facilities must have a minimum level of essential technologies available. Inadequate equipment and supplies are one of the impediments to the delivery of primary care services [97]. The list of medical devices by health care facility type is available from WHO [121]. In addition, in the Package of Essential Noncommunicable Diseases Interventions for Primary Health Care a minimum level of essential technologies were identified to effectively provide essential health services [96]. The indicator/question draws from the Noncommunicable Diseases Global Monitoring Framework [122]. |
| Preferred data sources | - WHO Global Country Capacity and Response Survey on Noncommunicable Diseases Survey 2017 - health facility database - expert consensus |
| Disaggregation | public/private |
| Limitations | The availability of laboratory equipment/technology does not indicate that the services are necessarily being offered in primary care. The data source for these structures question is the WHO country capacity survey which does not distinguish between availability of technology, and offer of services [120]. |

| Domain | Primary care structures |
| --- | --- |
| Subdomain | Primary care facility infrastructure |
| Feature | Amenities |
| **Indicator/question title** | General service readiness at facility level (str1q42) |
| Indicator/question definition or question | a. Is facility improvement planned by the following levels of government?  central government (select one)  regional/oblast government (select one)  local government (municipal/district) (select one)  communities (select one) |
| Numerator/denominator or answer choices | - yes - no - do not know |
| Unit of measurement | categorical |
| Indicator/question definition or question | b. If the facility improvement plan exists, does it include the following considerations?   - accessibility for persons with disability (select one) - IT infrastructure (select one) |
| Numerator/denominator or answer choices | - yes - no - not applicable - do not know |
| Unit of measurement | categorical |
| Rationale | An accessible environment is necessary for an effective and functional health services delivery system and a key predictor of accessibility [123]. |
| Preferred data sources | - key informant |
| Disaggregation | none specified |
| Limitations | none specified |

| Domain | Model of primary care |
| --- | --- |
| Subdomain | Primary care selection of services |
| Feature | Identifying needs |
| **Indicator/question title** | **Population stratification (sel1q43)** |
| Indicator/question definition or question | Is the selection of services informed by population stratification? (select one) |
| Numerator/denominator or answer choices | - yes, by population risk - yes, by vulnerable status - yes, by both - no - do not know |
| Unit of measurement | categorical |
| Rationale | The assessment of health needs for a given population, stratifying for epidemiological, demographic or geographic variables is acknowledged as a precursor for the planning and targeting of services to manage needs and to proactively address known risk factors [7]. This focus on population health ensures, among other planning considerations such as financial resources, staff, medicines and supplies that the package of services is tailored to a defined population. |
| Preferred data sources | - key informant |
| Disaggregation | none specified |
| Limitations | none specified |

| Domain | Model of primary care |
| --- | --- |
| Subdomain | Primary care selection of services |
| Feature | Preventive care |
| **Indicator/question title** | Counselling services (sel1q44) |
| Indicator/question definition or question | If the following counselling services are provided in primary care please select those health professional that provide these services. Please answer according to regulation. If no regulation is in place, please specify in general.  tobacco (select all that apply)  physical activity (select all that apply)  intake of salt (select all that apply)  consumption of fruits and vegetables (select all that apply)  use of alcohol (select all that apply)  bodyweight (select all that apply)  family planning services (select all that apply)  psychological counselling for mental disorders (select all that apply) |
| Numerator/denominator or answer choices | generalist medical practitioner  nurse/midwife/feldscher/paramedical practitioner  narrow specialist  specialist  other working in primary care (specify)  public health professional (specify)  not provided in primary care (exclusive choice)  do not know (exclusive choice) |
| Unit of measurement | categorical |
| Rationale | A minimum set of interventions can be delivered by generalist medical practitioners, narrow specialists (in countries of the Commonwealth of Independent States), and non-physician health professionals in primary care. If effectively integrated into primary care they can make a significant contribution to the reduction of morbidity and premature mortality from major noncommunicable diseases. In general, the provision of a wide range of services provided in primary care is associated with better health outcomes at lower costs [95], [122], [96]. |
| Preferred data sources | - key informant |
| Disaggregation | none specified |
| Limitations | none specified |

| Domain | Model of primary care |
| --- | --- |
| Subdomain | Primary care selection of services |
| Feature | Preventive care |
| **Indicator/question title** | **Population based screenings (sel1q45)** |
| Indicator/question definition or question | a. How are the following screening programmes delivered?   - cervical cancer screening (select one) - breast cancer screening (select one) - colon cancer screening (select one) |
| Numerator/denominator or answer choices | - integrated into primary care - in primary care but organized as a vertical programme - as a vertical programme - other (please specify) - does not exist - do not know   comments and clarifications |
| Unit of measurement | categorical |
| Indicator/question definition or question | b. Is there dispensarization in primary care for the following conditions?   - cardiovascular disease (select one) - diabetes type 2 (select one) - respiratory disease (select one) - cancer (select one) - tuberculosis (select one) - mental health (select one)   Note: skip if not country of the Commonwealth of Independent States. |
| Numerator/denominator or answer choices | - yes - no - do not know |
| Unit of measurement | categorical |
| Rationale | Core individual services for early detection of priority diseases ensure people-centered primary health care. Priority interventions reflect those cost-effective services corresponding to effective approaches to reduce burden of noncommunicable diseases as identified in the Package of Essential Noncommunicable [61] Disease Interventions for Primary Health Care [96], [124], [125]. |
| Preferred data sources | - key informant |
| Disaggregation | none specified |
| Limitations | none specified |

| Domain | Model of primary care |
| --- | --- |
| Subdomain | Primary care selection of services |
| Feature | Preventive care |
| **Indicator/question title** | Individual risk assessments/stratification (sel2q46) |
| Indicator/question definition or question | If the following services are provided in primary care select those health professionals that provide these services. Please answer according to regulation. If no regulation is in place, please specify in general.  annual physical exam/health evaluation (select all that apply)  cardiovascular disease risk assessment (using WHO/ISH risk charts) (select all that apply)  cardiovascular disease risk stratification for the management of individuals at high risk for heart attack and stroke (select all that apply)  detection of hypertension using a risk prediction chart (select all that apply)  detection of diabetes type 2 using total risk approach (select all that apply)  tuberculosis symptoms detection for at risk populations (select all that apply)  mental health risk assessment (select all that apply)  HEADS assessment for adolescents (select all that apply) |
| Numerator/denominator or answer choices | generalist medical practitioner  nurse / midwife / feldscher / paramedical practitioner  narrow specialist  specialist  other working in primary care (specify)  not provided in primary care (exclusive choice)  not provided in the country (exclusive choice)  do not know (exclusive choice) |
| Unit of measurement | categorical |
| Rationale | A minimum set of preventive interventions can be delivered by generalist medical practitioners, narrow specialists (in countries of the Commonwealth of Independent States) and non-physician health workers in primary care. If effectively integrated into primary care, these preventive services can make a significant contribution to the reduction of morbidity and premature mortality from major noncommunicable diseases. First contact care by primary care health professionals is essential to address the wide variety and often very basic needs existing in the community [95], [125]. |
| Preferred data sources | - key informant |
| Disaggregation | none specified |
| Limitations | none specified |

| Domain | Model of primary care |
| --- | --- |
| Subdomain | Primary care selection of services |
| Feature | Preventive care |
| **Indicator/question title** | **Vaccination services (sel2q47)** |
| Indicator/question definition or question | Are the following vaccination services available in primary care?   - HPV vaccination for girls (select one) - HPV vaccination for boys (select one) - influenza vaccination for at risk population (elderly, pregnant women etc.) (select one) |
| Numerator/denominator or answer choices | - yes - no - do not know |
| Unit of measurement | categorical |
| Rationale | The HPV vaccination indicator is a core measure of the global monitoring framework for noncommunicable diseases which will track the implementation of the noncommunicable diseases action plan through monitoring and reporting on the attainment of the global targets in 2015-2020. The 25 indicators and the 9 voluntary global targets of the framework provide overall direction and the action plan provides a roadmap for reaching the targets [122]. Vaccination services are a core component of health promotion and disease prevention – key to the delivery of a broad range of services across stages of the lifespan in primary health care. |
| Preferred data sources | - WHO Global Country Capacity and Response Survey on Noncommunicable Diseases Survey - Seasonal influenza vaccination in Europe technical report [126] - key informant |
| Disaggregation | none specified |
| Limitations | none specified |

| Domain | Model of primary care |
| --- | --- |
| Subdomain | Primary care selection of services |
| Feature | Diagnostic procedures |
| **Indicator/question title** | Diagnostic exams (sel3q48) |
| Indicator/question definition or question | If the following exams are provided in primary care please select those health professionals that provide these services. Please answer according to regulation. If no regulation is in place, please specify in general.  dilated fundus examination (select all that apply)  Doppler ultrasound for foot vascular status (select all that apply)  electrocardiography (select all that apply)  peak flow measurement (select all that apply)  pulse oximetry (select all that apply)  regular ultrasound (select all that apply)  sigmoidoscopy (select all that apply)  spirometry (select all that apply)  x-ray (select all that apply)  Note: the indicator seeks information on the availability of each diagnostic exam in primary care. An evaluation of medical equipment necessary for these tests was sought in Primary Care Structures. |
| Numerator/denominator or answer choices | generalist medical practitioner  nurse/midwife/feldscher/paramedical practitioner  narrow specialist  specialist  other working in primary care (specify)  not provided in primary care (exclusive choice)  do not know (exclusive choice) |
| Unit of measurement | categorical |
| Rationale | The delivery of a wide range of interventions in primary care is associated to better health outcomes. When effectively integrated into primary care these services can significantly contribute to the reduction of morbidity and premature mortality from major noncommunicable diseases at lower costs. [95], [124], [118]. |
| Preferred data sources | - key informant |
| Disaggregation | none specified |
| Limitations | Data from WHO Country Capacity Survey is used to inform questions under the domain of primary care structures. To answer this indicator, which seeks to understand the interventions integrated into primary care, a key informant must be approached. |

| Domain | Model of primary care |
| --- | --- |
| Subdomain | Primary care selection of services |
| Feature | Diagnostic procedures |
| **Indicator/question title** | Final diagnosis in primary care (sel3q49) |
| Indicator/question definition or question | Which primary care health professionals can make the final diagnosis in primary care for the following conditions? Please answer according to regulation. If no regulation is in place, please specify in general.  hypertension (select all that apply)  ischemic heart disease (select all that apply)  diabetes type 2 (select all that apply)  asthma (select all that apply)  chronic obstructive pulmonary disease (select all that apply)  tuberculosis (select all that apply)  latent tuberculosis infection (select all that apply)  depression (select all that apply) |
| Numerator/denominator or answer choices | generalist medical practitioner  nurse / midwife / feldscher / paramedical practitioner  narrow specialist  specialist  other working in primary care (specify)  not provided in primary care (exclusive choice)  do not know (exclusive choice) |
| Unit of measurement | categorical |
| Rationale | Hierarchical processes in services delivery can perpetuate specialist-driven processes to diagnose and treat conditions that could be managed in primary care [127]. The International Classification of Primary Care recognizes the above reasons for patient encounters as problems/diagnosis that can be managed in primary care [128]. |
| Preferred data sources | - key informant |
| Disaggregation | none specified |
| Limitations | none specified |

| Domain | Model of primary care |
| --- | --- |
| Subdomain | Primary care selection of services |
| Feature | Treatment |
| **Indicator/question title** | **Prescribing authority of generalist medical practitioner (sel4q50)** |
| Indicator/question definition or question | Can generalist medical practitioners prescribe/refill the following medicine?  statin as secondary prevention for those individuals with prior CVD (heart attacks, strokes, and peripheral vascular disease) (select one)  statin as secondary prevention for individuals, 40+ years, registered for treatment with diabetes type 2 (select one)  penicillin as secondary prophylaxis for rheumatic fever and rheumatic heart disease (select one)  aspirin as secondary prevention for individuals diagnose with ischemic heart disease (select one)  angiotensin-converting enzyme inhibitor (ACE-I) (select one)  beta-blocker (select one)  calcium channel blockers (CCB) (ex. amlodipine) (select one)  thiazide or thiazide-like diuretic (select one)  metformin (select one)  insulin (select one)  sulphonylurea (e.g. glibenclamide) (select one)  bronchodilators (e.g. oral short-acting b2 agonists, inhaled short-acting b2 agonists) (select one)  inhaled steroids (select one)  nicotine replacement therapy (select one)  oral morphine (select one)  treatment for drug-susceptible tuberculosis: isoniazid, rifampicin, pyrazinamide, ethambutol (first line treatment: 2HRZE/4HR) (select one)  antipsychotics for psychotic disorders (chlorpromazine, fluphenazine, haloperidol, risperidone) (select one)  antidepressants for depression and anxiety disorders (amitriptyline, fluoxetine) (select one)  anxiolytics and tranquilizers for anxiety disorders and sleep disorders (diazepam) (select one)  anticonvulsant medicine and mood stabilizers for bipolar disorder (carbamazepine, lithium carbonate, valporic acid) (select one) |
| Numerator/denominator or answer choices | can prescribe/refill without recommendation from specialist medical practitioner/narrow specialist  can prescribe only with recommendation from specialist medical practitioner/narrow specialist, but can refill without recommendation  can prescribe/refill only with recommendation from specialist medical practitioner/narrow specialist  cannot prescribe but can refill without recommendation from specialist medical practitioner/narrow specialist  cannot prescribe but can refill with recommendation from specialist medical practitioner/narrow specialist  cannot prescribe/refill  not applicable  do not know |
| Unit of measurement | categorical |
| Rationale | This indicator measures the potential for essential drugs to be accessed through primary care that in turn can improve patient treatment adherence. Prescribing restrictions for essential medicines can have unintended effects [129]. While prescribing restrictions can contribute to improved quality of health services through effective and safe use of pharmaceuticals and improve cost-effectiveness of health services through the economic and efficient use of pharmaceuticals, it can also negatively affect the accessibility of medicine to the population. Improving access to quality medicines for noncommunicable diseases is one of the 15 health system challenges and opportunities to scale up core noncommunicable diseases interventions and services [130]. The cardiovascular and diabetes drugs in this list are core drugs listed in the HEARTS technical package [131]. Effective secondary prevention in primary health care is recognized as a core component in strengthening health systems responding to noncommunicable diseases [114]. Tuberculosis treatment should be in accordance with the guidelines for treatment of drug-susceptible tuberculosis and patient care [132] and fall in line with the Tuberculosis Regional Eastern European and Central Asian Project [133]. The authorized maximum duration of one prescription of strong opioids is an indication of access to morphine and development of primary care in a country. |
| Preferred data sources | Health Systems in Transition series  policy and programme documents  key informant |
| Disaggregation | none specified |
| Limitations | Tuberculosis guidelines are country specific. For drug susceptible tuberculosis some initial specialist medical practitioner’s involvement in prescribing drugs may be needed, for drug resistant tuberculosis, and particularly multi- and extensively-drug resistant tuberculosis, this is common in most countries. |

| Domain | Model of primary care |
| --- | --- |
| Subdomain | Primary care selection of services |
| Feature | Management of diseases |
| **Indicator/question title** | Follow-up services in primary care (sel5q51) |
| Indicator/question definition or question | If the below conditions are well controlled, who manages the patient in primary care? Please answer according to regulation. If no regulation is in place, please specify in general.  hypertension (select all that apply)  ischemic heart disease (select all that apply)  diabetes type 2 (select all that apply)  asthma (select all that apply)  chronic obstructive pulmonary disease (select all that apply)  cancer – breast (select all that apply)  cancer – cervical (select all that apply)  cancer – colorectal (select all that apply)  tuberculosis and latent tuberculosis infection (treatment management) (select all that apply)  depression (select all that apply) |
| Numerator/denominator or answer choices | - generalist medical practitioner - nurse/midwife/feldscher/paramedical practitioner - narrow specialist - specialist - other working in primary care (specify) - not provided in primary care (exclusive choice)   do not know (exclusive choice) |
| Unit of measurement | categorical |
| Rationale | Improving the coordination of services is central to delivering quality, integrated health services. The coordination of care is not only about the coordination across service providers, but also about coordinating care over time, through improved information flows and maintaining relationships with providers. Primary care driven follow-up offers a gateway to coordinated service provision and the delivery of services that are provided in close communication between generalist and specialist providers [117]. |
| Preferred data sources | - key informant |
| Disaggregation | none specified |
| Limitations | none specified |
|  |  |
| Domain | Model of primary care |
| Subdomain | Primary care selection of services |
| Feature | Management of diseases |
| **Indicator/question title** | Other services (sel5q52) |
| Indicator/question definition or question | Who provides the following services in primary care? Please answer according to regulation. If no regulation is in place, please specify in general.   - administration of intravenous fluids/drips (select all that apply) - administration of oxygen (mask or tube) (select all that apply) - cardiopulmonary resuscitation (select all that apply) - foot vibration perception by tuning fork (select all that apply) - intramuscular/subcutaneous injection (select all that apply) - intravenous injection (select all that apply) - manual ventilation with a bag valve mask resuscitator (ambu-bag) (select all that apply) - ophthalmoscopy (select all that apply) - post-natal care check of mother (select all that apply)   visual acuity examination (select all that apply)  visual inspection and examination of diabetic individuals’ feet for the detection of risk factors for ulceration (select all that apply) |
| Numerator/denominator or answer choices | - generalist medical practitioner - nurse/midwife/feldscher/paramedical practitioner - narrow specialist - specialist - other working in primary care (specify) - not provided in primary care (exclusive choice) - do not know (exclusive choice) |
| Unit of measurement | categorical |
| Rationale | A minimum set of interventions can be delivered by generalist medical practitioners, narrow specialists (in countries of the Commonwealth of Independent States) and non-physician primary care health professionals. If effectively integrated into primary care they can make a significant contribution to the reduction of morbidity and premature mortality from major noncommunicable diseases. Preventive health services are cost-effective in the primary care setting and result in improved levels of population health. In general, the provision of a wide range of services provided by primary care providers is associated with better health outcomes at lower costs [95],[96],[124], [118]. |
| Preferred data sources | - key informant |
| Disaggregation | none specified |
| Limitations | Data from WHO Country Capacity Survey is used to inform questions under the domain of primary care structures. To answer this indicator, which seeks to understand the interventions integrated into primary care, a key informant must be approached. |

| Domain | Model of primary care |
| --- | --- |
| Subdomain | Primary care selection of services |
| Feature | Patient engagement |
| **Indicator/question title** | Self-management and health literacy in primary care (sel6q54) |
| Indicator/question definition or question | To enhance patient self-management and health literacy, do the following exist in primary care?  telephone-based services (select one)  computer-based programmes (e.g. internet-based chat rooms, virtual support group) (select one)  printed resources (e.g. pictograms, pamphlets, brochures, etc.) (select one)  in-home electronic aids (e.g. blood pressure cuff, blood glucose device etc.) (select one)  one-on-one patient education (e.g. nurse and patient) (select one)  patients school (select one)  peer support groups (select one) |
| Numerator/denominator or answer choices | yes, country-wide  yes, in some regions (please specify)  yes, is only being piloted  no  do not know |
| Unit of measurement | categorical |
| Rationale | Strengthening health literacy enables people to make important health services decisions and to communicate, assert and enact these decisions [134]. Strengthened health literacy improves health outcomes, the effective use of health services and reduces health inequities [135]. Low levels of health literacy are associated with unhealthy choices and lifestyle and riskier behaviours [136]. Self-management has been associated with improved health outcomes, reductions in service use, improved treatment adherence, increased access and convenience for patients, reduced hospitalizations, reduced emergency visits, fewer preventable hospitalizations, high patient and physician satisfaction and fewer unmet needs for getting around. An important part of patient education is increasing their awareness about the importance of disease prevention and health promotion as patients with certain co-morbidities are at increased risk for other related conditions [137], [138]. Services that work to link patients with peers can increase access to expert advice about how to manage both clinical and social aspects of a condition. It can also help to overcome feelings of isolation [117]. |
| Preferred data sources | - key informant |
| Disaggregation | none specified |
| Limitations | none specified |
|  |  |
| Domain | Model of primary care |
| Subdomain | Primary care design |
| Feature | Referral system |
| **Indicator/question title** | Gatekeeping system (des1q55) |
| Indicator/question definition or question | a. Do generalist medical practitioners act as a gatekeeper to services offered by specialist medical practitioners and other health professionals? (select one) |
| Numerator/denominator or answer choices | yes, a generalist medical practitioner’s referral is compulsory to access most types of specialist care (except in case of emergency)  no, but individuals have financial incentives to obtain a generalist medical practitioner’s referral (e.g. reduced co-payments), but direct access is always possible  no, there is no need and no incentive to obtain the generalist medical practitioner’s referral  do not know |
| Unit of measurement | categorical |
| Indicator/question definition or question | b. If yes, please specify for which type of specialist medical practitioner/narrow specialist (if any) referral is not compulsory. |
| Numerator/denominator or answer choices | open answer |
| Unit of measurement | open answer |
| Rationale | Gatekeeping systems have multiple positive effects on health services delivery. Most importantly, gatekeeping has been associated with cost containment, increased responsiveness to patients’ needs and enhanced quality of care [95]. First contact care by primary care providers is essential to address the wide variety and often very basic needs existing in the community. Having a generalist medical practitioner rather than a specialist medical practitioner as a regular source of care has been associated with better health outcomes and lower health care costs. |
| Preferred data sources | - OECD Health Systems Characteristics Survey - policy and programme documents - Health Systems in Transitions series - key informant |
| Disaggregation | - rural/urban |
| Limitations | none specified |
|  |  |
| Domain | Model of primary care |
| Subdomain | Primary care design |
| Feature | Referral system |
| **Indicator/question title** | Referral protocol from primary care to higher levels of care (des1q56) |
| Indicator/question definition or question | a. Is there a structured referral letter required when a generalist medical practitioner refers an individual to a higher level of care? (select one) |
| Numerator/denominator or answer choices | yes, country-wide  yes, in some regions (please specify)  yes, is only being piloted  no  do not know |
| Unit of measurement | categorical |
| Indicator/question definition or question | b. If a structured referral letter is required, is the following information included?   - individual’s identification information (select one) - reason for referral (e.g. investigation, diagnosis, treatment, reassurance etc.) (select one) - information related to illness (e.g. history, findings etc.) (select one) - information related to relevant investigations already undertaken (select one) - medication list (select one) - socio-psychological factors (select one) - generalist practitioner’s contact details (select one) |
| Numerator/denominator or answer choices | yes  no  do not know |
| Unit of measurement | categorical |
| Rationale | The delivery of coordinated health services depends on the accessibility and exchange of information among those involved in the care of an individual. The use of referral letters can facilitate this [95]. Information regarding the content of the referral letter is important in assessing the quality of a referral, which impacts the quality of care. Good communication can avoid problems related to polypharmacy, duplication of investigations, etc. |
| Preferred data sources | - review of national health policies - WHO Global Country Capacity and Response Survey on Noncommunicable Diseases Survey 2017 - key informant |
| Disaggregation | none specified |
| Limitations | none specified |
|  |  |
| Domain | Model of primary care |
| Subdomain | Primary care design |
| Feature | Referral system |
| **Indicator/question title** | Reply and discharge protocol from higher levels of care to primary care (des1q57) |
| Indicator/question definition or question | a. Is there a structured reply letter required when a specialist medical practitioner discharges an individual from their care to primary care? (select one) |
| Numerator/denominator or answer choices | yes, country-wide  yes, in some regions (please specify)  yes, is only being piloted  no  do not know |
| Unit of measurement | categorical |
| Indicator/question definition or question | b. If the structured reply letter is required, is the following information included?   - assessment of current problem (select one) - investigation undertaken (select one) - medication prescribed (select one) - next steps in the care of the individual (select one) |
| Numerator/denominator or answer choices | yes  no  do not know |
| Indicator/question definition or question | c. Is there a structured discharge letter required when the hospital discharges an individual from their care to primary care? (select one) |
| Numerator/denominator or answer choices | yes, country-wide  yes, in some regions (please specify)  yes, is only being piloted  no  do not know |
| Unit of measurement | - categorical |
| Indicator/question definition or question | d. If a discharge letter is required, is the following information included?   - assessment of current problem (select one) - investigation undertaken (select one) - medication prescribed (select one) - next steps in the care of the individual (select one) |
| Numerator/denominator or answer choices | - yes - no   do not know |
| Unit of measurement | categorical |
| Indicator/question definition or question | e. Is discharge planning required upon discharge from hospital? |
| Numerator/denominator or answer choices | - yes, country-wide - yes, in some regions (please specify) - yes, is only being piloted - no - do not know |
| Unit of measurement | categorical |
| Indicator/question definition or question | f. Based on need, is there an integrated health and social care plan required upon discharge from hospital? |
| Numerator/denominator or answer choices | - yes, country-wide - yes, in some regions (please specify) - yes, is only being piloted - no - do not know |
| Unit of measurement | categorical |
| Rationale | The delivery of coordinated health services depends on the accessibility and exchange of information among those involved in the care of an individual. The use of referral letters can facilitate this [95]. A health and social care plan (in addition to single point of access, and a care coordinator) are important to improve the rehabilitation, re-enablement and recovery experience for the individual and their carers. Its existence is associated with improved health outcomes and care experiences and thus lower re-hospitalization rates [139], [140]. |
| Preferred data sources | - review of national health policies - key informant |
| Disaggregation | none specified |
| Limitations | none specified |
|  |  |
| Domain | Model of primary care |
| Subdomain | Primary care design |
| Feature | Care pathways |
| **Indicator/question title** | Shared care pathways (des2q58) |
| Indicator/question definition or question | For the following conditions, are care pathways spanning different levels of care defined?   - cardiovascular diseases (select one) - diabetes type 2 (select one) - cancer – breast (select one) - cancer – cervical (select one) - cancer – colorectal (select one) - asthma (select one) - chronic obstructive pulmonary disease (select one) - tuberculosis (select one) - latent tuberculosis infection (select one) - depression (select one) |
| Numerator/denominator or answer choices | - yes, national care pathways guidelines - yes, regional care pathways guidelines - yes, included in national clinical practice protocols - no guidelines exist - other, please specify - do not know |
| Unit of measurement | - categorical |
| Rationale | Clearly designed care has also been found to contribute to improvements in services provision including minimizing discrepancies in core services in terms of both what is provided and how care is delivered. Care pathways have also been found to support the delivery of relevant services in a timely manner, to reduce complications and to enable better discharge planning [117]. |
| Preferred data sources | - Health Systems in Transition series - review of national health policies - key informant |
| Disaggregation | none specified |
| Limitations | none specified |

| Domain | Model of primary care |
| --- | --- |
| Subdomain | Primary care design |
| Feature | Flexible access modes |
| **Indicator/question title** | 59. Different access modes (des3q59) |
| Indicator/question definition or question | Percent of primary care providers that offer the following modes of care  individuals can telephone their regular primary care provider or support staff for questions or a consultation  individuals can email their regular primary care provider or support staff for questions or a consultation  make home visits  a member of the primary care team contacts individuals with multiple chronic conditions or complex needs between visits to monitor their condition |
| Numerator/denominator or answer choices | Exact percent reported in survey analysis  Alternate answer choices if exact data is not available:  70% or more  more than 50% but less than 70%  10% to 50%  less than 10% |
| Unit of measurement | exact percent if available, otherwise categorical |
| Rationale | The accessibility of primary care for persons with multiple chronic conditions can be improved by providing multiple access modes. This has been associated with reductions in demands for home health care and nursing facility admissions, improved quality of care, reduced family caregiver strain, increased physician satisfaction with care provided, reduced unnecessary emergency visits, hospitalisation and admissions, reduced hospital costs and improved quality of life [95], [138], [141], [142], [143], [144], [145], [146], [147], [148]. |
| Preferred data sources | Commonwealth Fund International Survey of Primary Care Physicians in 10 Nations [36]  survey – health professionals  expert consensus |
| Disaggregation | none specified |
| Limitations | none specified |
|  |  |
| Domain | Model of primary care |
| Subdomain | Primary care design |
| Feature | Shared care plans |
| **Indicator/question title** | **Developing shared care plans (des4q60)** |
| Indicator/question definition or question | Percent of primary care health professionals who engage with relevant specialists in the development of care plans for persons with multiple chronic conditions and receive care from more than one provider (select one) |
| Numerator/denominator or answer choices | Exact percent reported in survey analysis  Alternate answer choices if exact data is not available:  70% or more  more than 50% but less than 70%  10% to 50%  less than 10%  not applicable |
| Unit of measurement | exact percent, if available, or category |
| Rationale | Persons with multiple chronic conditions require care that is targeted around their individual needs, capabilities and resources. This should be planned and formalized in a care plan that is developed and shared with the patient and their (informal) caregivers as well as their regular care providers. Comprehensive and holistic assessments of needs, including the development of personalized care plans, have been associated with greater patient satisfaction, improved care coordination and reduced costs of care in older people and those with complex care needs [149]. |
| Preferred data sources | survey – health professionals  expert consensus |
| Disaggregation | none specified |
| Limitations | none specified |

| Domain | Model of primary care |
| --- | --- |
| Subdomain | Primary care workforce organization |
| Feature | Practice population |
| **Indicator/question title** | Choice of generalist medical practitioner (org1q61) |
| Indicator/question definition or question | a. Are individuals free to choose their primary care provider? (select one) |
| Numerator/denominator or answer choices | yes, the individual is free to choose the provider  yes, the individual is free to choose the provider, but the choice is limited (e.g. to a small geographical area, or to a specific network of providers)  yes, the individual is free to choose any provider, but have financial incentives (e.g. reduced co-payments) to choose certain ones  no, the individual is assigned to a specific provider (e.g. a health centre serving a geographical area)  do not know  comments or clarifications |
| Unit of measurement | categorical |
| Indicator/question definition or question | b. Are individuals free to choose their generalist medical practitioner within the chosen or assigned provider/practice? (select one) |
| Numerator/denominator or answer choices | - yes, the individual is free to choose the generalist medical practitioner within the chosen/assigned practice - no, the individual is assigned to a specific general medical practitioner within the chosen/assigned practice - not relevant (primary care services are predominantly provided by physicians in solo practice) - do not know   comments or clarifications |
| Unit of measurement | categorical |
| Rationale | The possibility to freely chose a primary care provider contributes to a positive relationship relative to an assigned practitioner. The evidence is strong regarding the benefits of a continuous relationship with a specific provider rather than with a specific place or no place at all [95]. |
| Preferred data sources | - OECD Health Committee Survey on Health Systems - Health Systems in Transition series - policy and programme documents - key informant |
| Disaggregation | none specified |
| Limitations | none specified |
|  |  |
| Domain | Model of primary care |
| Subdomain | Primary care workforce organization |
| Feature | Practice population |
| **Indicator/question title** | Patient list system (org1q62) |
| Indicator/question definition or question | Do generalist medical practitioners have a patient list? (select one) |
| Numerator/denominator or answer choices | yes  no  do not know |
| Unit of measurement | categorical |
| Rationale | Having a defined practice population by means of a patient list system creates an incentive for primary care providers as well as the population to provide and receive services on a continuous basis [95]. Registering with a specific practitioner has been found to contribute to accountability by making clear who is responsible for service coordination [117]. |
| Preferred data sources | - Health Systems in Transition series - policy and programme documents - key informant |
| Disaggregation | none specified |
| Limitations | none specified |

| Domain | Model of primary care |
| --- | --- |
| Subdomain | Primary care workforce organization |
| Feature | Practice population |
| **Indicator/question title** | **Primary care health professionals’ density (org1q63)** |
| Indicator/question definition or question | Number of generalist medical practitioners working in primary care per 100,000 population |
| Numerator/denominator or answer choices | **Numerator:** number of practising generalist medical practitioners (the number should be at the end of the calendar year) x 100,000  **Denominator:** resident population for the same calendar year |
| Unit of measurement | ratio |
| Rationale | Patient load can negatively influence the accessibility of providers and their job satisfaction as well as the experience of patient's with health services [95]. |
| Preferred data sources | - WHO European database on human and technical resources for health (numerator) [57] - population data from United Nations Population Division’s world population prospects database (denominator) [72] - Health Systems in Transition series - database – human resources |
| Disaggregation | rural-urban |
| Limitations | Data reported to the WHO European database on human and technical resources for health does not include paediatricians for countries of the Commonwealth of Independent States. 2013 is the latest year for which data is reported. |
|  |  |
| Domain | Model of primary care |
| Subdomain | Primary care workforce organization |
| Feature | Practice population |
| **Indicator/question title** | **Caseload of generalist medical practitioner (org2q64)** |
| Indicator/question definition or question | What is the average number of outpatient visits seen by a full-time generalist medical practitioner per day? |
| Numerator/denominator or answer choices | Database data:  **Numerator:** total number of outpatient visits conducted by a generalist medical practitioner (during 12-month reference period)  **Denominator:** total number of practising generalist practitioners (full time equivalent) (the number should be at the end of the calendar year) x number of working days in the year  Survey data:  Exact average number of outpatient visits per generalist medical practitioner per day from facility survey analysis |
| Unit of measurement | average number of visits per day |
| Rationale | Provider caseload can have critical impacts on service quality: a shortage of providers may cause caseload to rise and potentially compromise service quality and lead to provider burnout. Conversely, low caseloads may impact provider motivation, absenteeism and the practice of skills and procedures [97]. Low rates can also be indicative of poor availability and quality of services. For example, several countries have demonstrated that outpatient department rates go up when constraints to using such health services are removed, such as by bringing services closer to the people or reducing user fees. In contrast, once rates exceed an uncertain threshold, the number of visits is no longer an indicator of the strength of the health services [106]. |
| Preferred data sources | - health information system - survey – health facilities |
| Disaggregation | rural-urban |
| Limitations | Caseload does not measure the full workload experienced by a provider, which includes administrative work and other non-clinical activities. It also does not capture quality of care [97]. The accuracy and completeness of reporting need to be consistent over time and between populations to allow assessment of trends and comparisons [106]. |

| Domain | Model of primary care |
| --- | --- |
| Subdomain | Primary care workforce organization |
| Feature | After-hours care |
| **Indicator/question title** | Opening hours in primary care (org2q65) |
| Indicator/question definition or question | a. Do primary care providers have a required number of opening hours and days? (select one) |
| Numerator/denominator or answer choices | yes, obliged legally  yes, standard formulated by professional organisations  yes, decided by the employer  no  do not know |
| Unit of measurement | categorical |
| Indicator/question definition or question | b. If yes, how many hours or days? |
| Numerator/denominator or answer choices | hours/day, please specify  days/week, please specify  hours/week, please specify |
| Unit of measurement | hours |
| Rationale | A minimum number of opening hours or days ensures primary care services have a certain predictability for the population as well as physicians [95]. Opening hours is often used as a measure of the accessibility of services or health practitioners [116]. |
| Preferred data sources | - Health Systems in Transition series - policy and programme documents - key informant |
| Disaggregation | none specified |
| Limitations | none specified |
|  |  |
| Domain | Model of primary care |
| Subdomain | Primary care workforce organization |
| Feature | After-hours care |
| **Indicator/question title** | Out-of-hours primary care (org2q66) |
| Indicator/question definition or question | Are the following arrangements in place in primary care for individuals to see a generalist medical practitioner or nurse when the practices are closed without going to the hospital emergency room or department?   - generalist medical practitioners available in-person for their own patients (select one) - group of generalist medical practitioners available on a rota basis (select one) - primary care centres (mini injury units, urgent care centres) available (select one) - general practitioners’ cooperatives available (select one) - other arrangements, please specify (select one) |
| Numerator/denominator or answer choices | yes, country-wide  yes, in some regions (please specify)  yes, is only being piloted  no  do not know |
| Unit of measurement | N/A |
| Unit of measurement | N/A |
| Rationale | Primary care is well placed to assess acute episodes of chronic conditions to implement informed shared decision-making. The availability of 24/7 care with effective out-of-hours arrangements can help primary care to ensure effective triage to specialists. Systems without out-of-hours care can fuel unnecessary hospitalization and non-urgent visits [150]. |
| Preferred data sources | - OECD Survey on Health systems characteristics - key informant |
| Disaggregation | - rural-urban |
| Limitations | none specified |

| Domain | Model of primary care |
| --- | --- |
| Subdomain | Primary care workforce organization |
| Feature | Primary care teams |
| **Indicator/question title** | **Types of primary care facilities (org3q153)** |
| Indicator/question definition or question | a. If the following facilities provide ambulatory health care services, select the types of primary care health professionals working there. Please answer according to regulation. If no regulation is in place, please specify in general.  offices of single general medical practitioners – solo practices (e.g. general medical practitioner solo practice) (select all that apply)  offices of general medical practitioners - ambulatory group practices (e.g. walk-in offices/centres of multiple general medical practitioners) (select all that apply)  ambulatory multi-profile (specialty) group practices/polyclinics (select all that apply)  nurses and midwives offices (e.g. health posts) (select all that apply)  offices of other medical specialists (e.g. practices of independent offices of cardiologists, ophthalmologists, paediatricians of specialised care, etc.) (select all that apply)  other ambulatory health care centres (e.g. family planning centres, free-standing ambulatory surgery centres, dialysis care centres) (please specify) (select all that apply)  dental practices (select all that apply)  providers of home health care services (e.g. community nurses and domiciliary nursing care, home health care agencies, in-home hospice care services, etc.) (select all that apply) |
| Numerator/denominator or answer choices | - generalist medical practitioner - nurse/midwife/feldscher/paramedical practitioner - narrow specialist - specialist - other in primary care (please specify) - do not know |
| Unit of measurement | categorical |
| Indicator/question definition or question | b. Are primary health care / ambulatory services being delivered in the following settings? Please answer according to regulation. If there no regulation is in place, please specify in general.   - outpatient departments of hospitals (general hospitals providing out-patient, day care services) (select one) - residential long-term care facilities (e.g. long-term nursing care facilities) (select one) - providers of ancillary services (e.g. medical and diagnostic laboratories) (select one) - pharmacies, retailers and other providers of medical goods (e.g. pharmacies, suppliers of medical goods and medical appliances, patient transportation) (select one) - providers of preventive care (e.g. health promotion and protection agencies, public health institutes) (select one) |
| Numerator/denominator or answer choices | - yes - no - do not know |
| Unit of measurement | categorical |
| Rationale | Delivery settings describe the arrangement of providers in the various facilities, units or organizations where health services are delivered for a defined population. The way in which delivery settings are organized has been attributed to measures of performance including the accessibility of services [7]. |
| Preferred data sources | policy and programme documents  key informant |
| Disaggregation | none specified |
| Limitations | none specified |

| Domain | Model of primary care |
| --- | --- |
| Subdomain | Primary care workforce organization |
| Feature | Primary care teams |
| **Indicator/question title** | Shared practices in primary care (org3q67) |
| Indicator/question definition or question | Percent of primary care providers that are:  staffed only by a nurse/mid-wife/feldsher (no generalist medical practitioner)  one generalist medical practitioner (solo)  2 or 3 generalist medical practitioners in the same building without specialist medical practitioners  4 or more generalist medical practitioners in the same building without specialist medical practitioners  mixed practice with generalist medical practitioners and specialist medical practitioners |
| Numerator/Denominator or answer choices | **Numerator:** number of providers with the specified characteristic  **Denominator:** total number of providers |
| Unit of measurement | percent |
| Rationale | Group practices and teams with a greater occupational diversity are associated with a higher quality of care. Close involvement of generalist clinicians in specialty care leads to more cost-effective services and better outcomes [95]. The organization of health services supply potentially influences the accessibility to health services, their effectiveness, efficiency and quality, as well as provider and patient satisfaction. Generally, group practices are found to increase accessibility to care and professional working conditions, as well as the effectiveness and efficiency of health services delivery as several health professionals work together in collaboration [151]. |
| Preferred data sources | Health Systems in Transition series  policy and programme documents  registries of health professionals |
| Disaggregation | none specified |

| Domain | Model of primary care |
| --- | --- |
| Subdomain | Primary care workforce organization |
| Feature | Primary care teams |
| **Indicator/question title** | **Coordination within primary care (org3q68)** |
| Indicator/question definition or question | Percent of generalist medical practitioners that have regular meetings with the following professionals?  other generalist medical practitioners  nurse  social worker  psychologist  dietician  pharmacist  public health professional  Note: regular meetings include face-to-face, phone, or virtual discussions at least once per month |
| Numerator/denominator or answer choices | Exact percent reported in survey analysis  Alternate answer choices if exact data is not available:  70% or more  more than 50% but less than 70%  10% to 50%  less than 10% |
| Unit of measurement | exact percent if available, otherwise categorical |
| Rationale | Close collaboration between different primary care health professionals optimizes the treatment of individuals and therefore increases the strength of primary care. Regardless of the mode of teamwork that is applied there should be some form of structural communication among primary care health professionals treating the same individual [95]. |
| Preferred data sources | Health Systems in Transition series  policy and programme documents  survey – health professionals  expert consensus |
| Disaggregation | none specified |
| Limitations | none specified |
|  |  |
| Domain | Model of primary care |
| Subdomain | Primary care workforce organization |
| Feature | Primary care teams |
| **Indicator/question title** | **Existence of care coordinator (org3q70)** |
| Indicator/question definition or question | Percent of primary care providers that use a care coordinator (nurses or case managers) to monitor and manage care for individuals with chronic conditions that need regular follow-up care |
| Numerator/denominator or answer choices | Exact percent reported in survey analysis  Alternate answer choices if exact data is not available:  70% or more  more than 50% but less than 70%  10% to 50%  less than 10%  not applicable |
| Unit of measurement | exact percent if available, otherwise categorical |
| Rationale | Continuity in the relationship with health professionals is associated with improved communication and coordination of care, fewer emergency visits, hospitalisations and readmissions, reduced health care utilization, reduced hospital costs, better preventative care, fewer duplicative medications, improved patient outcomes and patient satisfaction, and more efficient use of resources [141], [142], [143], [152], [153]. Care coordinators or care managers can support the continuity of services through the management of patients and coordination of services across the continuum of care, overtime. |
| Preferred data sources | Commonwealth Fund International Survey of Primary Care Physicians in 10 Nations [36]  policy and programme documents  survey – health professionals  expert consensus |
| Disaggregation | none specified |
| Limitations | none specified |

| Domain | Model of primary care |
| --- | --- |
| Subdomain | Primary care workforce organization |
| Feature | Collaboration of primary care with other professionals |
| **Indicator/question title** | **Cooperation with specialist medical practitioners (org4q73)** |
| Indicator/question definition or question | a. Percent of generalist medical practitioners who engage in the following forms of cooperation with specialist medical practitioners  specialist medical practitioners visit a primary care practice to provide outpatient consultations/visits normally provided in hospital (replaced specialist care)  specialist medical practitioners visit a primary care practice to provide joint outpatient consultations/visits with generalist medical practitioners  generalist medical practitioners receive clinical lessons/training from specialist medical practitioners |
| Numerator/denominator or answer choices | Exact percent reported in survey analysis  Alternate answer choices if exact data is not available:  70% or more  more than 50% but less than 70%  10% to 50%  less than 10% |
| Unit of measurement | exact percent if available, otherwise categorical |
| Indicator/question definition or question | b. Percent of generalist medical practitioners who ask advice (e.g. e-mail, in-person, telephone, skype, etc.) from specialist medical practitioners (e.g. paediatricians, internists, gynaecologists, surgeons, cardiologists, pulmonologists, endocrinologists, etc.)? |
| Numerator/denominator or answer choices | Exact percent reported in survey analysis  Alternate answer choices if exact data is not available:  70% or more  more than 50% but less than 70%  10% to 50%  less than 10% |
| Unit of measurement | exact percent if available, otherwise categorical |
| Rationale | Shared care arrangements between primary and secondary care providers stimulates mutual education, promotes cooperation across levels, improves guideline-consistent care, reduces the use of inpatient services and improves appropriate prescribing and medication adherence and contributes to improved health outcomes [95]. |
| Preferred data sources | survey – health professionals  expert consensus |
| Disaggregation | none specified |
| Limitations | none specified |

| Domain | Model of primary care |
| --- | --- |
| Subdomain | Primary care workforce organization |
| Feature | Collaboration of primary care with other professionals |
| **Indicator/question title** | **Coordination across sectors (org4q69)** |
| Indicator/question definition or question | Percent of professionals from different sectors (incl. community health, mental health, social care, primary and hospital care) who are integrated in a care team with a shared governance model to care for individuals with multiple chronic conditions or complex needs |
| Numerator/denominator or answer choices | Exact percent reported in survey analysis  Alternate answer choices if exact data is not available:  70% or more  more than 50% but less than 70%  10% to 50%  less than 10% |
| Unit of measurement | exact percent if available, otherwise categorical |
| Rationale | Care teams can range from the basic unit of general medical practitioners and nurses, to larger, multi-sector teams that engage health and social care workers. Across-sector teams can allow for improved collaboration and knowledge exchange between providers working in different settings [117]. |
| Preferred data sources | Health Systems in Transition series  policy and programme documents  survey – health professionals  expert consensus |
| Disaggregation | none specified |
| Limitations | none specified |

| Domain | Model of primary care |
| --- | --- |
| Subdomain | Primary care services management |
| Feature | Primary care staffing |
| **Indicator/question title** | **Autonomy in staffing of medical staff (man1q74)** |
| Indicator/question definition or question | What is the level of autonomy for managing primary care facilities with respect to:   - recruitment and hiring of medical staff (select one) - remuneration level of medical staff (select one) |
| Numerator/denominator or answer choices | - complete autonomy - must negotiate with local authorities - central or subnational government decides - other arrangements, please specify in comments - do not know   comments or clarifications |
| Unit of measurement | categorical |
| Rationale | Autonomy of managers is a key predictor of the degree to which services and their arrangements are tailored to the community’s needs. A manager's autonomy to ensure that the right people are in the right jobs is critical to ensure resources are used optimally [116]. |
| Preferred data sources | - key informant |
| Disaggregation | none specified |
| Limitations | none specified |
|  |  |
| Domain | Model of primary care |
| Subdomain | Primary care services management |
| Feature | Managing primary care facilities |
| **Indicator/question title** | **Degree of autonomy in budgeting (man2q75)** |
| Indicator/question definition or question | a. Do primary care facilities have an autonomous budgeting process? (select one) |
| Numerator/denominator or answer choices | - yes, country-wide - yes, in some regions (please specify) - yes, is only being piloted - no - do not know   comments or clarifications |
| Unit of measurement | categorical |
| Indicator/question definition or question | b. If yes, do primary care managers use scenario planning? (select one) |
| Numerator/denominator or answer choices | - yes, country-wide - yes, in some regions (please specify) - yes, is only being piloted - no - do not know   comments or clarifications |
| Unit of measurement | categorical |
| Indicator/question definition or question | c. If yes (question a), do primary care managers have the autonomy to transfer funds between budget lines? (select one) |
| Numerator/denominator or answer choices | - yes, for the whole budget - yes, for a portion of the budget - no - do not know   comments or clarifications |
| Unit of measurement | categorical |
| Indicator/question definition or question | d. If yes (question a), do primary care managers have the autonomy to invest savings? (e.g. new services, invest in technology, bonuses, etc.) (select one) |
| Numerator/denominator or answer choices | - yes, country-wide - yes, in some regions (please specify) - yes, is only being piloted - no - do not know   comments or clarifications |
| Unit of measurement | categorical |
| Rationale | Managing services refers to the oversight of operations, to bring about order and consistency in their day-to-day delivery; the ability to do so being vital to cope with complexity and guide operations in the production process to secure optimal outcomes. Autonomy over resource management is linked to the allocation of resources and introduction of innovative resources. The investment of managers in primary care has been shown to contribute to the provision of health promotion and prevention services, improvements in planning and monitoring and the ability to identify high-risk individuals for more targeted care and in contributing to the reduction of inequities [116]. |
| Preferred data sources | - key informant |
| Disaggregation | none specified |
| Limitations | none specified |
|  |  |
| Domain | Model of primary care |
| Subdomain | Primary care services management |
| Feature | Managing primary care facilities |
| **Indicator/question title** | **Health care technology management (man2q76)** |
| Indicator/question definition or question | Is a maintenance programme for all available medical equipment organized at facility level in primary care? (select one) |
| Numerator/denominator or answer choices | - yes, country-wide - yes, in some regions (please specify) - yes, is only being piloted - no - do not know   comments or clarifications |
| Unit of measurement | categorical |
| Rationale | Planning a maintenance programme is part of a broader effort to establish a comprehensive programme for healthcare technology management. The planning process includes considerations of inventory, identifying the method by which maintenance will be provided to the items included in the programme, and allocating resources (financial, physical and human resources) to the programme [154]. |
| Preferred data sources | - survey – facilities - key informant |
| Disaggregation | none specified |
| Limitations | none specified |

| Domain | Model of primary care |
| --- | --- |
| Subdomain | Primary care services management |
| Feature | Strategic planning |
| **Indicator/question title** | **Population health management (man3q77)** |
| Indicator/question definition or question | a. Are health services planned at the facility level based on the needs of the catchment area? (select one) |
| Numerator/denominator or answer choices | - yes, country-wide - yes, in some regions (please specify) - yes, is only being piloted - no - do not know   comments or clarifications |
| Unit of measurement | categorical |
| Indicator/question definition or question | b. Are meetings to review progress against annual plans held on a quarterly basis at the facility level? (select one) |
| Numerator/denominator or answer choices | - yes, country-wide - yes, in some regions (please specify) - yes, is only being piloted - no - do not know   comments or clarifications |
| Unit of measurement | categorical |
| Indicator/question definition or question | c. Are clinical patient records from generalist medical practitioners used to identify health needs or priorities for health policy at the following levels of planning?   - practice/network level (select one) - regional level (select one) - country-wide (select one) |
| Numerator/denominator or answer choices | - routinely (health statistics) - incidentally - seldom - never |
| Unit of measurement | categorical |
| Rationale | A clear mandate and authority to plan care for a defined population has been shown to be a key predictor for the degree to which national plans are tailored to apply to a specific context. Managing planning processes sub-nationally has supported the strength of local partnerships, bringing unique and meaningful links across sectors for service provision. Moreover, adopting a results-orientation ensures the management of services purposefully promotes a high standard of care through the critical review of clinical and managerial processes [116]. The effect of primary care on improving equity on health depends on the availability of information about the needs in the various areas in which primary care practices are located. Targeting services around locally defined needs is effective in improving the quality and responsiveness of primary care [95]. |
| Preferred data sources | - key informant |
| Disaggregation | none specified |
| Limitations | none specified |

| Domain | Model of care |
| --- | --- |
| Subdomain | Primary care quality improvement |
| Feature | National or regional primary care performance assessment |
| **Indicator/question title** | **Accountability for performance (imp1q78)** |
| Indicator/question definition or question | a. Is primary care performance assessment carried out? (select one)   - nationally - regionally |
| Numerator/denominator or answer choices | - yes, recurrently - yes, one-off/occasionally - no - do not know   comments or clarifications |
| Unit of measurement | categorical |
| Indicator/question definition or question | b. If yes, please provide the following information and upload the relevant document |
| Numerator/denominator or answer choices | - name - type of assessment |
| Unit of measurement | upload most recent |
| Rationale | Reports on performance and health system monitoring influence health service quality [67], [68]. Reporting on the performance of health services is an important input in order for patients to exercise their freedom of choice. |
| Preferred data sources | - Health Systems Performance Assessment Working Group on Primary Care Questionnaire - Health Systems in Transition series - policy and programme documents - key informant |
| Disaggregation | none specified |
| Limitations | none specified |
|  |  |
| Domain | Model of primary care |
| Subdomain | Primary care quality improvement |
| Feature | National or regional primary care performance assessment |
| **Indicator/question title** | **Patient experience measures (imp1q79)** |
| Indicator/question definition or question | Are patient experiences measured? (select one) |
| Numerator/denominator or answer choices | - regularly, country-wide - incidentally, country-wide - regularly at local or regional level - incidentally at local or regional level - regularly at facility-level - incidentally at facility-level - no - do not know   comments or clarifications |
| Unit of measurement | categorical |
| Rationale | Surveys of patient satisfaction and utilization of health services are useful tools for obtaining information on the quality and responsiveness of health services. Such surveys may measure inputs (including whether facilities are properly equipped with essential medicines), processes (including whether waiting times are reasonable and treatment protocols are followed) and outcomes (including whether medical interventions reduce morbidity and mortality). Hence, an indicator that measures whether consumer satisfaction is considered in the assessment of health services reflect the responsiveness of the system [1]. The collection and reporting on patient experience measures is an important input to patients exercising their freedom of choice. |
| Preferred data sources | - Availability of national health services delivery data across the WHO European Region: scanning survey results - Health Systems in Transition series - policy and programme documents - key informant |
| Disaggregation | none specified |
| Limitations | none specified |

| Domain | Model of primary care |
| --- | --- |
| Subdomain | Primary care quality improvement |
| Feature | National or regional primary care performance assessment |
| **Indicator/question title** | **Job satisfaction (imp1q84)** |
| Indicator/question definition or question | Has job satisfaction of primary care providers been measured and reported? (select one) |
| Numerator/denominator or answer choices | - regularly, country-wide - incidentally, country-wide - regularly at local or regional level - incidentally at local or regional level - regularly at facility-level - incidentally at facility-level - no - do not know   comments or clarifications |
| Unit of measurement | categorical |
| Rationale | Job satisfaction has been found linked to levels of productivity, recruitment and retention, absenteeism and overall levels of quality of care [94]. Measures to assess the satisfaction of health professionals are a recognized tool to support competency-based practice environments. |
| Preferred data sources | Health Systems Performance Assessment Working Group on Primary Care  key informant |
| Disaggregation | none specified |
| Limitations | none specified |

| Domain | Model of primary care |
| --- | --- |
| Subdomain | Primary care quality improvement |
| Feature | Practice level quality improvement mechanisms |
| **Indicator/question title** | **Quality of care processes (imp2q80)** |
| Indicator/question definition or question | 1. Is there a national policy/strategy/order that requires the following quality of care processes to be implemented in primary care?  - quality improvement teams (select one) - periodic health audits (select one) - patient complaints systems (select one) - peer review meetings (select one) - incident reporting (select one) |
| Numerator/denominator or answer choices | - yes, specify policy/strategy/order - no |
| Unit of measurement | categorical |
| Indicator/question definition or question | 1. Are the following processes assuring quality of care implemented?  - quality improvement teams (select one) - periodic health audits (select one) - patient complaints systems (select one) - peer review meetings (select one) - incident reporting (select one) |
| Numerator/denominator or answer choices | - yes, country-wide - yes, in some regions (please specify) - yes, in some facilities - yes, is only being piloted - no - do not know |
| Unit of measurement | categorical |
| Rationale | Processes to assure that care is in accordance with defined standards are essential for systematically examining services across the care pathway, mapping clinical processes to identify gaps, causes of variation and to test improvements necessary. Feedback on clinical practice has an important impact on the ability of health professionals to modify their practice where evaluations show inconsistencies with a desired target [15]. |
| Preferred data sources | - Health Systems in Transition series - policy and programme documents - key informant |
| Disaggregation | none specified |
| Limitations | none specified |
|  |  |
| Domain | Model of primary care |
| Subdomain | Primary care quality improvement |
| Feature | Practice level quality improvement mechanisms |
| **Indicator/question title** | **Safety incidents reporting (imp2q81)** |
| Indicator/question definition or question | Are primary care health professionals and/or patients encouraged to report on safety incidents, near misses and safety concerns in primary care?   - primary care health professionals (select one) - patients (select one) |
| Numerator/denominator or answer choices | - yes - no - do not know   comments or clarifications |
| Unit of measurement | categorical |
| Rationale | A continuous and iterative reflection process to care contrasts with approaches that direct blame for medical errors and compromise patient safety onto individual health professional and their performance. Creating a system of reporting and learning promotes a culture of learning and ensures basic standards of care are maintained [116], [155]. |
| Preferred data sources | - Health Systems in Transition series - policy and programme documents - key informant |
| Disaggregation | none specified |
| Limitations | none specified |
|  |  |
| Domain | Model of primary care |
| Subdomain | Primary care quality improvement |
| Feature | External accountability for quality of care |
| **Indicator/question title** | **External accountability for quality of care delivered by generalist medical practitioners (imp3q82)** |
| Indicator/question definition or question | a. Is the activity of generalist medical practitioners monitored at least once a year for the following?   - volume of activity (select one) - volume of prescriptions (select one) - compliance with guidelines (select one) - performance targets (select one) - other (please specify) |
| Numerator/denominator or answer choices | - yes - no - do not know   comments and clarifications |
| Unit of measurement | categorical |
| Indicator/question definition or question | b. Do stakeholders receive this information? (select one) |
| Numerator/denominator or answer choices | - yes - no - do not know   comments or clarifications |
| Unit of measurement | categorical |
| Rationale | Standardized approaches for the measurement of quality of care across levels of care have been found to resolve sub-optimal performance from across the service continuum and not simply moving them downstream. Clinical governance ensures the impact of services is assessed and the cycle of review and reflection adds to a culture of innovation and learning [15]. |
| Preferred data sources | - key informant |
| Disaggregation | none specified |
| Limitations | none specified |

| Domain | Model of primary care |
| --- | --- |
| Subdomain | Primary care quality improvement |
| Feature | Continuous professional development |
| **Indicator/question title** | Continuous professional development opportunities (imp4q83) |
| Indicator/question definition or question | a. Have the following cadres attended any continuous professional development in the previous 12 months?  generalist medical practitioners (select one)  managers working in primary care (non-clinical professional development) (select one)  nurses working in primary care (select one)  narrow specialists working in primary care (select one)  other working in primary care, please specify (select one) |
| Numerator/denominator or answer choices | yes  no  do not know |
| Unit of measurement | category |
| Indicator/question definition or question | b. Percent of health professionals who attended any continuous professional development in the previous 12 months  generalist medical practitioners (select one)  managers working in primary care  nurses working in primary care  narrow specialists working in primary care  other working in primary care, specified in point a |
| Numerator/denominator or answer choices | **Numerator:** number of health professionals in the denominator who attended any continuous professional development  **Denominator:** number of practising health professionals in the respective category (the number should be at the end of the calendar year)  Alternate answer choices if exact data is not available:   - 70% or more - more than 50% but less than 70% - 10% to 50%   less than 10%  do not know |
| Unit of measurement | percent or category |
| Rationale | Continuous professional development is the most widely used approach to effectively improve clinical practice. There is substantial evidence that investments in different types of clinical education lead to improvements in services delivery, the consolidation of taught knowledge and skills from initial education and ultimately, improved health outcomes [116]. |
| Preferred data sources | registries of health professionals  health facility staffing routine data  expert consensus |
| Disaggregation | none specified |
| Limitations | none specified |

| Domain | Care contact |
| --- | --- |
| Subdomain | Utilization |
| Feature | Consultation rate |
| **Indicator/question title** | **Overall utilization of primary care services (utl1q85)** |
| Indicator/question definition or question | a. Average number of outpatient consultations with a generalist medical practitioner per person per year |
| Numerator/denominator or answer choices | Administrative data:  **Numerator:** total number of outpatient consultations with a generalist medical practitioner by an adult (15+ years) during the 12-month reference period (excluding telephone and email contacts, visits for prescribed laboratory tests, and visits to perform prescribed and scheduled treatment procedures, e.g. injections, physiotherapy, etc.)  **Denominator:** resident population (15+ years) |
| Unit of measurement | number of contacts with generalist medical practitioner per person per year |
| Indicator/question definition or question | b. Percent of population that consulted a primary health care team member at least once during the year (at least one outpatient consultation) |
| Numerator/denominator or answer choices | Administrative data:  **Numerator**: total number of individuals (15+ years) who consulted a primary health care team member at least once during the year (excluding telephone and email contacts, visits for prescribed laboratory tests, and visits to perform prescribed and scheduled treatment procedures, e.g. injections, physiotherapy, etc.)  **Denominator**: resident population (15+ years)  Reported survey data:  less than 1 year as self-reported time elapsed since last outpatient consultation with a primary health care team member |
| Unit of measurement | percent |
| Indicator/question definition or question | c. Percent of population attached to a primary care facility that consulted a primary health care team member at least once during the year |
| Numerator/denominator or answer choices | **Numerator**: total number of individuals in the denominator who consulted a primary care team member at least once during the year (excluding telephone and email contacts, visits for prescribed laboratory tests, and visits to perform prescribed and scheduled treatment procedures, e.g. injections, physiotherapy, etc.)  **Denominator**: number of adult individuals (15+ years) attached to a primary care facility (attachment can be either based on geographical area or on a list of enrolled patients) |
| Unit of measurement | percent |
| Rationale | Average number of outpatient consultations per person per year is part of the list of WHO recommended core indicators to evaluate health services delivery [106]. The value of the indicator is two-fold: one, it identifies outliers in the Region for which further inquiry will reveal the particular situation; and comparisons across time within countries will help to ascertain the effects of reforms or other changes. As well, the consumption of care (in terms of outpatient consultations/visits) is an indication of accessibility of services which is associated with improvements in the level of population health. |
| Preferred data sources | - health information system - survey – population - Eurostat (hlth_ehis_am1e for part b.) - UN World Population Prospects for denominator |
| Disaggregation | none specified |
| Limitations | Administrative sources tend to estimate higher average values compared to surveys because of incorrect recall. While this is an important measure of efficiency of the primary care workforce performance, the interpretation of levels across countries is ambiguous. For example, at national level, the frequency of outpatient consultations/visits will be a function of several factors including the density generalist medical practitioners in the population, the mechanism for reimbursing the generalist medical practitioner (i.e., fee-for-service payments will likely result in higher average number of outpatient consultations/visits), and the availability of other health professionals in the health workforce i.e., nurses and generalist medical practitioner assistants may fulfil basic generalist medical practitioner functions in some countries. |

| Domain | Care contact |
| --- | --- |
| Subdomain | Utilization |
| Feature | Preventive care and diagnostic services |
| **Indicator/question title** | **Influenza vaccination coverage (utl2q86)** |
| Indicator/question definition or question | Percent of at risk population who received an annual influenza vaccination:   - pregnant women - clinical risk groups - residents of long-term care facilities - population 65+ years   Note: the question should be answered if the answer to the indicator Model of care/preventive services/influenza is “yes” |
| Numerator/denominator or answer choices | Exact percent from programme/survey data  Alternate answer choices if exact data is not available:   - 70% or more - more than 50% but less than 70% - 10% to 50% - less than 10% - do not know |
| Unit of measurement | percent of target population or category |
| Rationale | Vaccines are safe, effective and the principal measure for preventing influenza and reducing the impact of epidemics. Increasing seasonal influenza vaccination uptake among these groups (high risk groups) is a key strategy to reduce the burden of influenza in the WHO European Region [156]. This approach is in line with measures to improve vertical equity by way of ensuring those who are at greatest risk are treated accordingly. This measures effectiveness and quality of primary health care and preventive services. Focusing on targeted groups presents delivery and coordination challenges since some may be more difficult to reach if they are not accessing health services. On the other hand, if there is any interface with health or social services then effective coordination would ensure high rates of vaccination coverage. There is some evidence that influenza vaccine reduces exacerbations in chronic obstructive pulmonary disease individuals [157]. |
| Preferred data sources | - European Centre for Disease Prevention and Control – Seasonal influenza vaccination in Europe Technical Report for 2014-2015 - European Core Health Indicators – influenza vaccination rates for people 65+ years - OECD Data – influenza vaccination rates for people 65+ years - health information system - Health Systems in Transition series - expert consensus |
| Disaggregation | none specified |
| Limitations | Unfortunately, this information is not collected for the entire WHO European Region, and it is not disseminated on the HFA-DB. |
|  |  |
| Domain | Care contact |
| Subdomain | Utilization |
| Feature | Preventive care and diagnostic services |
| **Indicator/question title** | **HPV vaccination coverage (utl2q87)** |
| Indicator/question definition or question | Percent of population targeted by the national HPV vaccination programme who were successfully vaccinated (select one)  Note: the question should be answered if the answer to the indicator Model of care/Preventive services/HPV vaccination is “yes”. The percentage reflect the target population of the national HPV vaccination programme – boys and girls, or only girls. |
| Numerator/denominator or answer choices | Exact percent from survey/programme data  Alternate answer choices if exact data is not available:   - 70% or more - more than 50% but less than 70% - 10% to 50% - less than 10% - do not know |
| Unit of measurement | percent of target population or category |
| Rationale | As part of a more comprehensive approach to cervical cancer prevention and control, HPV vaccination plays an important role in protecting adolescent girls and young women [157]. |
| Preferred data sources | - WHO Global Country Capacity and Response Survey on Noncommunicable Diseases Survey 2017 - health information system - expert consensus |
| Disaggregation | none specified |
| Limitations | The target population varies by country. |

| Domain | Care contact |
| --- | --- |
| Subdomain | Utilization |
| Feature | Preventive care and diagnostic services |
| **Indicator/question title** | **Diabetic education (ult2q88)** |
| Indicator/question definition or question | Percent of individuals registered for diabetes treatment who were referred for diabetic education |
| Numerator/denominator or answer choices | **Numerator:** number of cases in the denominator who were referred for diabetic education  **Denominator:** number of individuals registered for treatment of diabetes during the quarter that ended 6 months previously  Alternate answer choices if exact data is not available:   - 70% or more - more than 50% but less than 70% - 10% to 50% - less than 10% - do not know |
| Unit of measurement | percent or category |
| Rationale | Training for self-management strategies in people with diabetes type 2 is effective in improving fasting blood glucose levels, glycated hemoglobin and diabetes knowledge and in reducing systolic blood pressure levels, body weight and the requirement for diabetes medication. |
| Preferred data sources | - health information system - survey – population - expert consensus |
| Disaggregation | none specified |
| Limitations | None specified. |

| Domain | Care contact |
| --- | --- |
| Subdomain | Utilization |
| Feature | Preventive care and diagnostic services |
| **Indicator/question title** | **Counselling services for tobacco cessation (utl2q89)** |
| Indicator/question definition or question | Percent of population who are smokers who were advised by a primary care health professional to quit smoking in the previous 12 months |
| Numerator/denominator or answer choices | Exact percent reported in survey analysis  Alternate answer choices if exact data is not available:  70% or more  more than 50% but less than 70%  10% to 50%  less than 10% |
| Unit of measurement | percent of target population |
| Rationale | Evidence-based support to quit tobacco use (tobacco dependence treatment) includes methods from simple medical advice to pharmacotherapy, along with quit lines and counselling. However, tobacco users have low levels of awareness of the evidence about these tobacco dependence treatment interventions. This indicator would measure the ability of preventive care efforts to reach the population intended [158]. |
| Preferred data sources | WHO STEPwise approach to surveillance survey  survey – population |
| Disaggregation | none specified |
| Limitations | none specified |

| Domain | Care contact |
| --- | --- |
| Subdomain | Utilization |
| Feature | Preventive care and diagnostic services |
| **Indicator/question title** | **National cancer screening programmes targeting the general population (utl2q90)** |
| Indicator/question definition or question | a. Percent of target female population who had cervical cancer screening  Note: Cervical cancer screening includes a Papanicolau test, an HPV test or a visual inspection with acetic acid; target population according to screening frequencies corresponding to national cancer screening programme and policies |
| Numerator/denominator or answer choices | Exact percent from programme/survey data  Alternate answer choices if exact data is not available:   - 70% or more - more than 50% but less than 70% - 10% to 50% - less than 10% - do not know |
| Unit of measurement | categorical |
| Indicator/question definition or question | b. Percent of target female population who were screened for breast cancer  Note: Breast cancer screening includes bilateral mammography; target population according to screening frequencies corresponding to national cancer screening programme and policies |
| Numerator/denominator or answer choices | Exact percent from programme/survey data  Alternate answer choices if exact data is not available:   - 70% or more - more than 50% but less than 70% - 10% to 50% - less than 10% - do not know |
| nit of measurement | categorical |
| Indicator/question definition or question | c. Percent of target population who were screened for colon cancer  Note: colon cancer screening includes faecal test or a colonoscopy/sigmoidoscopy; target population according to screening frequencies corresponding to national cancer screening programme and policies |
| Numerator/denominator or answer choices | Exact percent from programme/survey data  Alternate answer choices if exact data is not available:   - 70% or more - more than 50% but less than 70% - 10% to 50% - less than 10% - do not know |
| Unit of measurement | categorical |
| Rationale | The cervical cancer screening indicator is indicator 25 of the NCD Global Monitoring Framework for noncommunicable diseases which will track the implementation of the noncommunicable diseases action plan through monitoring and reporting on the attainment of the global targets in 2015-2020. The 25 indicators and the 9 voluntary global targets of the framework provide overall direction and the action plan provides a road map for reaching the targets [122]. More information specifically on this indicator and methods for calculation is available at http://www.who.int/nmh/ncd-tools/indicator25/en/. |
| Preferred data sources | - WHO Global Country Capacity and Response Survey on Noncommunicable Diseases Survey 2017 - health information system - expert consensus |
| Disaggregation | none specified |
| Limitations | Data not specific to primary care. WHO Member States agreed to an indicator regarding monitoring the proportion of women between the ages 30-49 years screened for cervical cancer at least once, or more often, and lower or higher age groups according to national programmes and policies [159]. The WHO Noncommunicable country capacity survey collects information on screening coverage according to national programmes and policies without imposing an age bracket or frequency [120]. OECD reports programme and survey data for cervical cancer screening for women 20-69 years, within the past 3 years (or according to the specific screening frequency recommended in each country) [160]. |

| Domain | Care contact |
| --- | --- |
| Subdomain | Utilization |
| Feature | Preventive care and diagnostic services |
| **Indicator/question title** | **Individual risk assessments (utl2q91)** |
| Indicator/question definition or question | Percent of population, age 40-64, with cardiovascular disease risk assessment |
| Numerator/denominator or answer choices | **Numerator:** number of individuals in the denominator whose records include a cardiovascular disease risk assessment/screening  **Denominator:** number of individuals aged 40-64 years  Alternate answer choices if exact data is not available:   - 70% or more - more than 50% but less than 70% - 10% to 50% - less than 10% - do not know |
| Unit of measurement | percent of population aged 40-64 years or category |
| Rationale | Cardiovascular risk assessment is one of the three individual level priority interventions in the Action Plan for Prevention and Control of Noncommunicable Diseases in the WHO European Region [157], [114]. |
| Preferred data sources | - health information system - expert consensus |
| Disaggregation | service provided in primary care/outside of primary care |
| Limitations | none specified |
|  |  |
| Domain | Care contact |
| Subdomain | Utilization |
| Feature | Preventive care and diagnostic services |
| **Indicator/question title** | **Tuberculosis preventive care and diagnostic services (utl2q91)** |
| Indicator/question definition or question | Percent of risk groups with systematic screening for active tuberculosis and latent tuberculosis infection among tuberculosis risk groups |
| Numerator/denominator or answer choices | **Numerator:** actual number of people screened for tuberculosis and/or latent tuberculosis infection in a defined period **Denominator:** total number of people at risk eligible for screening according to the national guidelines, in the same period |
| Unit of measurement | percent |
| Rationale | This is an indicator from the Roadmap to implement the tuberculosis action plan for the WHO European region, with full coverage target [57]. Systematic screening is one of the four components of pillar 1 of the End TB strategy focused on integrated, person-centred care and prevention [161]. The screening tests, examinations or other procedures should efficiently distinguish persons with a high probability of having tuberculosis (that is, with suspected TB) from those who are unlikely to have TB. Among those whose screening is positive, the diagnosis needs to be established by using one or several diagnostic tests and additional clinical assessments, which together have high accuracy [162]. This approach is in line with measures to improve vertical equity by way of ensuring those who are at greatest risk are treated accordingly. |
| Preferred data sources | data reported in WHO Global Tuberculosis Report 2017 |
| Disaggregation | age groups (0-4 years, 5-14 years and 15+ years)  risk factors: people living with HIV (PLHIV), prisoners, migrants, other according to national guidelines. |
| Limitations | Indiscriminate mass screening should be avoided. The prioritization of risk groups for screening should be based on assessments made for each risk group of the potential benefits and harms, the feasibility of the initiative, the acceptability of the approach, the number needed to screen, and the cost effectiveness of screening.  The choice of algorithm for screening and diagnosis is country specific and should be based on an assessment of the accuracy of the algorithm for each risk group considered, as well as the availability, feasibility and cost of the tests. |

| Domain | Care contact |
| --- | --- |
| Subdomain | Utilization |
| Feature | Preventive care and diagnostic services |
| **Indicator/question title** | **WHO recommended rapid test as the initial diagnostic test for tuberculosis (utl2q93)** |
| Indicator/question definition or question | Percent of notified new and relapse tuberculosis cases tested with a WHO recommended rapid test as the initial diagnostic test |
| Numerator/denominator or answer choices | **Numerator:** number of notified new and relapse tuberculosis cases tested with a WHO recommended rapid diagnostic test as the initial test during the reference period  **Denominator:** number of notified new and relapse tuberculosis cases during the reference period |
| Unit of measurement | percent |
| Rationale | This indicator is in line with the recommendation of WHO to replace by 2017 the initial diagnostic test for all people with signs and symptoms of tuberculosis with a new point of care WHO-recommended rapid diagnostics with sensitivity similar to that of liquid culture. WHO will monitor this indicator in low- and middle-income countries. a target of 100% should be reached by the end of 2018 for people living with HIV and people at risk of DR-TB. This indicator is also included as one of the top 10 priority indicators for monitoring the implementation of the End tuberculosis Strategy [163], [119], [164], [161]. |
| Preferred data sources | - data reported in WHO Global tuberculosis report 2017; indicator available in country profiles as “% tested with rapid diagnostics at time of diagnosis” |
| Disaggregation | Where electronic registers or periodic surveys allow stratification, national-level monitoring of this indicator should be stratified by patient risk group. |
| Limitations | none specified |

| Domain | Care contact |
| --- | --- |
| Subdomain | Continuity of primary care |
| Feature | Treatment |
| **Indicator/question title** | **Hypertension treatment coverage (con1q94)** |
| Indicator/question definition or question | Percent of hypertensive individuals with controlled blood pressure |
| Numerator/denominator or answer choices | **Numerator:** cumulative number of registered patients with controlled blood pressure (SBP<140 and DBP<90) at all health facilities, aged 18+  **Denominator:** estimated number of individuals aged 18+ years with a diagnosis of hypertension  Alternate answer choices if exact data is not available:   - 70% or more - more than 50% but less than 70% - 10% to 50% - less than 10% - do not know |
| Unit of measurement | percent of target population or category |
| Rationale | This indicator is part of the Systems for monitoring of the HEARTS Technical package for cardiovascular disease management in primary health care. Its purpose is to measure the coverage of the programme to treat and control hypertension [131]. |
| Preferred data sources | - health information system (numerator) - registers for hypertension (numerator) - STEPwise approach to surveillance or similar survey (denominator) - expert consensus |
| Disaggregation | initial treatment prescribed in primary care/outside of primary care |
| Limitations | none specified |

| Domain | Care contact |
| --- | --- |
| Subdomain | Continuity of primary care |
| Feature | Treatment |
| **Indicator/question title** | **Tuberculosis treatment coverage (con1q95)** |
| Indicator/question definition or question | Percent of estimated number of incident tuberculosis cases that were notified and treated |
| Numerator/denominator or answer choices | **Numerator:** number of new and relapse cases that were notified and treated  **Denominator:** estimated number of incident tuberculosis cases in the same year |
| Unit of measurement | percent |
| Rationale | This indicator measures the capacity of health system to ensure anti-tuberculosis treatment and assure rapid and quality care. In low resources settings and with weak tuberculosis governance as well with gaps in pharmaceutical management detected tuberculosis cases remain in the waiting lists for and when available treatment. The target for coverage is 90% or more. |
| Preferred data sources | - data reported in WHO Global tuberculosis report 2017, country profile “TB treatment coverage (notified/estimated incidence)” |
| Disaggregation | - all tuberculosis - HIV-status - rifampicin resistant/multidrug resistant tuberculosis conf_rrmdr_tx/conf_rrmdr |
| Limitations | none specified |

| Domain | Care contact |
| --- | --- |
| Subdomain | Continuity of primary care |
| Feature | Treatment |
| **Indicator/question title** | **Depression treatment coverage (con1q96)** |
| Indicator/question definition or question | Percent of population aged 18+ years with a diagnosis of depression who were offered antidepressant drug treatment or referral to a mental health professional |
| Numerator/denominator or answer choices | **Numerator:** number of individuals in the denominator who were diagnosed and offered psychological or antidepressant drug treatment or referral to a mental health professional by a generalist medical practitioner in the previous 12 months.  **Denominator:** estimated prevalence of depression (number of individuals aged 18+ years)  Alternate answer choices if exact data is not available:   - 70% or more - more than 50% but less than 70% - 10% to 50% - less than 10% - do not know |
| Unit of measurement | percent of target population or category |
| Rationale | WHO Mental Health Action Plan 2013-2020 objective no 2 specifies to provide comprehensive, integrated and responsive mental health and social care services in community-based settings. Among the actions suggested is the reorganization of services to shift the locus of care away from long-stay mental hospitals towards non-specialized health settings, with increasing coverage of evidence based interventions which can be delivered, among other settings, in primary care [165]. |
| Preferred data sources | - health information system - expert consensus |
| Disaggregation | N/A |
| Limitations | none specified |

| Domain | Care contact |
| --- | --- |
| Subdomain | Continuity of primary care |
| Feature | Follow-up care |
| **Indicator/question title** | **Hypertension follow-up (con2q97)** |
| Indicator/question definition or question | Percent of hypertensive individuals aged 18+ years who had a follow-up consultation in primary care (excluding visits only for medication re-fill) in the 12-month reference period |
| Numerator/denominator or answer choices | **Numerator:** number of individuals in the denominator who had a follow-up consultation with a generalist medical practitioner in the 12-month reference period  **Denominator:** estimated number of individuals aged 18+ years with a diagnosis of hypertension  Alternate answer choices if exact data is not available:   - 70% or more - more than 50% but less than 70% - 10% to 50% - less than 10% - do not know |
| Unit of measurement | percent of target population or category |
| Rationale | Measuring this gap reflects the health system’s continuity, including the system’s ability to capture and follow-up with patients. |
| Preferred data sources | - health information system - STEPwise approach to surveillance (denominator) - expert consensus |
| Disaggregation | none specified |
| Limitations | none specified |

| Domain | Care contact |
| --- | --- |
| Subdomain | Continuity of primary care |
| Feature | Follow-up care |
| **Indicator/question title** | **Diabetes monitoring (con2q98)** |
| Indicator/question definition or question | Percent of diabetic type 2 population aged 18+ years who were monitored in primary care in the previous year by receiving the following tests:   - foot exam - eye exam - urine protein test - blood pressure measurement - overweight screening |
| Numerator/denominator or answer choices | **Numerator:** number of individuals in the denominator who received the respective exams/tests during a visit with a primary care professional in the 12-month reference period or otherwise specified  **Denominator:** number of individuals aged 18+ years diagnosed with diabetes type 2  Alternate answer choices if exact data is not available:   - 70% or more - more than 50% but less than 70% - 10% to 50% - less than 10% - do not know |
| Unit of measurement | percent of target population or category |
| Rationale | Diabetes is a primary care sensitive condition. The provision of a wide range of services provided by primary care health professionals is associated with better health outcomes at lower costs. These are part of the essential package of interventions for diabetic patients from WHO-PEN (foot exam, and eye exam) [96]. Early detection and treatment of complications (at intervals recommended by national and international guidelines) is an important part of managing diabetes in primary care [114]. |
| Preferred data sources | - health information system - expert consensus |
| Disaggregation | none specified |
| Limitations | none specified |

| Domain | Care contact |
| --- | --- |
| Subdomain | Continuity of primary care |
| Feature | Follow-up care |
| **Indicator/question title** | **Chronic obstructive pulmonary disease follow-up (con2q99)** |
| Indicator/question definition or question | Percent of individuals aged 18+ years with chronic obstructive pulmonary disease who had a follow-up consultation with a generalist medical practitioner in the previous 12 months   - general follow-up consultation - lung function measurement |
| Numerator/denominator or answer choices | **Numerator:** number of individuals in the denominator who had a follow-up consultation, including a lung function measurement, with a generalist medical practitioner for chronic obstructive pulmonary disease in the 12-month reference period  **Denominator:** number of individuals aged 18+ years diagnosed with chronic obstructive pulmonary disease  Alternate answer choices if exact data is not available:   - 70% or more - more than 50% but less than 70% - 10% to 50% - less than 10% - do not know |
| Unit of measurement | percent of target population or category |
| Rationale | Measuring this gap reflects the health system’s continuity, including the system’s ability to capture and follow-up with patients. |
| Preferred data sources | - health information system - expert consensus |
| Disaggregation | none specified |
| Limitations | none specified |

| Domain | Care contact |
| --- | --- |
| Subdomain | Continuity of primary care |
| Feature | Follow-up care |
| **Indicator/question title** | **Post-natal care (con2q100)** |
| Indicator/question definition or question | Percent of women who received a post-natal health check   - between days 7-14 post delivery - 6 weeks post deliver |
| Numerator/denominator or answer choices | **Numerator:** number of women in the denominator who received a health check in primary care during the specified intervals post-delivery, in the 12-month reference period:  ICD-10 Z39.2 - encounter for routine postpartum follow-up  ICPC2 - W31 - postnatal check-up  **Denominator:** number of women, age 15 to 49, who had a delivery in the 12-month reference period  Alternate answer choices if exact data is not available:   - 70% or more - more than 50% but less than 70% - 10% to 50% - less than 10% - do not know |
| Unit of measurement | percent of women who had a delivery in the 12-month reference period or category |
| Rationale | In 2013, there was a notable change to existing WHO guidance on postnatal check-up for mothers to include 4 postnatal check-ups: full assessment during the first day, and three check-ups: on day 3 (48-72 hours), between days 7-14, and 6 weeks after birth. These contacts can be made at home or in health facility, depending on the context and the provider. Additional contacts may be needed to address issues or concerns [166]. |
| Preferred data sources | - health information system - expert consensus |
| Disaggregation | none specified |
| Limitations | none specified |

| Domain | Care contact |
| --- | --- |
| Subdomain | Continuity of primary care |
| Feature | Follow-up care |
| **Indicator/question title** | **Depression treatment follow-up (con2q101)** |
| Indicator/question definition or question | Percent of population aged 18+ years with depression who received psychological treatment or were prescribed anti-depressant drug treatment by a generalist medical practitioner and who had a follow-up consultation with the generalist medical practitioner |
| Numerator/denominator or answer choices | **Numerator:** number of individuals in the denominator who had a follow-up consultation with a generalist medical practitioner for review within two to four weeks of initiating psychological or antidepressant drug treatment  **Denominator:** number of individuals aged 18+ years with depression who started anti-depressant drug treatment in the 12-month reference period under the supervision of a generalist medical practitioner  Alternate answer choices if exact data is not available:   - 70% or more - more than 50% but less than 70% - 10% to 50% - less than 10% - do not know |
| Unit of measurement | percent of target population or category |
| Rationale | In adult individuals with depressive episode/disorders who have benefited from psychological or initial antidepressant treatment, the psychological or antidepressant treatment should not be stopped before 9 -12 months after recovery. Treatment should be regularly monitored, with special attention to treatment adherence. Frequency of contact should be determined by the adherence, severity and by local feasibility issues [167]. |
| Preferred data sources | - health information system - expert consensus |
| Disaggregation | none specified |
| Limitations | WHO mental health guidelines focus on treatment for moderate to severe depression, and not mild depression. |

| Domain | Care contact |
| --- | --- |
| Subdomain | Continuity of primary care |
| Feature | Longitudinal continuity of care |
| **Indicator/question title** | **Stability of patient–generalist medical practitioner relationship (con3q102)** |
| Indicator/question definition or question | Percent of population who report visiting their usual generalist medical practitioner for their common health problems |
| Numerator/denominator or answer choices | Exact percent reported in survey analysis  Alternate answer choices if exact data is not available:  70% or more  more than 50% but less than 70%  10% to 50%  less than 10% |
| Unit of measurement | percent of population |
| Rationale | The existence of an ongoing relationship with a particular generalist medical practitioner rather than with a particular place or no place at all, is beneficial for the quality of care [95]. |
| Preferred data sources | survey – population |
| Disaggregation | none specified |
| Limitations | none specified |

| Domain | Care contact |
| --- | --- |
| Subdomain | Continuity of primary care |
| Feature | Informational continuity of care |
| **Indicator/question title** | **Medical record keeping (con4q103)** |
| Indicator/question definition or question | Percent of generalist medical practitioners with complete medical records for all patients |
| Numerator/denominator or answer choices | Exact percent reported in survey analysis  Alternate answer choices if exact data is not available:  70% or more  more than 50% but less than 70%  10% to 50%  less than 10%  do not know |
| Unit of measurement | percent of practitioners or category |
| Rationale | Systematically keeping medical records is an important measure to achieve informational continuity of care and to facilitate personalized care provision. Both are important for the quality of care [95]. |
| Preferred data sources | Commonwealth Fund International Survey of Primary Care Physicians in 10 Nations [36]  survey – health professionals  expert consensus |
| Disaggregation | none specified |
| Limitations | none specified |
|  |  |
| Domain | Care contact |
| Subdomain | Continuity of primary care |
| Feature | Informational continuity of care |
| **Indicator/question title** | **Incoming clinical information procedures (con4q104)** |
| Indicator/question definition or question | Percent of generalist medical practitioners who receive information/notification when their patients have contacted out-of-hours services, including emergency care |
| Numerator/denominator or answer choices | Exact percent reported in survey analysis  Alternate answer choices if exact data is not available:  70% or more  more than 50% but less than 70%  10% to 50%  less than 10% |
| Unit of measurement | exact percent if available, otherwise categorical |
| Rationale | To safeguard the quality of care it is important that the generalist medical practitioner receives feedback on patient results of the visits to other care providers, during or after office hours. Besides the necessity for generalist medical practitioners to stay up to date on the progress of their patients, individuals find it easier to obtain information from their regular source of care compared to a specialist medical practitioner [95]. |
| Preferred data sources | Commonwealth Fund - International survey of primary care physicians in 10 nations [36]  survey – health professionals  expert consensus |
| Disaggregation | none specified |
| Limitations | none specified |
|  |  |
| Domain | Care contact |
| Subdomain | Continuity of primary care |
| Feature | Informational continuity of care |
| **Indicator/question title** | **Generalist–specialist medical practitioner communication (con4q105)** |
| Indicator/question definition or question | Percent of generalist medical practitioners who always receive a report/reply letter back from specialist medical practitioner with all relevant health information |
| Numerator/denominator or answer choices | Exact percent reported in survey analysis  Alternate answer choices if exact data is not available:  70% or more  more than 50% but less than 70%  10% to 50%  less than 10% |
| Unit of measurement | percent or category |
| Rationale | To safeguard the quality of care it is important that the generalist medical practitioner receives feedback on patient results of the visits to other health professionals, during or after office hours. Besides the necessity for primary care health professionals to stay up to date on the progress of their patients, individuals find it easier to obtain information from their regular source of care compared to a specialist medical practitioner [95]. |
| Preferred data sources | Commonwealth Fund – International survey of primary care physicians in 10 nations  survey – health professionals  expert - consensus |
| Disaggregation | none specified |
| Limitations | none specified |
|  |  |
| Domain | Care contact |
| Subdomain | Continuity of primary care |
| Feature | Informational continuity of care |
| **Indicator/question title** | **Generalist medical practitioner-social services (con4q106)** |
| Indicator/question definition or question | Percent of generalist medical practitioners who coordinate care with social services or other community providers at least once per month |
| Numerator/denominator or answer choices | Exact percent reported in survey analysis  Alternate answer choices if exact data is not available:  70% or more  more than 50% but less than 70%  10% to 50%  less than 10% |
| Unit of measurement | categorical |
| Rationale | When different types of health professionals are involved in a person’s care complete and timely information sharing will ensure safe and prompt care. |
| Preferred data sources | Commonwealth Fund - International survey of primary care physicians in 10 nations [36]  existing surveys – health professionals and assessments  expert consensus |
| Disaggregation | none specified |
| Limitations | none specified |
|  |  |
| Domain | Care contact |
| Subdomain | Coordination of care across settings |
| Feature | Transition management |
| **Indicator/question title** | **Referral feedback to primary care (cor1q108)** |
| Indicator/question definition or question | Percent of generalist medical practitioners that receive information needed to continue managing the individual upon discharge from hospital (including recommended follow-up care) within 4 days |
| Numerator/denominator or answer choices | Exact percent reported in survey analysis  Alternate answer choices if exact data is not available:  70% or more  more than 50% but less than 70%  10% to 50%  less than 10% |
| Unit of measurement | average time period |
| Rationale | Generalist medical practitioners depend on the feedback on clinical findings and further care required to care for returning patients effectively. Lack of such feedback can lead to poor efficiency and care that is not cost effective. |
| Preferred data sources | Commonwealth Fund - International survey of primary care physicians in 10 nations  survey – health professionals  expert consensus |
| Disaggregation | none specified |
| Limitations | none specified |

| Domain | Care contact |
| --- | --- |
| Subdomain | Comprehensiveness of primary care |
| Feature | Resolution capacity of generalist medical practitioners |
| **Indicator/question title** | **Generalist medical practitioner consultations without referral (cop1q110)** |
| Indicator/question definition or question | Percent of total consultations handled solely by generalist medical practitioners without referrals to other health professionals |
| Numerator/denominator or answer choices | **Numerator:** number of consultations in the denominator prescribed a referral  **Denominator:** number of first-contact consultations (include only the first consultations and exclude consultations that are for the same course of treatment)  Alternate answer choices if exact data is not available:   - 70% or more - more than 50% but less than 70% - 10% to 50% - less than 10% |
| Unit of measurement | percent or category |
| Rationale | First contact care by generalist medical practitioners is essential to address the wide variety and often very basic needs existing in the community. Having a generalist medical practitioner rather than a specialist medical practitioner as a regular source of care has been associated with better health outcomes and lower health care costs [95]. Studies have shown that in countries where generalist medical practitioners had a strong role as the doctor of first contact they treated more than 90% of all patient contacts without referral [168]. |
| Preferred data sources | - health information system - expert consensus |
| Disaggregation | none specified |
| Limitations | none specified |

| Domain | Care contact |
| --- | --- |
| Subdomain | People-centeredness of primary care |
| Feature | Patient experience |
| **Indicator/question title** | **Patient satisfaction (pcc1q111)** |
| Indicator/question definition or question | Percent of population who are overall satisfied with primary care services |
| Numerator/denominator or answer choices | Exact percent reported in survey analysis  Alternate answer choices if exact data is not available:  70% or more  more than 50% but less than 70%  10% to 50%  less than 10% |
| Unit of measurement | percent |
| Rationale | The quality of the personal relationship between patients and their generalist medical practitioners, which should be characterized by a sense of responsibility for the delivery of coordinated and comprehensive care and a mutual feeling of trust and loyalty, leads to better quality of care [95]. |
| Preferred data sources | Health Systems in Transition series  survey – population |
| Disaggregation | none specified |
| Limitations | none specified |
|  |  |
| Domain | Care contact |
| Subdomain | People-centeredness of primary care |
| Feature | Shared decision-making |
| **Indicator/question title** | **Care and treatment shared decision-making (pcc2q112)** |
| Indicator/question definition or question | Percent of population reporting the generalist medical practitioner involved them as much as they wanted to be in decisions about their care and treatment |
| Numerator/denominator or answer choices | Exact percent reported in survey analysis  Alternate answer choices if exact data is not available:  70% or more  more than 50% but less than 70%  10% to 50%  less than 10% |
| Unit of measurement | percent |
| Rationale | Patient-reported experience measures (PREMs) with primary care are an important marker of primary care quality from the point of view of those most concerned – patients themselves. |
| Preferred data sources | OECD Health Care Quality Indicators - patient experience  STEPwise approach to surveillance survey, optional module  Health Systems in Transition  survey – population |
| Disaggregation | none specified |
| Limitations | Target population of the STEPS noncommunicable diseases risk factor survey be all adults aged 18 to 69 [159]. OECD Health Care Quality Indicators report data from 16+ years age group. |
|  |  |
| Domain | Care contact |
| Subdomain | People-centeredness of primary care |
| Feature | Patient engagement |
| **Indicator/question title** | **Patient reporting opportunity to ask questions (pcc3q113)** |
| Indicator/question definition or question | Percent of population reporting generalist medical practitioner giving opportunity to ask questions or raise concerns about recommended treatment |
| Numerator/denominator or answer choices | Exact percent reported in survey analysis  Alternate answer choices if exact data is not available:  70% or more  more than 50% but less than 70%  10% to 50%  less than 10% |
| Unit of measurement | percent |
| Rationale | Patient-reported experience measures (PREMs) with primary care are an important marker of primary care quality from the point of view of those most concerned – patients themselves. The quality of the personal relationship between patients and their generalist medical practitioner, which should be characterized by a sense of responsibility for the delivery of coordinated and comprehensive care and a mutual feeling of trust and loyalty, leads to better quality of care [95]. |
| Preferred data sources | OECD Health Care Quality Indicators - patient experience  STEPwise approach to surveillance survey, optional module  survey – population |
| Disaggregation | none specified |
| Limitations | Target population of the STEPS noncommunicable diseases risk factor survey be all adults aged 18 to 69 [159]. OECD Health Care Quality Indicators report data from 16+ years age group. |
|  |  |
| Domain | Care contact |
| Subdomain | People-centeredness of primary care |
| Feature | Patient engagement |
| **Indicator/question title** | **Patient reporting enough time with doctor (pcc3q114)** |
| Indicator/question definition or question | Percent of population reporting the generalist medical practitioner spending enough time with them during the consultation |
| Numerator/denominator or answer choices | Exact percent reported in survey analysis  Alternate answer choices if exact data is not available:  70% or more  more than 50% but less than 70%  10% to 50%  less than 10% |
| Unit of measurement | Percent |
| Rationale | Patient-reported experience measures (PREMs) with primary care are an important marker of primary care quality from the point of view of those most concerned – patients themselves. The quality of the personal relationship between patients and their generalist medical practitioner, which should be characterized by a sense of responsibility for the delivery of coordinated and comprehensive care and a mutual feeling of trust and loyalty, leads to better quality of care [95]. |
| Preferred data sources | OECD Health Care Quality Indicators - patient experience  STEPwise approach to surveillance survey, optional module  survey – population |
| Disaggregation | none specified |
| Limitations | Target population of the STEPS noncommunicable diseases risk factor survey be all adults aged 18 to 69 [159]. OECD Health Care Quality Indicators report data from 16+ years age group. |
|  |  |
| Domain | Care contact |
| Subdomain | People-centeredness of primary care |
| Feature | Patient engagement |
| **Indicator/question title** | **Patient reporting easy to understand explanations (pcc3q115)** |
| Indicator/question definition or question | Percent of population reporting generalist medical practitioner providing easy-to-understand explanations |
| Numerator/denominator or answer choices | Exact percent reported in survey analysis  Alternate answer choices if exact data is not available:  70% or more  more than 50% but less than 70%  10% to 50%  less than 10% |
| Unit of measurement | percent |
| Rationale | Patient-reported experience measures (PREMs) with primary care are an important marker of primary care quality from the point of view of those most concerned – patients themselves. The quality of the personal relationship between patients and their generalist medical practitioner, which should be characterized by a sense of responsibility for the delivery of coordinated and comprehensive care and a mutual feeling of trust and loyalty, leads to better quality of care [95]. |
| Preferred data sources | OECD Health Care Quality Indicators - patient experience  STEPwise approach to surveillance survey, optional module  survey – population |
| Disaggregation | none specified |
| Limitations | Target population of the STEPS noncommunicable diseases risk factor survey be all adults aged 18 to 69 [159]. OECD Health Care Quality Indicators report data from 16+ years age group. |
|  |  |
| Domain | Outputs |
| Subdomain | Access to primary care services |
| Feature | Availability and affordability of primary care services |
| **Indicator/question title** | **Same day appointments (acc1q116)** |
| Indicator/question definition or question | Percent of population reporting that they could get a same-day or next-day appointment to see a generalist medical practitioner for immediate care for a minor health problem |
| Numerator/denominator or answer choices | Exact percent reported in survey analysis  Alternate answer choices if exact data is not available:  70% or more  more than 50% but less than 70%  10% to 50%  less than 10% |
| Unit of measurement | percent |
| Rationale | Access (in general) is the opportunity or ability to both obtain the health services people need, while benefitting from financial risk protection. Universal health coverage is not possible without universal access. Access has three domains: physical accessibility, financial affordability and acceptability. Physical accessibility is understood as the availability of good health services within reasonable reach of those who need them and of opening hours, appointment systems and other aspects of service organization and delivery that allow people to obtain the services when they need them [169]. |
| Preferred data sources | Commonwealth Fund - International profiles of health care systems  STEPwise approach to surveillance survey, optional module  survey – population |
| Disaggregation | none specified |
| Limitations | none specified |
|  |  |
| Domain | Outputs |
| Subdomain | Access to primary care services |
| Feature | Availability and affordability of primary care services |
| **Indicator/question title** | **Waiting time for appointment (acc1q117)** |
| Indicator/question definition or question | Waiting time to see a generalist medical practitioner in the facility for a booked appointment |
| Numerator/denominator or answer choices | Average number of minutes individuals waited to see a generalist medical practitioner in the facility for a booked appointment (reported in the survey analysis) |
| Unit of measurement | minutes |
| Rationale | Access (in general) is the opportunity or ability to both obtain the health services people need, while benefitting from financial risk protection. Universal health coverage is not possible without universal access. Access has three domains: physical accessibility, financial affordability and acceptability. Physical accessibility is understood as the availability of good health services within reasonable reach of those who need them and of opening hours, appointment systems and other aspects of service organization and delivery that allow people to obtain the services when they need them [169]. |
| Preferred data sources | STEPwise approach to surveillance survey, optional module  Health Systems in Transition series  survey – population |
| Disaggregation | none specified |
| Limitations | none specified |

| Domain | Outputs |
| --- | --- |
| Subdomain | Access to primary care services |
| Feature | Availability and affordability of primary care services |
| **Indicator/question title** | **Access barriers due to treatment costs (acc2q119)** |
| Indicator/question definition or question | Percent of population that reported needing a medical service but skipped them due to costs:  outpatient consultation/visits with a generalist medical practitioner  follow-up care and treatment (not medication) prescribed in primary care  medicine prescribed in primary care |
| Numerator/denominator or answer choices | Exact percent reported in survey analysis  Alternate answer choices if exact data is not available:  70% or more  more than 50% but less than 70%  10% to 50%  less than 10% |
| Unit of measurement | percent |
| Rationale | Access (in general) is the opportunity or ability to both obtain the health services people need, while benefitting from financial risk protection. Universal health coverage is not possible without universal access. Access has three domains: physical accessibility, financial affordability and acceptability. Financial affordability to primary care services is a key feature of a strong primary care system. Financial access, a measure of people's ability to pay for services without financial hardship, is a critical component of health service access. Analysing it, considers not only the price of health services, but also indirect and opportunity costs (e.g. the costs of transportation to and from facilities and of taking time away from worked). All European countries endorse equity of access to health care for all people as an important policy objective. One method of gauging to what extent this objective is achieved is through assessing reports of unmet needs for health care. The problems that people report in obtaining care when they are ill often reflect significant barriers to care [170]. |
| Preferred data sources | OECD Health Care Quality Indicators  STEPwise approach to surveillance survey, optional module  survey – population |
| Disaggregation | none specified |
| Limitations | Target population of the STEPS noncommunicable diseases risk factor survey be all adults aged 18 to 69 [159]. OECD Health care quality indicators reports data from 16+ years age group.  This indicator may not be available for primary care only, and is reported differently across data sources:  WHO STEPS optional module separates these three services and is reported specifically for primary care.  OECD's question captures doctor, nurse, or allied health professional. It is not specific to primary care. It groups medical tests, treatment and follow-up. There is a separate question on prescriptions.  European Core Health Indicators report unmet need grouping together reasons: financial barriers, waiting time and travelling distance. |

| Domain | Outputs |
| --- | --- |
| Subdomain | Access to primary care services |
| Feature | Availability and affordability of primary care services |
| **Indicator/question title** | Access to essential medicines (acc2q154) |
| Indicator/question definition or question | Proportion of health facilities that have a core set of relevant essential medicines available and affordable on a sustainable basis |
| Numerator/Denominator or answer choices | As reported for the SDG 3.b.3 indicator  For detailed computation method and methodology please refer to the metadata of indicator SDG 3.b.3 [115]. |
| Unit of measurement | percent |
| Rationale | This is indicator is corresponds to SDG 3.b.3 and a detailed rational can be found in its metadata. Access to medicines in general is an integral part of the Universal Health Coverage movement and indispensable to the delivery of quality health care. Measuring and monitoring access to medicines is integral to understanding whether essential medicines are available and affordable. This indicator combines both dimensions into a single evaluation. |
| Preferred data sources | as reported to the SDG monitoring (data collection through Health Action International Project supported by the WHO, The Service Availability and Readiness Assessment survey or the WHO Medicines Price and Availability Monitoring mobile application) |
| Disaggregation | as reported to the SDG; the calculation proposed for the SDG 3.b.3 allows for the following disaggregation:   - public/private facilities - geography – rural/urban areas - therapeutic group - facility type (pharmacy/hospital) - medicine |
| Limitations | The 28 medicines identified for the SDG indicator cover tracers conditions relevant to the PHC-IMPACT (non-communicable diseases, mental health conditions, palliative care and anti-infective) as well as mother and child health, and antiretroviral, therefore a disaggregation by therapeutic group, if available, should be reported.  For further limitations to this indicator please refer to the metadata of SDG 3.b.3 [115]. |

|  | |
| --- | --- |
| Domain | Outputs |
| Subdomain | Access to primary care services |
| Feature | Acceptability |
| **Indicator/question title** | **Patient reported acceptability of primary care services (acc3q120)** |
| Indicator/question definition or question | *No indicator identified. Flagged for further development.* |
| Numerator/denominator or answer choices | To be confirmed. |
| Unit of measurement | To be confirmed. |
| Rationale | In the Tanahashi model, acceptability is defined as the capacity of health services to be appealing and sought by people. It includes factors related to culture, beliefs, religion, gender, confidentiality, and age-appropriateness as well as perceptions related to the value of health services. It is influenced by people's perceptions, previous experiences and interactions with the health system, and expectations. Systematic barriers arise from health personnel's discriminatory attitudes towards certain groups. Health workforce characteristics and ability (e.g. sex, language, culture, age, etc.) to treat all with dignity, create trust and promote demand for services [171]. This indicator captures people’s willingness to seek services. Acceptability is low when patients perceive services to be ineffective or when social and cultural factors such as language or the age, sex, ethnicity or religion of the health professional discourage them from seeking services [169]. All European countries endorse equity of access to health services for all people as an important policy objective. One method of gauging to what extent this objective is achieved is through assessing reports of unmet needs for health care. The problems that people report in obtaining care when they are ill often reflect significant barriers to care [170]. |
| Preferred data sources | - survey – population |
| Disaggregation | none specified |
| Limitations | none specified |

| Domain | Outputs |
| --- | --- |
| Subdomain | Responsiveness of primary care |
| Feature | Resolving capacity of primary care |
| **Indicator/question title** | **Composite measure (res1q121)** |
| Indicator/question definition or question | *Suggested to use a composite measure. Indicator construction flagged for further development.* |
| Numerator/denominator or answer choices | To be confirmed. |
| Unit of measurement | To be confirmed. |
| Rationale | To be confirmed. |
| Preferred data sources | - Analysis of responses across indicators. |
| Disaggregation | none specified |
| Limitations | none specified |

| Domain | Outputs |
| --- | --- |
| Subdomain | Safety of primary care |
| Feature | Medical errors |
| **Indicator/question title** | **Correct diagnosis (saf1q122)** |
| Indicator/question definition or question | Percent of population with cardiovascular disease risk estimated correctly |
| Numerator/denominator or answer choices | Exact percent from survey analysis  Alternate answer choices if exact data is not available:   - 70% or more - more than 50% but less than 70% - 10% to 50% - less than 10% |
| Unit of measurement | percent or category |
| Rationale | It is recommended that therapeutic decisions should be based on cardiovascular risk, however there is evidence that risk is often estimate inaccurately even when guidelines are followed. generalist medical practitioners and specialist medical practitioners tend to underestimate the cardiovascular risk in daily clinical practice, mainly in very high-risk individuals [172], [173]. This indicator would help isolate issues related to medical errors that lead to poor health outcomes. |
| Preferred data sources | existing assessments  expert consensus |
| Disaggregation | none specified |
| Limitations | none specified |
|  |  |
| Domain | Outputs |
| Subdomain | Safety of primary care |
| Feature | Medical errors |
| **Indicator/question title** | **Incident reporting (saf1q123)** |
| Indicator/question definition or question | How many incidents were reported in primary care (audit data)? |
| Numerator/denominator or answer choices | Average number of incidents reported per facility per month |
| Unit of measurement | number of incidents |
| Rationale | Reporting is crucial to reducing the incidence of medical errors even in cases where no harm had occurred to patients since it leads to positive changes in overall care [155]. The World Health Report 2010 identified 10 leading sources of inefficiency in the use of key health service resources. This indicator helps assess inefficiencies of health care services in terms of medical errors and suboptimal quality of care [174]. |
| Preferred data sources | existing assessments  quality inspections  expert consensus |
| Disaggregation | none specified |
| Limitations | none specified |
|  |  |
| Domain | Outputs |
| Subdomain | Safety of primary care |
| Feature | Medicine safety |
| **Indicator/question title** | **Prescription safeguards (saf2q125)** |
| Indicator/question definition or question | Percent of primary care facilities with a protocol in place to ensure that a current medication and problem list is recorded in the health records (e.g. interactions, allergies, etc.) |
| Numerator/denominator or answer choices | Exact percent from survey analysis.  Alternate answer choices if exact data is not available:  70% or more  more than 50% but less than 70%  10% to 50%  less than 10% |
| Unit of measurement | percent |
| Rationale | Patients’ problem and medication lists support continuity of care between health professionals. Properly updated problem and medication lists facilitate the prevention of errors [172]. |
| Preferred data sources | survey – facility  expert consensus |
| Disaggregation | none specified |
| Limitations | none specified |

| Domain | Outputs |
| --- | --- |
| Subdomain | Safety of primary care |
| Feature | Medicine review and reconciliation |
| Indicator/question title | Overall volume of antibiotics prescribed (saf2q127) |
| Indicator/question definition or question | a. Defined Daily Dose of antibiotics per 1,000 population per day (all ATC J01 prescriptions) in primary care |
| Numerator/Denominator or answer choices | as reported by the WHO AMC Network and the OECD Health Care Quality Indicator database  **Numerator:** sum of DDDs ATC J01 prescriptions in the primary care prescription database for the reference year x 1000  **Denominator:** 365 x number of people covered by the database as of 1 January of the reference year |
| Unit of measurement | DDDs per 1,000 population per day |
| Indicator/question definition or question | b. Relative use of quinolones and cephalosporin with respect to total consumption of systemic antibiotics |
| Numerator/Denominator or answer choices | as reported by the WHO AMC Network and the OECD Health Care Quality Indicator database  **Numerator:** sum of DDDs of only ATC J01D and J01M prescriptions in the primary care prescription database for the reference year x 1000  **Denominator:** sum of all DDDs ATC J01 prescriptions in the primary care prescription database in the reference year |
| Unit of measurement | ratio |
| Rationale | Excessive antibacterial consumption leads to wasted financial resources and contributes to the development of antimicrobial resistance. Antibiotics should be prescribed only when there is an evidence-based need, to reduce the risk of resistant strains.  The use of second-line antibiotics (e.g. quinolones and cephalosporin) should be restricted to ensure availability of effective second-line therapy should first-line antibiotics fail. Their volume as a percent of the total volume of antibiotics prescribed has been validated as a marker of quality in the primary care setting [175]. |
| Preferred data sources | WHO AMC Network data 2011 to 2014– estimates on consumption [176]  OECD Health Care Quality Indicators  health information system |
| Disaggregation | N/A |
| Limitations | Data on DDD of antibiotics is available in OECD Health Care Quality Indicators which refers to primary care only. If data is not available on prescription, estimates on consumption are available from WHO AMC Network data – but this does not link to primary care exclusively. WHO AMC data is based on import records, while OECD Health Care Quality Indicators are based on prescribing databases. |

| Domain | Outputs |
| --- | --- |
| Subdomain | Safety of primary care |
| Feature | Medicine safety |
| **Indicator/question title** | **Medication review (saf3q128)** |
| Indicator/question definition or question | Are the following medication review practices implemented in primary care?   - pharmacists actively medically review prescriptions (select one) - members of the primary care team (e.g. primary care practitioner or nurse) actively performs medication reconciliation of patients (e.g. after hospital discharge) (select one) |
| Numerator/denominator or answer choices | yes  no  do not know |
| Unit of measurement | N/A |
| Rationale | Medication review is a process of patients` medicines evaluation to improve the health outcomes and mitigate the drug-related problems. A systematic review of 38 studies of primary care interventions designed to reduce medication related adverse events found that most successful interventions included a medication review conducted by a pharmacist or other clinicians, or focused on multicomponent interventions, which had a medication review by a primary care professional as one component. Studies showed that pharmacist-led medication reviews reduced hospital admissions [177]. |
| Preferred data sources | - key informant |
| Disaggregation | none specified |
| Limitations | None specified. |

| Domain | Outputs |
| --- | --- |
| Subdomain | Effectiveness of primary care services |
| Feature | Effective management and control of diseases |
| **Indicator/question title** | **Control of blood pressure among people treated for hypertension (eff1q129)** |
| Indicator/question definition or question | Percent of population registered for hypertensive treatment who had controlled blood pressure 6 months after treatment initiation |
| Numerator/denominator or answer choices | **Numerator:** number of individuals in the denominator with controlled blood pressure (SBP <140 and DBP <90 mmHg) at the last clinical visit in the most recent quarter (just before the reporting quarter)  **Denominator:** number of individuals newly registered for treatment of hypertension during the quarter than ended 6 months previously  Alternate answer choices if exact data is not available:   - 70% or more - more than 50% but less than 70% - 10% to 50% - less than 10% - do not know |
| Unit of measurement | percent or category |
| Rationale | This indicator is part of the Systems for monitoring of the HEARTS Technical package for cardiovascular disease management in primary health care. Its purpose is to measure the effectiveness of clinical series in the programme to control blood pressure among cohorts of treated individuals [131].  Hypertension is a common disorder and has substantial effects on morbidity and mortality, but adequate treatment has been shown to prevent long-term complications. Hypertension alone is symptomless and can only be discovered if it is measured, but it is an important risk factor for cardiovascular diseases, both ischaemic heart disease and cerebrovascular disease. This indicator can be used to understand if the primary care network is functioning effectively to ensure early detection of disease. If more than 60% of estimated cases with high blood pressure are identified in primary care the coverage of individual services for cardiovascular disease in terms of detection and management of hypertension can be deemed extensive [114]. These indicators contribute to the population-based approach to evaluation of the effectiveness of hypertension management which requires distinction of ‘awareness’ (the proportion of all patients with hypertension report to have a medical diagnosis of hypertension), ‘treatment’ (the proportion of patients with hypertension reporting receiving blood pressure-lowering medication) and ‘control’ (the proportion of patients with hypertension having an average blood pressure reading under the limits) [178]. |
| Preferred data sources | - health information system - register of hypertension patients - expert consensus |
| Disaggregation | none specified |
| Limitations | none specified |
|  |  |
| Domain | Outputs |
| Subdomain | Effectiveness of primary care services |
| Feature | Effective management and control of diseases |
| **Indicator/question title** | **Control of blood glucose among people treated for diabetes (eff1q130)** |
| Indicator/question definition or question | Percent of individuals registered for diabetic treatment whose blood glucose is controlled 6 months after treatment initiation |
| Numerator/denominator or answer choices | **Numerator:** number of individuals in the denominator with blood glucose control (HbA1C measurement <7 mg %) at the last clinical visit in the most recent quarter (just before the reporting quarter)  **Denominator:** number of individuals registered for treatment of diabetes during the quarter that ended 6 months previously  Alternate answer choices if exact data is not available:   - 70% or more - more than 50% but less than 70% - 10% to 50% - less than 10% - do not know |
| Unit of measurement | percent or category |
| Rationale | Diabetes is an ambulatory care sensitive condition. The provision of a wide range of services provided in primary care is associated with better health outcomes at lower costs. The management of registered diabetic patients’ blood glucose over an extended period of time is a reflection of the effectiveness of follow-up services provided by primary health care. |
| Preferred data sources | - health information system - register for diabetes - expert consensus |
| Disaggregation | none specified |
| Limitations | none specified |
|  |  |
| Domain | Outputs |
| Subdomain | Effectiveness of primary care services |
| Feature | Effective management and control of diseases |
| **Indicator/question title** | **Tuberculosis detection and treatment (eff1q131)** |
| Indicator/question definition or question | a. Case detection as percent of tuberculosis cases detected (diagnosed and reported to the national health authority) among the total number of tuberculosis cases estimated to occur countrywide during a 12-months period |
| Numerator/denominator or answer choices | **Numerator:** total number of notified tuberculosis cases  **Denominator:** total number of estimated tuberculosis cases |
| Unit of measurement | percent |
| Indicator/question definition or question | b. Notification rate as number of all new tuberculosis and relapses notified in the reporting period per 100,000 population |
| Numerator/denominator or answer choices | as reported in WHO TB database |
| Unit of measurement | rate |
| Indicator/question definition or question | c. Tuberculosis treatment success rate - percentage of a cohort of tuberculosis cases registered in a specified period that successfully completed treatment with outcomes “cured” and “treatment completed” |
| Numerator/denominator or answer choices | **Numerator:** tuberculosis cases registered in a specified period that were successfully treated during the reference period  **Denominator:** total number of tuberculosis cases registered in the reference period |
| Unit of measurement | percent |
| Rationale | Case detection measures the national tuberculosis program’s integration in the health system, and its ability to diagnose and notify tuberculosis cases. The target is 90% and more. Notification coverage measures the under-notification of detected by laboratory network. In low resources settings and with weak tuberculosis governance some detected tuberculosis patients are not notified by the national tuberculosis program. A stronger interoperable link between laboratory network, private and public mixes heath care providers should be established to exclude under-notification. Coverage should be 95% or more. Notification rate indirectly measures trend of the tuberculosis epidemic. Monitoring of this indicator over time may indirectly indicate the impact of the programme intervention to tuberculosis epidemic. In low resource settings, a substantial investment in health system strengthening (tuberculosis diagnosis, integration in primary care, communication campaign, intensified active tuberculosis case finding in risk groups) may result on the increasing notification rate. This trend will stabilize and then decrease in a short time (2-3 years).  High-quality tuberculosis care is essential to prevent suffering and death from tuberculosis and to cut transmission. This indicator measures a program’s capacity to retain patients through a complete course of tuberculosis treatment regimens with a favourable clinical result. It is an outcome indicator, and it is noteworthy because it is the only outcome indicator that can (and should) be used at all levels. There is a direct and immediate link between this outcome of treatment success and the impact of reduced tuberculosis mortality. |
| Preferred data sources | - data reported in WHO Global tuberculosis report 2017 - for a: tuberculosis reporting system, WHO estimates from <http://www.who.int/tb/country/data/profiles/en/> - for b: laboratory register or other relevant patient management primary records (patient card) or the basic medical unit register or national tuberculosis database - for d: Global Health Observatory data http://apps.who.int/gho/data/view.main.57200 |
| Disaggregation | - all new tuberculosis and relapses - sex - age groups (e.g.<15 yours, >65 years) - HIV-status - rifampicin/multidrug resistant tuberculosis |
| Limitations | The quality of this indicator is affected by many tuberculosis cases with treatment outcome "not evaluated". |

| Domain | Outputs |
| --- | --- |
| Subdomain | Effectiveness of primary care services |
| Feature | Effective management and control of diseases |
| **Indicator/question title** | Cancer survival rates (eff2q155) |
| Indicator/question definition or question | Age-standardised 5-year net survival for adults diagnosed with:   - breast cancer - cervical cancer - colon cancer - rectal cancer |
| Numerator/denominator or answer choices | As reported by the CONCORD-3 study [61] |
| Unit of measurement | percent with 95% CI |
| Rationale | Cancer survival rate enables a comparison of the effectiveness of health systems [54]. Analysing survival following diagnosis can link the efforts put in place to strengthen health systems in terms of effective and timely diagnoses and referrals from primary care with reductions in cancer mortality [179]. |
| Preferred data sources | CONCORD-3 study |
| Disaggregation | none specified |
| Limitations | none specified |

| Domain | Health system outcomes |
| --- | --- |
| Subdomain | Quality |
| Feature | Quality of care for chronic conditions |
| **Indicator/question title** | **Hospital admissions for chronic conditions (qly1q133)** |
| Indicator/question definition or question | Age-standardized acute care hospitalisation rate for conditions where appropriate ambulatory care may prevent or reduce the need for admission to hospital, per 100,000:   - cardiovascular diseases: hypertension - diabetes - respiratory – chronic obstructive pulmonary disease - respiratory - asthma |
| Numerator/denominator or answer choices | **Numerator:** number of hospitalisations with a diagnosis of (exclusions: individual died before discharge):   - hypertension (ICD-10 I10, I119, I129, I139) - diabetes (ICD-10 codes: E10-E14) - chronic obstructive pulmonary disease (ICD-10 J40 with secondary diagnosis J41, J43, J44, J47; J410, J411, J418, J42, J430-432, J438-441, J448-449, J47) - asthma (ICD-10 J450, J451, J458, J459, J46 excluding diagnosis codes cystic fibrosis and anomalies of the respiratory system)   **Denominator:** population age 15+, for the same calendar year x 100,000 (age adjusted). |
| Unit of measurement | age group 15+; age-sex standardized rate per 100,000 population per year |
| Rationale | Asthma, chronic obstructive pulmonary disease, congestive heart failure, and diabetes are four widely prevalent long-term conditions. Common to all these conditions is the fact that the evidence base for effective treatment is well established and much of it can be delivered at the primary care level. A high-performing primary care system can reduce acute deterioration in people living with asthma, chronic obstructive pulmonary disease or congestive heart failure and prevent their admission to hospital [170]. |
| Preferred data sources | - OECD Health Care Quality Indicators - health information system |
| Disaggregation | gender |
| Limitations | none specified |
|  |  |
| Domain | Health system outcomes |
| Subdomain | Quality |
| Feature | Quality of care for chronic conditions |
| **Indicator/question title** | **Avoidable complications (qly1q134)** |
| Indicator/question definition or question | a. Percent of population, age 15+, with established diabetes mellitus who had a major lower extremity amputation |
| Numerator/denominator or answer choices | **Numerator:** number of admissions with a procedure code of major lower extremity amputation and a diagnosis code of diabetes in any field in a specified year  **Denominator:** estimated population with diabetes, age 15+ |
| Unit of measurement | percent or category |
| Indicator/question definition or question | b. Percent of population, age 15+, who had a major lower extremity amputation |
| Numerator/denominator or answer choices | **Numerator:** number of admissions with a procedure code of major lower extremity amputation in a specified year  **Denominator:** total population, age 15+ |
| Unit of measurement | percent |
| Rationale | Poor control of the level of glucose in the blood over the short term can lead to vomiting, dehydration and even cause coma, whereas sustained high levels of blood glucose over several years can result in serious diseases with ongoing consequences for a person's health and wellbeing. For example, diabetes can cause nerve damage and poor blood circulation over time [105]. |
| Preferred data sources | - OECD Health Care Quality Indicators - health information system |
| Disaggregation | gender |
| Limitations | none specified |
|  |  |
| Domain | Health system outcomes |
| Subdomain | Quality |
| Feature | Quality of care for chronic conditions |
| **Indicator/question title** | **Notified tuberculosis cases lost to follow-up (qly1q135)** |
| Indicator/question definition or question | Percent of all tuberculosis cases registered in a specified period that were lost to follow-up treatment for more than 2 consecutive months |
| Numerator/denominator or answer choices | **Numerator:** number of tuberculosis cases registered in a specified period who did not start treatment or whose treatment was interrupted for 2 consecutive months or more  **Denominator:** total number of tuberculosis cases that were notified in the reporting period |
| Unit of measurement | percent |
| Rationale | This indicator is part of the Roadmap to prevent and combat drug-resistant tuberculosis [180], and the Companion handbook to the WHO guidelines for the programmatic management of drug-resistant tuberculosis [180]. WHO recommends tuberculosis treatment is given under direct and supportive observation [181] for tuberculosis treatment success. Currently WHO defines DOT as any person observing the patient taking medications in real-time. Direct treatment observer does not need to be a health professional. If effectively integrated into primary care they can make a significant contribution to the reduction of percentage tuberculosis patients who are lost to follow-up. The target for this indicator is 5% and less. Loss to follow-up may decrease when engaging communities and civil societies in supporting health professionals/health associate professionals to patient/people needs oriented tuberculosis care delivery. |
| Preferred data sources | - data reported in WHO Global tuberculosis report 2017 |
| Disaggregation | By 5 main cohorts:   - new and relapse cases - other retreatments - multidrug-resistant-tuberculosis (all started treatment with second-line drugs) - tuberculosis/HIV [15] - children under 15 (group 1: 0-4 and group 2, 5-14 years of age) |
| Limitations | none specified |
|  |  |
| Domain | Health system outcomes |
| Subdomain | Quality |
| Feature | Quality of care for chronic conditions |
| **Indicator/question title** | **Stage at diagnosis for cancer (qly2q136)** |
| Indicator/question definition or question | Stage at diagnosis for:   - breast cancer - cervical cancer - colorectal cancer |
| Numerator/denominator or answer choices | **Numerator:** total number of cases from the denominator diagnosed in a certain stage  **Denominator:** total number of respective cancer diagnosed in the 12-month reference period |
| Unit of measurement | stage of cancer (T1-4, N1-3, M1) |
| Rationale | It is a very good indicator of effectiveness of patient pathways across levels of care and overall communication mechanisms across facilities (primary care, labs, 2nd 3rd level). Cancer stage at diagnosis is highly correlated to overall effectiveness of health systems, whereas the cancer screening is developed or not. Stage data is readily available and highly comparable across regions/countries. |
| Preferred data sources | - EUROCARE-05 - cancer registries - health information system - tertiary care level or pathology service level monitoring systems |
| Disaggregation | age, gender |
| Limitations | While being collected in EUROCARE-05, stage diagnosis data may be incomplete and accuracy needs to improve in order to fulfil the role in cancer control [182]. |
|  |  |
| Domain | Health system outcomes |
| Subdomain | Quality |
| Feature | Prescribing in primary care |
| **Indicator/question title** | **Secondary prevention/high-risk control (qly3q138)** |
| Indicator/question definition or question | Percent of eligible individuals (defined as age 40+ years with a 10-year cardiovascular disease risk ≥30%, including those with existing cardiovascular disease) receiving drug therapy and counselling (including glycaemic control) to prevent heart attacks and strokes |
| Numerator/denominator or answer choices | **Numerator:** number of eligible surveyed individuals who are receiving drug therapy and counselling  **Denominator:** total number of eligible survey participants (defined as aged 40+ years with a 10-year cardiovascular risk ≥30%, including those with existing cardiovascular disease)  Alternate answer choices if exact data is not available:   - 70% or more - more than 50% but less than 70% - 10% to 50% - less than 10% - do not know |
| Unit of measurement | percent or category |
| Rationale | This indicator is part of the Systems for monitoring of the Technical package for cardiovascular disease management in primary health care. Its purpose is to measure the population-level CVD-risk management [131].  This is indicator 18 corresponding to target 9 of the NCD Global Monitoring Framework for noncommunicable diseases which will track the implementation of the noncommunicable diseases action plan through monitoring and reporting on the attainment of the global targets in 2015-2020. The 25 indicators and the 9 voluntary global targets of the framework provide overall direction and the action plan provides a road map for reaching the targets [122]. More information specifically on this indicator and methods for calculation is available at <http://www.who.int/nmh/ncd-tools/indicator18/en/> and http://www.who.int/nmh/ncd-tools/target9/en/. |
| Preferred data sources | - WHO STEPwise approach to surveillance survey - population survey - expert consensus |
| Disaggregation | none specified |
| Limitations | This is feasible in settings that have a comprehensive population-based survey with behavioural parameters along with physical and biochemical measurements. |
|  |  |
| Domain | Health system outcomes |
| Subdomain | Quality |
| Feature | Prescribing in primary care |
| **Indicator/question title** | **Tuberculosis and rifampicin/multidrug resistant tuberculosis treatment in primary care (qly3q139)** |
| Indicator/question definition or question | Percent of individuals diagnosed with tuberculosis and rifampicin/multidrug resistant tuberculosis initiating treatment in primary care (at ambulatory facility / specialised outpatient treatment facility) |
| Numerator/denominator or answer choices | **Numerator:** number of patients starting treatment at primary care level (ambulatory/outpatient)  **Denominator:** total number of individuals enrolled in treatment |
| Unit of measurement | percent |
| Rationale | This is a new indicator, integrated in the global tuberculosis data collection system to monitor the universal health coverage. It reflects the people-centred model of tuberculosis care, and monitors its implementation [133]. Target for tuberculosis and multidrug resistant tuberculosis should reflect country epidemiological context and prevalence of social determinates; however, an average target is the following: drug-sensitive tuberculosis = 50%, rifampicin/multidrug resistant tuberculosis = 30%, extensively drug-resistant tuberculosis = none. |
| Preferred data sources | - data reported in WHO Global tuberculosis report 2017 not available for primary care level |
| Disaggregation | tuberculosis and rifampicin/multidrug resistant tuberculosis |
| Limitations | none specified |
|  |  |
| Domain | Health system outcomes |
| Subdomain | Quality |
| Feature | Prescribing in primary care |
| **Indicator/question title** | **Access to palliative care (qly3q140)** |
| Indicator/question definition or question | Access to palliative care assessed by morphine-equivalent consumption of strong opioid analgesics (excluding methadone) per death from cancer |
| Numerator/denominator or answer choices | **Numerator:** total morphine-equivalent consumption of strong opioid analgesics (excluding methadone) in mg for the 12-month reference period  **Denominator:** number of deaths from cancer during the 12-month reference period |
| Unit of measurement | rate |
| Rationale | This is indicator 20 of the NCD Global Monitoring Framework to track the implementation of the noncommunicable diseases action plan through monitoring and reporting on the attainment of the global targets in 2015-2020. The 25 indicators and the 9 voluntary global targets of the framework provide overall direction and the action plan provides a road map for reaching the targets [122]. More information on this indicator including methods to calculate it is available at http://www.who.int/nmh/ncd-tools/indicator20/en/. |
| Preferred data sources | - International Narcotics Control Board, Annual report, statistics for 2015 – table XIVe for the numerator [183] - International Agency for Research on Cancer, WHO – GLOBOCAN – for the denominator |
| Disaggregation | none specified |
| Limitations | Potential limitations include incomplete administrative records and incomplete or unusable death registration data. |

| Domain | Outputs |
| --- | --- |
| Subdomain | Equity |
| Feature | Equitable delivery of primary care services |
| **Indicator/question title** | **Composite measure (eqt1q141)** |
| Indicator/question definition or question | *Suggested use of composite measure. Indicator construction flagged for further development.* |
| Numerator/denominator or answer choices | To be confirmed |
| Unit of measurement | To be confirmed |
| Rationale | To be confirmed |
| Preferred data sources | - To be confirmed |
| Disaggregation | none specified |
| Limitations | none specified |

| Domain | Health system outcomes |
| --- | --- |
| Subdomain | Efficiency |
| Feature | Unnecessary procedures |
| **Indicator/question title** | **Unnecessary duplication of medical tests (efc1q142)** |
| Indicator/question definition or question | Percent of generalist medical practitioners who repeated medical tests because previous results were unavailable |
| Numerator/denominator or answer choices | Exact percent reported in survey analysis  Alternate answer choices if exact data is not available:  70% or more  more than 50% but less than 70%  10% to 50%  less than 10% |
| Unit of measurement | percent |
| Rationale | The inappropriate duplication of medical tests is disruptive to the patient and adds an unnecessary cost burden to the health system. It can also reflect problems with coordination if test results are not available at point of care [184]. |
| Preferred data sources | Commonwealth Fund International Survey of Primary Care Physicians in 10 Nations [36]  survey – health professionals  expert consensus |
| Disaggregation | none specified |
| Limitations | Not reported in surveys in the WHO European Region. |

| Domain | Health outcomes |
| --- | --- |
| Subdomain | Health status and well-being |
| Feature | Burden of disease and risk factors |
| **Indicator/question title** | Risk factors – smoking (hsw1q145) |
| Indicator/question definition or question | Age-standardized prevalence of current tobacco use among people aged 15+ years  Note: tobacco use includes cigarettes, cigars, pipes or any other tobacco products. Current use includes both daily and non-daily or occasional use. |
| Numerator/denominator or answer choices | as reported in the Global Health Observatory data repository, prevalence of smoking any tobacco product. |
| Unit of measurement | percent |
| Rationale | This indicator monitors target 5 of the NCD Global Monitoring Framework for noncommunicable diseases which will track the implementation of the noncommunicable diseases action plan through monitoring and reporting on the attainment of the global targets in 2015-2020. The 25 indicators and the 9 voluntary global targets of the framework provide overall direction and the action plan provides a road map for reaching the targets [122]. More information specifically on this indicator and methods for calculation is available at http://www.who.int/nmh/ncd-tools/target5/en/. Voluntary global targets by 2025 include 30% reduction in the prevalence of current tobacco use in persons aged 15+ years [114]. Smoking is a contributing factor to several chronic disease conditions including respiratory diseases, coronary heart disease, stroke, diabetes, cancers, and other diseases. While it is an entirely avoidable risk factor (and it is the largest avoidable risk factor for health), the levels of smoking are influenced by several social, economic and individual factors. The public and preventive health services have an important role to play in educating and thus dissuading individuals from smoking. In this sense, it is a measure of effectiveness of the primary care and preventive services. |
| Preferred data sources | WHO Global Health Observatory |
| Disaggregation | gender |
| Limitations | Estimates are calculated for 2015, 2020 and 2025.  Standardization is done to the WHO global population. |
|  |  |
| Domain | Health outcomes |
| Subdomain | Health status and well-being |
| Feature | Burden of disease and risk factors |
| **Indicator/question title** | Risk factors – alcohol (hsw1q146) |
| Indicator/question definition or question | Per capita alcohol consumption among people aged 15+ years within a calendar year (litres of pure alcohol) |
| Numerator/denominator or answer choices | as reported in the Global Health Observatory data repository, recorded alcohol per capita consumption |
| Unit of measurement | rate - total, litres/capita (aged 15+ years) |
| Rationale | Alcohol consumption is a causal factor in certain cardiovascular diseases and cancers, among some 200 diseases and injuries. Countries have a responsibility in formulating, implementing, monitoring and evaluating public policies to reduce the harmful use of alcohol, and specifically the health system in implementing screening and intervention programs [185], [186]. Alcohol use is associated with numerous harmful health and social consequences, including an increased risk of a range of noncommunicable diseases: cancers, cardiovascular diseases, etc. [105]. This is indicator 3 monitoring target 2 of the NCD Global Monitoring Framework for noncommunicable diseases which will track the implementation of the noncommunicable diseases action plan through monitoring and reporting on the attainment of the global targets in 2015-2020. The 25 indicators and the 9 voluntary global targets of the framework provide overall direction and the action plan provides a road map for reaching the targets [122]. More information specifically on this indicator and methods for calculation is available at http://www.who.int/nmh/ncd-tools/indicator3/en/ and <http://www.who.int/nmh/ncd-tools/target2/en/> . |
| Preferred data sources | Global Health Observatory |
| Disaggregation | age, gender |
| Limitations | Latest year reported in WHO Global Health Observatory is 2016 as of May 2018 update.  Standardization is done to the WHO global population. |
|  |  |
| Domain | Health outcomes |
| Subdomain | Health status and well-being |
| Feature | Burden of disease and risk factors |
| **Indicator/question title** | Risk factors – overweight and obesity (hsw1q147) |
| Indicator/question definition or question | Age-standardized prevalence in people aged 18+ years of:  overweight (defined as BMI ≥ 25 kg/m2) and  obesity (defined as BMI ≥ 30 kg/m2) |
| Numerator/denominator or answer choices | as reported in the WHO Global Health Observatory data repository |
| Unit of measurement | percent of population, age standardised rate |
| Rationale | This is indicator 14 monitoring target 7 of the NCD Global Monitoring Framework while will track the implementation of the noncommunicable diseases action plan through monitoring and reporting on the attainment of the global targets in 2015-2020. The 25 indicators and the 9 voluntary global targets of the framework provide overall direction and the action plan provides a road map for reaching the targets [122]. More information specifically on this indicator and methods for calculation is available at <http://www.who.int/nmh/ncd-tools/indicator14/en/> and <http://www.who.int/nmh/ncd-tools/target7/en/>. Obese adults are at increased risk of adverse metabolic outcomes including increased blood pressure, cholesterol, triglycerides, and insulin resistance. Subsequently, an increase in BMI exponentially increases the risk of noncommunicable diseases such as coronary heart disease, ischemic stroke and type-2 diabetes mellitus [187]. |
| Preferred data sources | WHO Health for All (Health 2020)  OECD – self-reported and measured  European Health Interview Survey 2015 (2019-forthcoming) |
| Disaggregation | age, gender |
| Limitations | Issues of comparability may arise if data is reported from different secondary sources since WHO Health 2020 data comes from surveys while OECD presents both self-reported and measured data separately.  Standardization is done to the WHO global population. |
|  |  |
| Domain | Health outcomes |
| Subdomain | Health status and well-being |
| Feature | Burden of disease and risk factors |
| **Indicator/question title** | Morbidity (hsw1q148) |
| Indicator/question definition or question | a. Age-standardized estimate prevalence of raised blood pressure among persons aged 18+ years (SBP ≥ 140 or DBP ≥90 mmHG) |
| Numerator/denominator or answer choices | as reported in the Global Health Observatory  Input data and methods are described in the NCD-RisC analysis [188] |
| Unit of measurement | percent |
| Indicator/question definition or question | b. Age-standardized estimate prevalence of raised blood glucose/diabetes among persons aged 18+ years (defined as fasting plasma glucose concentrations >=7.0 mmol/l (126mg/dl) or history of diagnosis with diabetes or use of insulin or oral hypoglycaemic drugs) |
| Numerator/denominator or answer choices | as reported in the Global Health Observatory  Input data and methods are described in the NCD-RisC analysis [188, 189] |
| Unit of measurement | percent |
| Rationale | These are indicators 11 and 12 corresponding to monitoring targets 6 and 7 of the NCD Global Monitoring Framework which will track the implementation of the noncommunicable diseases action plan through monitoring and reporting on the attainment of the global targets in 2015-2020. The 25 indicators and the 9 voluntary global targets of the framework provide overall direction and the action plan provides a road map for reaching the targets [122]. More information specifically on these indicator and methods for calculation is available at http://www.who.int/nmh/ncd-tools/en/. |
| Preferred data sources | WHO Global Health Observatory  NCD-RisC |
| Disaggregation | age, gender, socioeconomic status |
| Limitations | Latest available data in the Global Health Observatory is for 2014 for blood glucose and 2015 for raised blood pressure.  Standardization is done to the WHO global population. |
|  |  |
| Domain | Health outcomes |
| Subdomain | Health status and well-being |
| Feature | Burden of disease and risk factors |
| **Indicator/question title** | Disability adjusted life years (hsw1q149) |
| Indicator/question definition or question | Disability adjusted life years per 100,000 population  hypertensive heart disease  diabetes type 2  breast cancer  cervical cancer  colorectal cancer  chronic obstructive pulmonary disease  asthma  tuberculosis  depressive disorder  self-harm |
| Numerator/denominator or answer choices | estimated by WHO reported in the Global Health Estimates |
| Unit of measurement | years per 100,000 population |
| Rationale | The Disability Adjusted Life Year or DALY is a health gap measure that extends the concept of potential years of life lost due to premature death (PYLL) to include equivalent years of ‘healthy’ life lost by being in a state of poor health or disability (1). DALYs for a disease or health condition are calculated as the sum of the years of life lost due to premature mortality (YLL) in the population and the years lost due to disability (YLD) for incident cases of the health condition [187]. |
| Preferred data sources | WHO Global Health Estimates |
| Disaggregation | gender |
| Limitations | Standardization is done to the WHO global population. |
|  |  |
| Domain | Health outcomes |
| Subdomain | Health status and well-being |
| Feature | Mortality |
| **Indicator/question title** | Standardized death rates (hsw2q150) |
| Indicator/question definition or question | Estimated standardized death rates per 100,000 population from the following diseases:  hypertensive heart disease  diabetes type 2  breast cancer  cervical cancer  colorectal cancer  chronic obstructive pulmonary disease  asthma  tuberculosis  self-harm |
| Numerator/denominator or answer choices | reported in the WHO Global Health Estimates |
| Unit of measurement | deaths per 100,000 population |
| Rationale | A death is amenable if, in the light of medical and technology at the time of death, all or most deaths from that cause could be avoided through good quality health care [190]. Measuring the level of amenable mortality rates should provide insights into the quality of service delivery. |
| Preferred data sources | WHO Global Health Estimates |
| Disaggregation | gender |
| Limitations | There is not consensus on the exact causes of amenable mortality, and these causes may change over time as new medical interventions become available. Some studies have also indicated a weak and inconsistent link between amenable mortality and indicators of health services delivery. Standardization is done to the WHO global population |
|  |  |
| Domain | Health outcomes |
| Subdomain | Health status and well-being |
| Feature | Mortality |
| **Indicator/question title** | Premature mortality (hsw2q152) |
| Indicator/question definition or question | Age-standardized overall premature mortality rate from 30-69 years for four major non-communicable diseases (cardiovascular diseases, cancer, diabetes and chronic respiratory diseases) |
| Numerator/denominator or answer choices | as reported in the Health 2020 dataset |
| Unit of measurement | percent |
| Rationale | This indicator is part of the joint monitoring framework for Health 2020, the Sustainable Development Goals and NCD indicators to facilitate reporting in Members States and to enable a consistent and timely way for measuring progress [191]. This is indicator 1 corresponding to target 1 of the NCD Global Monitoring Framework to track progress towards global targets between 2015-2020. The 25 indicators and the 9 voluntary global targets of the framework provide overall direction and the action plan provides a roadmap for reaching the targets [122]. More information specifically on this indicator and methods for calculation is available at <http://www.who.int/nmh/ncd-tools/indicator1/en/> and http://www.who.int/nmh/ncd-tools/target1/en/. |
| Preferred data sources | WHO Health for All: Health 2020 indicators [65] |
| Disaggregation | gender |
| Limitations | Standardization is done to the WHO European population |
