# Supplementary material for: Creating performance intelligence for primary health care strengthening in Europe
Source: BMC Health Serv Res. 2019 Dec 27;19:1006. doi: 10.1186/s12913-019-4853-z (PMC6935208; doi:10.1186/s12913-019-4853-z)
Supplement: Supplementary file 4 — Additional file 4. Glossary of terms [file 12913_2019_4853_MOESM4_ESM.docx]

**Supplementary file 4: Glossary of terms**

This glossary of terms defines all underlined words found in Supplementary file 3 – Indicator passports. The definitions draws from existing international standards, including the System of Health Accounts 2011, chapter on classification of health care providers and existing glossaries, namely: the glossary of terms of the WHO European Framework for Action on Integrated Health Services Delivery; the WHO systems strengthening glossary; other glossaries developed by WHO technical units. The terms are organized in three sections, alphabetical order by section: health workforce; settings of health services delivery; and other, general terms.

**Health workforce terms**

**Allied health professionals** are diverse group of health care professionals who provide necessary services to patients in addition to, or in place of, services provided by physicians, nurses and paramedical practitioners [192].

**Carers/family carers** refer to individuals who provide unpaid care for a member or members of their family, friends or community [134]. They can be any relative (spouse, adult children, daughters- and sons-in-law), friend or neighbour who provides of a broad range of assistance with personal care or basic activities of daily living to people with functional limitations. They may provide regular, occasional or routine care, that can either be 'hands-on' or they can be involved in organizing care delivered by others, sometimes even at distance. They can live with, or separately from the person receiving care. This is in contrast with a provider associated with a formal service system, whether paid or on a volunteer-basis (formal caregiver) [193], [194].

**Dentist** refers to a health professional that diagnoses, treats and prevents diseases, injuries and abnormalities of the teeth, mouth, jaws and associate tissues by applying the principles and procedures of modern dentistry. Occupations included in this category require completion of university-level training in theoretical and practical dentistry or a related field [195].

**Dieticians and nutritionists** (ISCO-08 2265) are health professionals who assess, plan and implement programmes to enhance the impact of food and nutrition on human health. Part of this subgroup are clinical dieticians, nutritionists, public health nutritionists, etc. [195].

**District therapeutists** (part of ISCO-08 2211) are a type of **generalist medical practitioners** often found in countries of the Commonwealth of Independent States.

**District paediatric doctors** (part of ISCO-08 2211) are a type of **generalist medical practitioners** often found in countries of the Commonwealth of Independent states

**Feldscher** (part of ISCO-08 2240) are a type of **paramedical practitioners**.

**General medical practitioners/family medicine doctors/primary care doctors** (part of ISCO-08 2211) are a type of **generalist medical practitioners.**

**Generalist medical practitioners** (ISCO-08 2211) are physicians including family and primary care doctors, who do not limit their practice to certain disease categories or methods of treatment and may assume responsibility for the provision of continuing and comprehensive medical care to individuals, families and communities [192], [196]. Occupations included in this category require completion of a university-level degree in basic medical education plus postgraduate clinical training or equivalent for competent performance. Medical interns who have completed their university education in basic medical education and are undertaking postgraduate clinical training are included here. Although in some countries ‘general practice’ and 'family medicine' may be considered as medical specializations, these occupations should always be classified here [195]. In Commonwealth of Independent States, **district paediatric doctors** and **district** **therapeutists** are included in this category.

**Health associate professionals** (ISCO-08 32) are part of the wider occupational group of Technicians and Associate Professionals. They support the diagnosis and treatment of illness, disease, injuries and impairments; as well as the implementation of health care plans typically established by medical, nursing and other health professionals. The types of tasks usually performed by health associate professionals include: testing and operating medical imaging equipment; administering radiation therapy; performing clinical tests on specimens of bodily fluids and tissues; preparing medications and other pharmaceutical compounds under the guidance of pharmacists; designing, fitting, servicing and repairing medical and dental devices and appliances; providing nursing and personal care and midwifery support services; and using herbal and other therapies [197]. This category includes medical and pharmaceutical technicians (ISCO-08 321), nursing and midwifery associate professionals (ISCO-08 322).

**Health professionals** (ISCO-08 22) are professionals who establish and undertake research and develop and apply scientific knowledge in a range of health and related fields including: medicine, complementary medicine, dentistry, optometry, environmental health and occupational health. Specific occupations within the health professionals group include: physicians, nursing and midwifery professionals; paramedics; opticians; dentists; speech therapists; dieticians; psychiatrists, and; other health professionals. The tasks undertaken by health professionals involve: conducting research and obtaining scientific knowledge through the study of human and animal disorders; diagnosing illnesses and ways of treating them; the planning, management and evaluation of the care of patients; advising on or dispensing and applying preventive and curative measures; promoting health; and, preparing scientific papers and reports [198]. This category includes medical doctors (ISCO-08 221), nursing and midwifery professionals (ISCO-08 222), paramedical professionals (ISCO-08 224).

**Midwife (professionals)** (ISCO-08 2222) plan, manage, provide and evaluate midwifery care services before, during and after pregnancy and childbirth. They provide delivery care for reducing health risks to women and new-born children, working autonomously or in teams with other health care providers.

**Midwife (associate professionals)** (ISCO-08 3222) implement care, treatment and referral plans already established by medical, midwifery and other health professionals.

**Narrow specialists** are specialized physicians in countries of the Commonwealth of Independent States and Eastern Europe often working in polyclinics and outpatient departments of hospitals. They usually have less clinical training than specialist medical practitioners, and instead a brief training course for initial specialization. They primarily provide medical treatment, while the generalist medical practitioner performs the follow-up with the patient [199].

**Nurses (professionals)** (ISCO-08 2221) provide treatment, support and care services for people who are in need of nursing care due to the effects of ageing, injury, illness or other physical or mental impairment, or potential risks to health. They assume responsibility for the planning and management of care of patients, including the supervision of other health care workers, working autonomously or in teams with medical doctors and others in the practical application of preventive and curative measures [195]. Nurses (professionals) include **general practice nurses, district nurse, specialist nurse and nurse practitioners.**

**Nurses (associate professionals)** (ISCO-08 3221) provide basic nursing and personal care for people in need of such care due to effects of ageing, illness, injury or other physical or mental impairment. They generally work under the supervision of, and in support of, implementation of health care, treatment and referral plans established by medical, nursing and other health professionals. The distinctions between nursing and midwifery professionals and associate professionals should be made on the basis of the nature of the work performed in relation to the tasks specified in this definition and in the relevant unit group definitions. The qualifications held by individuals or that predominate in the country are not the main factor in making this distinction, as training arrangements for nurses and midwives vary widely between countries and have varied over time within countries [195].

**Nurse practitioner/advanced practice nurses** are nurses (professionals) (part of ISCO-08 2221) who have acquired the expert knowledge base, complex decision-making skills and clinical competencies for expanded practice, the characteristics of which are shaped by the context and/or country in which s/he has the credentials to practise. A master's degree is recommended for entry level [192], [200].

**Occupational therapists** (part of ISCO-08 2269) are health professionals who provide diagnostic, preventive, curative and rehabilitative health services. Among other tasks they can develop and implement treatment plans for injuries, illnesses and other physical and mental impairments; can administer therapeutic care and treatment to patients; can recommend environmental adaptations in the home, leisure, work and school environments on an individual or a group basis to enable individuals with functional limitations to perform their daily activities and occupations [195].

**Paediatricians/district paediatricians/district paediatric doctors** are considered **generalist medical practitioner** (ISCO-08 2211) only when they are the first point of contact for children, for example in countries of the Commonwealth of Independent States. In other cases when they consult as a specialist, they are considered specialist medical practitioners (ISCO-08 2212).

**Paramedical practitioners** (ISCO-08 2240) are health professionals who provide advisory, diagnostic, curative and preventive medical services more limited in scope and complexity than those carried out by medical doctors. They work autonomously, or with limited supervision of medical doctors, and apply advanced clinical procedures for treating and preventing diseases, injuries and other physical or mental impairments common to specific communities [201]. Examples of occupations: **feldsher**, advanced care paramedic, clinical officer (paramedical), primary care paramedic.

**Pharmacists** (ISCO-08 2262) are health professionals who store, preserve, compound and dispense medicinal products and counsel on the proper use and adverse effects of drugs and medicines following prescriptions issued by medical doctors and other health professionals. This category includes dispensing chemist and retail pharmacists but is to the exclusion of pharmaceutical technician and assistant [195].

**Pharmaceutical technician and assistants** (ISCO-08 3213) are health associate professionals who perform a variety of tasks associate with dispensing medicinal products under the guidance of a pharmacist or other health professional. This category includes dispensing technicians, pharmaceutical assistants, pharmaceutical technicians, etc. [195].

**Physicians/medical doctors** study, diagnose, treat and prevent illness, disease, injury and other physical and mental impairments in humans through the application of the principles and procedures of medicine [202]. Include **generalist medical practitioners**, **specialist medical practitioners** and in countries of the Commonwealth of Independent States **narrow specialists**.

**Physiotherapists** (ISCO-08 2264) assess, plan and implement rehabilitative programmes that improve or restore human motor functions, maximize movement ability, relieve pain syndromes, and treat or prevent physical challenges associated with injuries, diseases and other impairments [195].

**Physiotherapist technicians** (part of ISCO-08 3255) are health associate professionals who provide physical therapeutic treatments to patients n circumstances where functional movement is threated by injury, disease or impairment. Therapies are usually provided according to rehabilitative plans established by a physiotherapists or other health professional [195].

**Practising health professionals** are health professionals who are actively practising medicine in public and private institutions and provide services for individual patients [203]. In case of data not available for practising health professionals, data closest to practising (professionally active health professionals, health professionals with active license) can be used.

**Primary care health professionals** are a sub-group of health professionals who provide services in primary care.

**Public health professionals** (ISCO-08 2212) are specialists working to improve the public health deficits by providing health surveillance through promoting the development of indicator-based comprehensive health monitoring systems; promoting health behaviour and lifestyles, and reducing risk factors; working to help reduce inequity in health; helping to decision-making in health care among different players **[204]**.

**Specialist medical practitioners** are physicians who diagnose, treat and prevent illness, disease, injury and other physical and mental impairments using specialized testing, diagnostic, medical, surgical, physical and psychiatric techniques, through application of the principles and procedures of modern medicine. They plan, supervise and evaluate the implementation of care and treatment plans by other health-care providers. They specialize in certain disease categories, types of patient or methods of treatment, and may conduct medical education and research activities in their chosen areas of specialization [195]. Include specialist medical doctors, specialist doctor, etc. [205].

**Social workers** (part of ISCO-08 2635) provide advice and guidance to individuals, families, groups, communities and organizations in response to social and personal difficulties. They assist clients to develop skills and access resources and support services needed to respond to various issues arising from unemployment, poverty, disability, addiction, etc. [195]

**Speech therapist** (part of ISCO-08 2266) is a health professional who evaluates, manages and treats physical disorders affecting human speech, communication and swallowing. They prescribe corrective devices or rehabilitative therapies for speech disorders and related sensory and neural problems, and provide counselling on communication performance. This category includes language therapists, speech pathologists, speech therapists, etc. [195].

**Settings of services delivery terms**

**Ambulatory multi-profile (specialty) group practices / polyclinics** (HP.3.4.9) comprise establishments that are engaged in providing a wide range of outpatient services, by a medical and paramedical staff, and often support staff too, usually bringing together several specialities and/or serving specific function of primary care and/or secondary care [206].

**Dental practices** (HP.3.2) comprise independent establishments of health professionals who hold a university-level degree in dental medicine or a qualification at a corresponding level and are primarily engaged in the independent practice of general specialised dentistry. These practitioners operate private or group practices in their own offices and either provide comprehensive preventive, reconstructive or emergency care or specialise in a single field of dentistry [206].

**Facilities providing ambulatory health care / providers of ambulatory health care** (HP.3) comprise establishments that are primarily engaged in providing health care services directly to outpatients who do not require inpatient services [206]. This includes both offices of general medical practitioners and medical specialists and establishments specializing in the treatment of day-cases and in the delivery of home care services. Health professionals in ambulatory health care primarily provide services to patients who visit the health professional’s office, or the practitioners visit the patients at home. Consequently, these establishments do not usually provide inpatient services. This item has five subcategories, including: medical practices, dental **practices** (HP.3.2), other health care practitioners, ambulatory health care centres and **providers of home health care services** (HP3.5)

**Networks of primary care facilities** or care groups are collaborative networks that include a large variety of teams and health professionals who together provide a broad integrated set of health services [168].

**Nurses and midwives offices** (e.g. health post) (part of HP.3.3) are providers of ambulatory health care and comprise independent health professionals, in this case nurses and midwives, that operate in their own office without a generalist or specialist physicians [206].

**Offices of general medical practitioner** (HP.3.1.1) comprise establishments of health professionals who hold the degree of a doctor of medicine or a corresponding qualification and are primarily engaged in the independent practice of general/family medicine. Although in some countries “general practice” and “family medicine” may be considered as medical specialisations, these occupations should always be classified here [206]. **Generalist medical practitioner solo practice** refers to offices held by a single practitioner.  **Ambulatory group practice** refers to centres of multiple general medical practitioners.

**Offices of medical specialists** (e.g. practices of independent psychiatrists, offices of psychotherapists, offices of cardiologists, ophthalmologists, ENT, paediatricians of specialised care, etc.) (HP.3.1.2 and HP.3.1.3) comprises establishment of health professionals holding a degree of doctor of medicine with a specialisation mental medicine, a corresponding qualification or a medical doctor with a specialisation other than general medicine (equivalent to ISCO-08 Code 2212) [206].

**Other ambulatory health care centres** (HP.3.4) (e.g. family planning centers, free-standing ambulatory surgery centers, dialysis care centres) comprise establishments that are engaged in providing a wide range of outpatient services by a team of medical and paramedical staff, often along with support staff, that usually bring together several specialities. They differ from offices of medical specialists (HP.3.1.3) by their multi-specialisations, the complexity of the medical-technical equipment used and the range of types of health professionals involved [206].

**Outpatient departments of hospitals** (part of HP.1) (general hospitals providing out-patient, day care services) comprise licensed establishments that are primarily engaged in providing medical, diagnostic and treatment services that include physician, nursing and other health services that provide day care, outpatient and home health care services as secondary activities [206].

**Pharmacies, retailers and other providers of medical goods** (HP.5) refers to specialised establishments whose primary activity is the retail sale of pharmaceuticals and other medical goods to the general public for individual or household consumption or utilisation. Pharmaceuticals include both prescribed and non-prescribed medicines, either manufactured or prepared by onsite pharmacists [206].

**Polyclinics** (HP.3.4.9) see **ambulatory multi-profile (speciality) group practice/polyclinic**

**Practice** is understood as a provider with more than one generalist medical practitioner.

**Primary care facilities** refer to ambulatory care facilities such as primary care centre, office of generalist health professional, ambulatory health care centre, family planning centre, home health care centre, nursing home, and polyclinic; other settings such as walk-in treatment centre, outpatient department of a district/general hospital, ambulance, mobile clinic, laboratory, pharmacy, and palliative care establishment; and, rural-specific facilities such as rural physician ambulatory, feldsher assistance point, midwifery post and rural health house [207].

**Provider** groups together all organization arrangements of health professionals (practice with more than one health professional, solo practices, etc.).

**Providers of ancillary services** (HP.4) include establishments that provide specific ancillary type of services directly to outpatients under the supervision of health professionals and are not included within the episode of treatment by other providers. They include medical and diagnostic laboratories such as diagnostic imaging centres, medical x-ray laboratories, medical pathology laboratories, clinical laboratories [206].

**Providers of preventive care** (HP.6) comprise organisations that primarily provide collective preventing programmes and campaigns/public health programmes for specific groups of individuals or the population-at-large, such as health promotion and protection agencies or public health institutes as well as specialised establishments providing primary preventive care as their principal activity [206].

**Providers of home health care services** (HP.3.5) comprise establishments that are primarily engaged in providing skilled nursing services in patients’ homes, along with a range of the following: personal care services: medical social services, support in medications, use of medical equipment and supplies, counselling; 24-hour home care; occupational and vocational therapy; dietary and nutritional services; speech therapy; audiology; and high-tech care, such as intravenous therapy [206].

**Residential long-term care facilities** (HP.2), also known as high dependency care facilities, are establishments primarily engaged in providing inpatient nursing and rehabilitative services to individuals requiring nursing care [208].

**General terms**

**Accessibility** (of health services) include aspects of health services or health facilities that enhance the ability of people to reach a health professional, in terms of location, time and ease of approach [209], [210].

**Accountability arrangements** make explicit the ways in which actors are expected to perform and interact according to their mandated roles and responsibilities [211].

**Accountability** is defined according to its necessary elements: a clear mandate, with the necessary resources and adequate incentives for its fulfilment, and tended to through regular supervision [212], [211].

**Accreditation (facilities)** is a process by which an authorized body, usually non-governmental organization, assesses and recognizes an organization as achieving pre-determined and published standards, demonstrated through an independent, external, periodic, on-site peer assessment of that organization's level of performance. Accreditation standards are usually regarded as optimal and achievable and are designed to encourage continuous improvement efforts within the accredited organizations. Accreditation is often a voluntary process in which organizations choose to participate [209], [213], [214]. *See also* certification.

**Actors (in health)** are broadly characterized as those individuals, organizations, groups or coalitions that have the capacity to exert influence over policy or decision-making or are mandated with the responsibility to carry out a particular aspect of a given health system function [215].

**Ambulatory care sensitive conditions** are defined as those conditions for which hospitalization can be avoided with timely and effective care in ambulatory settings [212], [211].

**Ambulatory care** comprise those health services provided to patients who are not confined to an institutional bed as inpatients during the time the services are rendered [216]. Ambulatory care includes medical services of general [10] and specialized (secondary) nature. Examples of facilities that provide ambulatory services are: primary care clinics and physician's' offices, hospital-based outpatient clinics, ambulatory surgical centres, public health clinics, imaging centres, ambulatory behavioural health and substance abuse clinics and physical therapy and rehabilitation centres [217].

**Assessment** defines a formal evaluation of a process or system, either quantitative or qualitative [209], [218].

**Budgeting** defines a process of elaborating a detailed plan for the future showing how resources will be acquired and used during a specific time period, expressed in formal, measurable terms [209].

**Capital expenditure** are costs for resources that last more than one year, such a s building, vehicles, computers, pre-service training [209]. *See also* recurrent expenditure.

**Cardiovascular disease risk assessment** refers to a comprehensive risk assessment in adults with no known cardiovascular disease using simple risk-scoring tools. This can help identify those at high risk and initiate early preventive interventions. The level or risk can help guide decisions about whether to initiate preventive interventions and treatment intensity. WHO guidelines are detailed in the HEARTS Technical package published by WHO [219].

**Cardiovascular disease risk stratification** consists of the categorization and management of people according to their likelihood or chance for a cardiovascular event (heart attack or stroke). WHO guidelines are detailed in the HEARTS Technical package published by WHO [219].

**Cardiovascular disease risk prediction charts** are used to determine the level of risk for developing CVD over a defined period (e.g. 10 years). Calculations consider the combined effect of multiple risk factors, including age, gender, smoking status, blood pressure and total cholesterol or body mass index. WHO guidelines are detailed in the HEARTS Technical package published by WHO [219]. *See also* **WHO/ISH cardiovascular risk prediction charts**

**Care coordinator** is a health professional who acts as the key point of contact, from health promotion and disease prevention to targeted referral to specialist care. The care coordinator coordinates patient care throughout the entire continuum of care [209], [220]. *See also* **case manager**.

**Care pathway** or care map refers to an aid (in addition to clinical guideline) that maps the patient pathway through the care system. It plans for the management of patient care that set goals for the patients and provide the sequence of interventions that physicians, nurses and other health professionals should carry out in order to reach the desired goals in a given time period [209], [221]. *See also* clinical guidelines and clinical protocols.

**Care plans** are a personalized record (written and/or electronic form) of the outcome from care planning discussions and decisions taken with the aim to address an individual’s full range of needs [134].

**Case manager** arranges for the provision of continuous care across different services through the integration and coordination of services based on individual needs and system resources. The fundamental difference with a care coordinator is that the case manager not only ensures the continuum of care by focusing on the transition between levels of care, but also ensures the integration and utilization of system resources (e.g. insurance, payment schemes, social care arrangements etc.) [209], [222], [223]. *See also* **care coordinator**.

**Catchment area** refers to a geographic area defined and served by a health programme or facility which is delineated based on population distribution, national geographic boundaries, and transportation accessibility [208].

**Certification of an organization/facility**, or part of an organization/facility, refers to a process by which an authorized body, either governmental or nongovernmental organization, evaluates and recognizes an organization/facility as meeting pre-determined requirements or criteria. It usually implies that the organization/facility has additional services, technology, or capacity beyond those found in similar organizations/facilities [209], [214], [220]. *See also* accreditation and licensure.

**Certification of individual practitioners** refers to a process by which an authorized body, either governmental or nongovernmental organization, evaluates and recognizes the individual as meeting pre-determined requirements or criteria. It implies that the individual has received additional education and training and demonstrated competence in a specialty area beyond the minimum requirements set for licensure [209], [214], [220]. *See also* licensure.

**Clinical practice guidelines** refer to systematically developed, evidence-based recommendations that support the health professionals and patients to make decisions about the most appropriate, efficient care in specific clinical circumstances [209], [212], [224], [208]. *See also* care pathway and clinical protocols.

**Clinical protocols** are defined as an agreed framework outlining the care to be provided to patients according to a type of care, describing why, where, when and by who the care is given [212]. *See also* care pathway and clinical guidelines.

**Co-insurance** is a cost-sharing requirement whereby the insured person pays a share of the cost of the medical service (e.g. 10%) [225].

**Consumer groups** include associations and organizations that represent the rights of consumers and advance their interests.

**Consumer health related group** include consumer groups with a specific focus/special interest on health related activities and topics.

**Consumption-based reimbursement scheme** adjusts the level of reimbursement with the expenses for medicines of a patient within a time period (increasing reimbursement with rising consumption) [50].

**Continuous professional development** refers to learning opportunities during a health professional’s career, ideally designed as inquiry-based, practice-based and problem-based learning opportunities to promote reflection, problem-solving, self-directed learning, and professional responsibility, as well as focused on relevant issues faced by the workforce. It includes continuing medical education, continued professional education and in-service training [212], [94].

**Controlled blood pressure** is defined as a blood pressure of 120mm Hg systolic and a blood pressure of 80mm Hg diastolic. When systolic blood pressure is equal or above 140 mm Hg and/or diastolic blood pressure equal to or above 90 mm Hg the blood pressure is considered to be raised or high [226].

**Coordination** is defined as the extent to which services in a specific episode of care and the provision of services at intervals over time and across the lifespan promote the best results [207], [227].

**Co-payment** is a fixed sum (e.g. $15) or a percentage of the tariff (e.g. 10%) paid by an insured individual for the consumption of itemized health care services (e.g. per hospital day, per prescription item) [225].

**Disease specific reimbursement scheme** determines eligibility and reimbursement rate based on the underlying disease treated. A medicine may be reimbursed at different rates for the treatment of different diseases [50].

**Discharge letter** refers to the form completed by the provider releasing the patient containing information regarding procedures undertaken, diagnosis and treatment.

**Discharge planning** refers to the process by which an admitted inpatient’s needs on discharge are anticipated, planned for or arranged [208].

**Dispensarization** is a method for monitoring the health of selected population groups through screening (case-detection) and systematic treatment and follow-up. It is prevalent in members of the Commonwealth of Independent States.

**District level** refers to a second level administrative division that has jurisdiction over an urban/rural area that can cover several municipalities.

**Domains** capture the dynamics between areas for action to optimally reason and sequence strategic efforts to transform health services delivery. In the European Framework for Action, domain cluster areas for action according to: populations and individuals, services delivery processes, system enablers, and change management [212], [211]. When translated into a monitoring framework of health services delivery, the domains reflect the capacity of primary care in terms of structures and model of care, the performance of primary care in terms of care contact, outputs and health system outcomes, and health outcomes in terms of impact [207].

**Effectiveness** is defined as the extent to which services are delivered in line with the current evidence-based, for the optimal delivery of services for desired outcomes [212], [228]. It measures the extent to which a specific intervention, procedure, regimen or service, when deployed in the field in routine circumstances, does what is intended to do for a specified population [209].

**Efficiency** is the capacity to produce the maximum output for a given input [209].

**Electronic health records** are defined as real-time, patient-centred records that provide immediate and secured information to authorized users and that play a vital role in universal health coverage by supporting the diagnosis and treatment of patients through provision of rapid, comprehensive and timely patient information at the point of care [212], [113].

**Eligibility for reimbursement coverage** are criteria based on which expenses on medicine are fully or partially paid for by a public payer. Four schemes are considered: **product-specific**, **disease-specific**, **population-groups-specific** and **consumption-based** [50].

**Equity** in health refers to the absence of systematic or potentially remediable differences in health status, access to health care and health-enhancing environments, and treatment in one or more aspects of health across population groups defined socially, economically, demographically or geographically within and across countries [209]. Factors weighing on equity and considered in the scope of the tool include gender, age, socioeconomic status and rural–urban classifications.

**Essential medicines list** is developed by the WHO and serves as a guide for the development of national and institutional essential medicine lists. It is updated and revised every two years by the WHO Expert Committee on Selection and Use of Medicines. The latest update, published on 6 June 2017, marks the 40^th^ Anniversary of this flagship WHO tool [229].

**Evaluation** is defined as the systematic and objective assessment of the relevance, adequacy, progress, efficiency, effectiveness and impact of a course of actions, in relation to objectives and taking into account the resources and facilities that have been deployed [209].

**Facility/institution ownership type** is a classification for ownership. There are three types: **publicly owned** facilities owned or controlled by a governmental unit or another public corporation (where control is defined as the ability to determine the general corporate policy); **not-for-profit privately owned** facilities that are legal or social entities created for the purpose of producing goods and services, whose status does not permit them to be a source of income, profit or other financial gain for the unit(s) that establish, control or finance them; and, **for-profit privately owned** facilities that are legal entities set up for the purpose of producing goods and services and are capable of generating a profit or other financial gain for their owners [192].

**Final diagnosis** refers to the confirmation of a diagnosis not just the preliminary diagnosis that requires the patient to visit another physician.

**First contact visit** is considered when the patient is visiting the physician for the first time for the particular health problem and the visit was not by referral. In this case the physician is acting as the entry point into the care system.

**Follow-up consultations/visits** include services offered to manage condition after diagnosis.

**Foot vibration perception by tuning fork** measures the sensitivity to vibration and is important for early diagnosis of diabetic neuropathy and prevention of diabetic foot amputation.

**Formulary** refers to a list of drugs, usually by their generic names, and indications for their use. A formulary is intended to include a sufficient range of medicines to enable medical practitioners to prescribe all medically appropriate treatment for all reasonably common illnesses [208].

**Full-time equivalent** employment is defined as total hours worked divided by average annual hours worked in full-time jobs. Depending on data availability on working hours, full-time equivalent level may also be calculated in the following ways: (i) a worker with a full-time employment contract should be counted as 1 FTE. Concerning workers who do not have a full-time employment contract, full-time equivalent should be measured by the number of hours of work mentioned in each contract divided by the normal number of hours worked in full-time jobs; [61] a worker with a full-time employment contract should be counted as 1 FTE. Concerning workers with part-time contracts, the practice in many countries is simply to consider that 2 part-time workers = 1 FTE [192].

**Gatekeeper** is a primary care health professional who has responsibilities for the provision of primary care as well as for the coordination of specialized care and referral [209] [230].

**General practice/family medicine** is the discipline of medicine for the provision of comprehensive and continuing care to individuals in the context of their family and community. The scope of family medicine encompasses all ages and both sexes. Providers often include generalist medical practitioners, physician’s assistants, family nurses [212], [231].

**HbA1c** is the glycated haemoglobin test used in the diagnosis of diabetes mellitus. WHO-backed expert report recommending the acceptability of this test as an additional test to diagnose diabetes is found here <http://www.who.int/diabetes/publications/diagnosis_diabetes2011/en/>.

**Health information system** provides the underpinnings for decision-making and has four key functions: (i) data generation, [61] compilation, (iii) analysis and synthesis, and [232] communication and use. the health information system collects data from health and other relevant sectors, analyses the data, ensures their overall quality, relevance and timeliness, and converts the data into information for health-related decision-making [192], [106].

**Health insurance** is a contract between the insured and the insurer to the effect that in the event of specified events (determined in the insurance contract) occurring the insurer will pay compensation either to the insured person or the health service provider. There are two major forms of health insurance. One is private health insurance, with premiums based on individual or group risks. the other is social security, whereby in principle society's risks are pooled, with contributions by individuals usually dependent on their capacity to pay [209], [206].

**Health literacy** is defined as the achievement of a certain level of knowledge, personal skills and confidence to take action to improve personal and community health by changing personal lifestyles and living conditions [134], [212].

**HEADS assessment** is a check-list approach which health workers could use to understand the adolescent they are working with. The rapid assessment includes questions that provide information on the psychological and social dimensions of the adolescent’s life including: family life, interests and education performance, eating and exercise habits, hopes for the future, social and recreational activities, whether they smoke or use other psychoactive substances, thoughts and feelings about their sexual activity, and how they feel and whether they have thought of hurting themselves. The acronym stands for Home Education Eating Exercise Ambition Activities Drug-use Sexuality Suicide [233].

**Health needs** refer to objectively determined deficiencies in health that require health services, such as health protection, health promotion, disease prevention, diagnosis, treatment, management, long-term care, rehabilitation and palliative care [209], [212].

**Health needs assessment** refers to a systematic procedure for determining the nature and extent of problems experienced by a specified population that affect their health, either directly or indirectly. Needs assessment makes use of epidemiological, sociodemographic and qualitative methods to describe health problems and their environmental, social, economic and behavioural determinants [208].

**Health professional association** represents the interests of health professionals and specialties by supporting national health policy development, engaging in negotiations on pay and working conditions of members, supporting continuous professional development, developing undergraduate and post-graduate education curricula and/or the development of clinical practice guidelines. This role is distinguished from health professional regulators, representing the interests of patients [94].

**Health promotion** refers to any combination of health education and related organizational, political and economic interventions designed to facilitate behavioural and environmental adaptations that will improve or protect health [208].

**Health service** refers to any service (i.e. not limited to medical or clinical services) aimed at contributing to improved health or to the diagnosis, treatment and rehabilitation of individuals [209].

**Health services delivery processes** are defined as the unique processes inherent to the health services delivery function that contribute to the performance of health services delivery. these processes include: selecting services, designing care, organising providers, managing services and improving performance [212], [116].

**Health technology assessment** is the systematic evaluation of the properties, effects or other impacts of health care technology. It is intended to inform decision-makers about health technologies and may measure the direct or indirect consequences of a given technology or treatment [208].

**Health workforce registry** refers to a national registry with individual data of health workforce. It is meant to provide a count of and information on all health care personnel that either have worked or are currently working at national or sub-national levels, including private sector [103].

**Incentives** refer to rewards reinforcing positive performance and removing barriers that perversely effect desired performance to inspire and motivate health professionals, organizations and patients to work towards defined objectives [212], [234].

**Incident reporting** refers to a quality of care process for reporting undesirable clinical outcomes resulting from some aspect of diagnosis or treatment, and not an underlying disease. It may also be referred to as Critical Incident Report/adverse event reporting [235].

**Integrated health and social care plan** is a dynamic document based on an assessment which outlines the types and frequency of care services that a client receives. It may include strategies, interventions, continued evaluation and actions intended to help a person to achieve or maintain goals [208]. Integrated health and social care plan provides coordination of care across the system’s different functions, activities and operating units. It encompasses horizontal and vertical integration including discharge management and rehabilitation arrangements, a transfer letter to primary/community care services/rehabilitation, etc.. [140], [139].

**Legally recognized groups** refer to organizations/associations that are formalized as a registered agent according to the country’s bylaws of incorporated business or non-profit entities.

**Licensure** defines the process by which a governmental authority grants permission, usually following inspection against minimal statutory standards, to an individual practitioner or healthcare organization to operate or to engage in an occupation or profession. **Licensure to individuals** is usually granted after some form of examination or proof of education and maybe renewed periodically. **Licensure to organizations** is granted following an on-site inspection to determine if minimum health and safety standards have been met [209], [213], [214]. *See also* accreditation and certification.

**Maintenance programme** in terms of medical equipment, can be implemented in a number of ways including establishing service contracts with device manufacturers, independent service organizations, carrying out maintenance activities by employees of the facility, service contractors or other external service providers. A comprehensive maintenance programme includes identifying an inventory, choosing a methodology and allocating financial, physical and human resources to the programme [154].

**Managing facilities** is defined as the process of planning and budgeting, aligning resources, overseeing implementation and monitoring of results to maintain a degree of consistency and order in the delivery of services and act upon observed deviations from plans, problem-solving and troubleshooting as needed [212], [116], [236], [237].

**Medical device** is an article, instrument, apparatus or machine that is used in the prevention, diagnosis or treatment of illness or disease, or for detecting, measuring, restoring, correcting or modifying the structure or function of the body for some health purpose. Typically, the purpose of a medical device is not achieved by pharmacological, immunological or metabolic means [154].

**Medical equipment** is a medical device requiring calibration, maintenance, repair, user training, and decommissioning − activities usually managed by clinical engineers. Medical equipment is used for the specific purposes of diagnosis and treatment of disease or rehabilitation following disease or injury; it can be used either alone or in combination with any accessory, consumable, or other piece of medical equipment. Medical equipment excludes implantable, disposable or single-use medical devices [154].

**mHealth** is defined as the use of mobile technologies to support health information and medical practices, often incorporated into services such as health call centres or emergency number services [212], [113].

**Model of care** is defined as an evolving conception of how services should be delivered. The evolution of the model of care implies changes to services delivery processes in response, including in the design of care, organization of providers, management of services and continuous performance improvement [212], [211].

**Multidisciplinary team** in primary care comprise of various primary care professionals: generalist medical practitioners, nurses, feldshers, specialist nurses, managers, support staff, family medicine and other primary care specialists [238].

**Municipal level** refers to a local administrative subdivision of the government that administers a city.

**National cancer screening programme** refers to a government-endorsed programme whereby screening is offered. NGO-let programmes or national recommendations for screening at the patient’s cost, do not qualify as a national screening programme [120].

**Out-of-pocket payments (OOP)** are payments for goods or services that include: (i) direct payments: payments for goods or services that are not covered by any form of insurance; [61] cost sharing: a provision of health insurance or third-party payment that requires the individual who is covered to pay part of the cost of health care received; and (iii) informal payments: unofficial payments for goods and services that should be fully funded from pooled revenue [209].

**Outpatient consultations/visits** include consultations/visits at the physician's office, consultations/visits in the patient's home, consultations/visits in outpatient departments in hospital, but excludes telephone contacts, visits for prescribed laboratory tests, visits to perform prescribed and scheduled treatment procedures, e.g. injections, physiotherapy, etc. visits to dentists, visits to nurses [192].

**Patient complaint system** is a formal, systematic and transparent process for receiving, investigating and resolving patients’ expressions of grievances or disputes with the care they received.

**Patient group**s include associations and organizations that provide organized insight and represent patient experiences as potential, current and past recipients of health services on general health topics or disease-specific areas [94].

**Patient list** refers to a list of records for each individual registered/assigned to/regularly seen by a provider. The list includes identification information, patient characteristics and may include information on current medical problems and on-going treatments. The list can exist in paper or electronic form.

**Patient registries** collect information over time on patients who are diagnosed with a particular disease or who receive particular treatments.

**Peer review meetings** (teams, committees, circles) are small groups of health professionals based on voluntary participation and concerned with activities aimed at accessing and continuously improving the quality of patient care.

**Peer support groups (peer-to-peer support)** are patient-driven groups on specific topics that encourage individuals to be in direct control of managing their conditions through group work and mutual support allowing them to draw on each other’s experiences.

**Periodic health audits and feedback** refers to any summary of clinical performance of health care over a specified period of time aimed at providing information to health professionals to allow them to assess and adjust their performance.

**Population-groups-specific reimbursement scheme** selects specific population groups (e.g. children, elderly, pensioners) for higher reimbursement rates or free medicines [50].

**Population stratification** (based on needs and risks), refers to the assessment of health needs for a given population, segmenting for epidemiological, demographic or geographic variables, for the planning and targeting of services to manage needs and proactively address known risk factors [212], [239].

**Positive list/reimbursement list** refers to the list of medicines that may be prescribed at the expense of the third-party payer.

**Postgraduate education programme** is part of tertiary education and corresponds to ISCED level 7. It typically varies from 1 to 4 years when following an undergraduate/bachelor’s programme (ISCED level 6), or from 5 to 7 years when directly following secondary education, ISCED level 3 [240].

**Post-natal care** **check** is understood as visits for the care of the mother (not the infant). It includes visits by a primary care health professional either at home or in a facility.

**Primary care** describes a type of care and setting for health services delivery that supports first-contact, accessible, continued, comprehensive and coordinated care to individuals and communities [212], [211], [207]. *See also* primary health care.

**Primary care performance assessment** includes publications (on paper or online) that systematically report on the performance of primary care in general, or important parts of the primary care system. These performance assessments may be used for monitoring, target setting and / or accountability. The focus of assessments is rather broad than detailed. Assessments do not include studies that evaluate specific interventions or programmes or studies that were solely done for academic purposes. A primary care assessment may also be part of an assessment of the health system in general. In replying to this question, please consider not only specific assessments dedicated to primary care, but also exercises that are part of larger reports or reports on specific forms of care (e.g. general practitioners, paediatricians, dentists, etc.). *See also* assessment.

**Primary health care expenditure** is currently being defined at the global level but it includes the following: all expenditures for providers who only provide primary health care services; expenditures for primary health care preventive services provided by additional providers; a proportion of overall capital costs; and, a proportion of administrative expenditures.

**Primary health care** refers to the approach elaborated in the 1978 Declaration of Alma-Ata based on the principles of equity, participation, intersectoral action, appropriate technology and a central role played by the health system for the delivery of services that are made universally accessible to individuals and families in the community through their full participation and at a cost that the community and country can afford to maintain at every stage of their development in the spirit of self-reliance and self-determination [212], [211], [207]. *See also* primary care.

**Product specific reimbursement scheme** determines eligibility based on the medicine in question; a medicine is either considered as reimbursable or as non-reimbursable [50].

**Provider payment: bundled payments** combine otherwise separate payments to providers into a single fee covering the care required for a person or defined population with multimorbidity for a predefined period. Payments can be bundled across providers and services and the price for the bundle can be set or negotiated [241].

**Provider payment: capitation** refers to a payment in which all providers in the payment system are paid, in advance, a predetermined fixed rate to provide a defined set of services for each individual enrolled with the provider for a fixed period [242].

**Provider payment: fee-for-service** is the case when providers are paid for each individual service provided. Fees are fixed in advance for each service or group of services [242].

**Provider payment: global budget** refers to the allocation of a payment fixed to a health care provider to cover the aggregate costs over a specific period to provide a set of services that have been broadly agreed on. A global budget may be based on inputs or outputs, or a combination of the two. Typically, providers have flexibility to make decisions about how to allocate funds across expenditure categories [242].

**Provider payment: pay-for performance** refers to a mechanism where the payment to providers is modified upwards or downwards based on the degree of target achievement reached [242].

**Public health services** refer to health services targeted at the population as a whole, These include, among others, health situation analysis, health surveillance, health promotion, prevention services, infectious diseases control, environmental protection and sanitation, disaster preparedness and response, and occupational health [209].

**Purchaser of health care** refers to financing agents as defined in the System of Health Account, i.e. the “final payer”. Depending on the country and type of service, purchasers either pay the provider directly or reimburse the patient after he/she receives care [225].

**Quality improvement teams/committees** refers to a group of individuals within a practice charged with carrying out improvement efforts. The team may report to management. Teams should meet regularly to review performance data, identify areas in need of improvement and carry out and monitor improvement efforts [235].

**Quality of care** refers to health system improvements sought for care that is effective, efficient, accessible, acceptable/patient-centered, equitable and safe [212], [243].

**Rapid tuberculosis diagnosis using WHO recommended rapid test** refers to the Xpert MTB/ RIF assay is the test endorsed by WHO to be used in countries most affected by tuberculosis. The test provides an accurate diagnosis for many patients in about 100 minutes, compared to previous tests which were required up to three months to receive results. More information regarding the test, frequently-asked questions and fact sheet can be found on the WHO tuberculosis programme’s web page: <http://www.who.int/tb/features_archive/new_rapid_test/en/>.

**Recurrent expenditures** are costs that refer to inputs which last less than one year and are regularly purchased for continuing an activity, such as salaries, drugs and supplies, repair maintenance, and others [209]. *See also* capital expenditure.

**Referral form** is a standardized form throughout the network of service providers that ensures that the same essential information is provided whenever a referral is initiated. It is normally designed to facilitate communication in both directions – the initiating facility completes the outward referral, **referral letter**, and at the end of care, the receiving facility completes the back referral to the original facility, **reply letter** [244].

**Referral letter** is part of the **referral form** and can be a stand alone document or included on one form with the **reply letter**. It is filled out by the initiating facility with information on the patient, the reason for referral and any clinical findings. It is used by the receiving facility to begin a through assessment of the patient and begin the management of the case [244].

**Referral guidelines** intend to map out the linkages across the different levels of the health system to ensure that health needs are addressed irrespective of the health system level at which care was first sought. It facilitates forward and backwards management of cases across different levels of care.

**Regional/oblast level** type of first level sub-national administrative division that may include several districts (second level administrative divisions).

**Reimbursement list** see *positive list*.

**Reply letter** is part of the referral form and can be a stand alone document or included on one form with the referral letter. It is filled out by the treating facility and contains information on special investigations, findings, diagnosis and treatment [244].

**Rural-urban classification** defines or delimits both urban and rural areas, or urban areas first and the latter by default. The classification may be defined on the basis of population in physical spaces with or without access to key services. In many countries, the criterion is population size or density, which are standard determinants of rurality. Rural areas are those with a low population density, i.e. a low number of inhabitants on a given area of land. Local administrative units may contain combinations of urban and rural populations. Several criteria may be combined (cities, municipalities, metropolitan areas) to define urban areas and define rural areas by exclusion [192].**Rural-urban classification** defines or delimits both urban and rural areas, or urban areas first and the latter by default. The classification may be defined on the basis of population in physical spaces with or without access to key services. In many countries, the criterion is population size or density, which are standard determinants of rurality. Rural areas are those with a low population density, i.e. a low number of inhabitants on a given area of land. Local administrative units may contain combinations of urban and rural populations. Several criteria may be combined (cities, municipalities, metropolitan areas) to define urban areas and define rural areas by exclusion [192].

**Scenario planning** defines a process of strategic planning that allows managers to explore various combinations of interventions to better understand what levels of intervention coverage and resources might be needed to achieve the desired results.

**Screening** is the presumptive identification of unrecognized disease in an apparently healthy, asymptomatic population by means of tests, examinations or other procedures that can be applied rapidly and easily to the target population [245].

**Self-management** or **self-care** is defined as the knowledge, skills and confidence to manage one’s own health, to care for a specific condition or to recover from an episode of ill health [134], [212].

**Settings of care** describe the varied types of arrangements for services delivery, organized further into different facilities, institutions and organizations that provide care. Settings include ambulatory, community, home, in-patient and residential services, whereas facilities refer to infrastructure, such as clinics, health centres, district hospitals, dispensaries, or other entities, for examples, mobile clinics and pharmacies [212], [116].

**Shared decision-making** is defined as an interactive process in which patients, their families and carers, in collaboration with their health provider(s), choose the next action(s) in their care path following an informed analysis of possible options, their values and preferences [134], [212].

**Stakeholder** refers to an individual, group or an organization that has an interest in the organization and delivery of health care [209].

**Strategy** refers to a series of time-bound broad lines of action intended to achieve a set of goals and targets set out within a policy programme [209], [246].

**Total risk approach** identifies individuals for prevention, treatment and referral based on a combined risk evaluation that includes age, sex, blood pressure, smoking status, total blood cholesterol and presence or absence of diabetes mellitus. This approach is considered more effective and less costly than informing treatment decisions based on a single factor, such as high arterial blood pressure or high serum cholesterol [219].

**Undergraduate/bachelor’s programme** is part of tertiary education and corresponds to International Standard Classification of Education (ISCED) level 6 program. It typically varies from 3 to 4 or more years when directly following upper secondary education (ISCED level 3) or 1 to 2 years when following another ISCED level 6 programme [240].

**Vocational training** refers to a short-cycle tertiary education programme corresponding to ISCED level 5. It may be referred to in many ways, for example: (higher) technical education, community college education, technician or advanced/higher vocational training, associate degree, or bac+2. It is designed for learners to acquire the knowledge, skills and competencies specific to a particular occupation or class of occupations. Successful completion of such programmes leads to labour market-relevant, vocational qualifications acknowledged as occupationally-oriented by the relevant national authorities and/or the labour market [240].

**WHO/ISH cardiovascular risk prediction charts** indicate 10-year risk of a fata or not fatal major cardiovascular event taking into consideration age, sex, blood pressure, smoking status, total blood cholesterol and presence or absence of diabetes mellitus. There are specifi charts for 14 WHO epidemiological subregions. A separate set of charts are available for settings where blood cholesterol cannot be measured. These can be further calibrated at country-level. Detailed information can be found at:

http://www.who.int/cardiovascular_diseases/guidelines/Chart_predictions/en/

**References**

1. Wagner, E., *Care for older people with chronic illness.* Older People: Building Systems Based on Evidence, 1999: p. 39-64.

2. Commonwealth Fund, *Framework for a high performance health system for the United States*. 2006, The Commonwealth Fund: New York.

3. Flocke, S., *Measuring attributes of primary care: development of a new instrument* J Fam Pract, 1997. **45**(1): p. 64-74.

4. Roberts, M., et al., *Getting health reform right: a guide to improving performance and equity*. 2008, Oxford University Press: Oxford.

5. Aday, L.A., et al., *A framework for assessing the effectiveness, efficiency, and equity of behavioral healthcare.* Am J Manag Care, 1999. **5 Spec No**: p. Sp25-44.

6. Pina, I.L., et al., *A framework for describing health care delivery organizations and systems.* Am J Public Health, 2015. **105**(4): p. 670-9.

7. Tello, J. and E. Barbazza, *Health services delivery: a concept note*. 2015, WHO Regional Office for Europe: Copenhagen

8. International Health Partnership, *A common framework for monitoring performance and evaluation of the scale-up for better health*. 2008, WHO and World Bank,: Geneva

9. Kelley, J. and J. Hurst, *Health care quality indicators project conceptual framework paper*, in *OECD Health Working Papers No. 23*. 2006, OECD: Paris.

10. Centre for Policy Studies in Family Medicine and Primary Care, *The Patient Centred Medical Home: history, seven core features, evidence and*

*transformational change*. 2007, Robert Graham Center Washington, D.C.

11. Hurst, J. and M. Jee-Hughes, *Performance measurement and performance management in OECD health systems*, in *OECD Labour Market and Social Policy Occassional Papers No. 47*. 2001, OECD: Paris.

12. Shi, L., B. Starfield, and J. Xu, *Validating the adult primary care assessment tool* J Fam Pract, 2001. **50**(2): p. 161-164.

13. Alliance for Health Policy and Systems Research, *Report of the expert consultation on primary care systems profiles and performance (PRIMASYS)*. 2015, World Health Organization Geneva.

14. Kringos, D.S., et al., *The European primary care monitor: structure, process and outcome indicators.* BMC family practice, 2010. **11**: p. 81-81.

15. Veillard, J., et al., *Better Measurement for Performance Improvement in Low- and Middle-Income Countries: The Primary Health Care Performance Initiative (PHCPI) Experience of Conceptual Framework Development and Indicator Selection.* Milbank Q, 2017. **95**(4): p. 836-883.

16. Wendt, D., *Health systems rapid diagnostic tool*. 2012, Family Health International: North Carolina

17. Schlette, S., et al., *The Bellagio Model: an evidence informed, international framework for population-oriented primary care.* Z Evid Fortbild Qual Gesundwes, 2009 **103**(7): p. 467-74.

18. USAID, *The health system assessment approach: a how-to manual 2.0*. 2012, USAID: Washington, D.C.

19. WHO Regional Office for Europe, *Assessing health services delivery performance with hospitalizations for ambulatory care sensitive conditions*. 2016, WHO Regional Office for Europe: Copenhagen

20. WHO Regional Office for Europe, *Better noncommunicable disease outcomes: challenges and opportunities for health systems. Country Assessment guide*. 2014, WHO Regional Office for Europe Copenahgen

21. WHO Regional Office for Europe, *Towards people-centred health systems: an innovative approach for better health outcomes* 2013, WHO Regional Office for Europe Copenhagen.

22. WHO Regional Office for Europe, *Priorities for health systems strengthening in the European Region 2015-2020: walking the talk on people centredness*. 2015, WHO Regional Office for Europe: Copenhagen.

23. WHO Regional Office for Europe, *Self-assessment tool for the evaluation of essential public health operations in the WHO European Region*. 2015, WHO Regional Office for Europe: Copenhagen.

24. World Health Organization, *Framework of indicators and targets for laboratory strengthening under the end TB strategy*. 2016, World Health Organization Geneva

25. World Health Organization, *Framework on integrated, people-cenred health services* 2016, World Health Organization Geneva

26. World Health Organization, *The World Health Report 2000: Health Systems: Improving Performance*. 2000, World Health Organization Geneva

27. World Health Organization, *WHO: Everybody’s business: strengthening health systems for improved health outcomes: WHO’s framework for action [Internet]*. 2007, Geneva: WHO Press.

28. Rechel, B., S. Thomson, and E. van Ginneken, *Health Systems in Transition: Template for authors* 2010, WHO Regional Office for Europe Copenhagen

29. World Health Organization, *Package of essential noncommunicable disease interventions for primary health care in low-resource settings*. 2010, World Health Organization Geneva.

30. Pan American Health Organization, *Integrated Health Service Delivery Networks: Concepts, Policy Options and a Road Map for Implementation in the Americas*. 2011, PAHO: Washington, D.C.

31. WHO Regional Office for Europe, *Performance Assessment Tool for Quality Improvement in Hospitals* 2007, WHO Regional Office for Europe Copenhagen

32. WHO Regional Office for Europe, *Primary care evaluation tool*. 2010, WHO Regional Office for Europe: Copenhagen.

33. Adams, O., et al., *Provision of personal and non-personal health services: proposal for monitoring* 2002, World Health Organization: Geneva.

34. Atun, R. and N. Mendabde, *Health systems and systems thinking*, in *Health Systems and the challenges of communicable diseases: experiences from Europe and Latin America*, E.O.o.H.S.a. Policies, Editor. 2008, WHO Regional Office for Europe: Copenhagen.

35. Hogan, D., A. Hosseinpoor, and T. Boerma, *Developing an index for the coverage of essential health services* 2016, World Health Organization Geneva

36. The Commonwealth Fund, *2015 Commonwleath Fund International Survey of Primary Care Physicians in 10 Nations*. 2015, The Commonwealth Fund: Washington, DC.

37. WHO Regional Office for Europe, *Availability of national health services delivery data across the WHO European Region: scanning survey results* 2018, WHO Regional Office for Europe Copenhagen

38. European Centre for Disease Prevention and Control, *Seasonal influenza vaccination in Europe. Vaccination recommendations and coverage rates in the EU Member States for eight influenza seasons: 2007–2008 to 2014–2015*. 2017, ECDC: Stockholm

39. WHO, *Third global survey on eHealth*. 2015, WHO: Geneva.

40. Expert Panel on effective ways of investing in health, *Tools and methodologies for assessing the performance of primary care*. 2018, European Commission Brussels.

41. OECD, *Strengthening health information infrastructure for health care quality governance* in *OECD Health Policy Studies* 2013, OECD: Paris

42. Oderkirk J, *Readiness of electronic health record systems to contribute to national health information and research* 2017, OECD Health Working Papers Paris

43. OECD. *OECD Health System Characteristics Survey*. 2016; Available from: <https://www.oecd.org/els/health-systems/characteristics.htm>.

44. Paris, V., De Lagasnerie, G & Fujisawa, R *How Do OECD Countries Define the Basket of Goods and Services Financed Collectively?* 2014, Center for Health Care Strategies: New Jesey.

45. OECD, *Patient-reported indicators survey*. 2017, OECD: Paris.

46. Fujisawa, R. and N. Klazinga, *Measuring patient experience (PREMS): Progress made by the OECD and its member countries between 2006 and 2016* in *OECD Health Working Papers* 2017, OECD Publishing Paris

47. Schafer, W.L., et al., *QUALICOPC, a multi-country study evaluating quality, costs and equity in primary care.* BMC Fam Pract, 2011. **12**: p. 115.

48. Wendt, D., et al., *Health system rapid diagnostic tool.* Durham, NC: FHI, 2013. **360**.

49. World Health Organization, *Service Availability and Readiness Assessment (SARA): an annual monitoring system for service delivery*, in *Reference Manual, Version 2.2*. 2015, World Health Organization Geneva.

50. WHO Regional Office for Europe, *Medicines reimbursement policies in Europe,* . 2018, WHO Regional Office for Europe Copenhagen

51. World Health Organization, *Assessing national capacity for the prevention and control of noncommunicable diseases* 2017, World Health Organization: Geneva.

52. World Health Organization, *The WHO STEPwise approach to noncommunicable disease risk factor surveillance (STEPS)*. 2017, World Health Organization Geneva.

53. WHO Regional Office for Europe, *WHO Regional Office for Europe Antimicrobial Medicines Consumption Network* 2017, WHO Regional Office for Europe Copenhagen

54. London School of Hygiene and Tropical Medicine. *CONCORD Programme* 2019; Available from: <https://csg.lshtm.ac.uk/research/themes/concord-programme/>.

55. European Commission. *European Core Health Indicators (ECHI)*. Indicators and Data 2018; Available from: <https://ec.europa.eu/health/indicators_data/echi_en>.

56. European Commission. *Eurostat database*. 2018; Available from: <https://ec.europa.eu/eurostat/data/database>.

57. WHO Regional Office for Europe, *European database on human and technical resources for health*, in *European Health Information Gateway*, W.R.O.f. Europe, Editor. 2016, WHO Regional Office for Europe: Copenhagen.

58. WHO Regional Office for Europe. *Morbidity, disability and hospital discharges* European Health Information Gateway 2018; Available from: <https://gateway.euro.who.int/en/datasets/european-health-for-all-database/#morbidity-disability-and-hospital-discharges>.

59. World Health Organization. *Global Health Estimates (GHE)*. Health statistics and information systems 2018; Available from: <https://www.who.int/healthinfo/global_burden_disease/en/>.

60. World Health Organization. *Global Health Expenditure Database*. 2018; Available from: <http://apps.who.int/nha/database>.

61. Allemani, C., et al., *Global surveillance of trends in cancer survival 2000&#x2013;14 (CONCORD-3): analysis of individual records for 37&#x2008;513&#x2008;025 patients diagnosed with one of 18 cancers from 322 population-based registries in 71 countries.* The Lancet, 2018. **391**(10125): p. 1023-1075.

62. World Health Organization. *Global health observatory* 2018; Available from: <https://www.who.int/gho/en/>.

63. International Narcotics Control Board, *Narcotic drugs 2017 - Estimated world requirements for 2018* 2017, International Narcotics Control Board Vienna

64. World health Organization, *Global tuberculosis report 2018*. 2018, World Health Organization Geneva.

65. Europe, W.R.O.f. *Health 2020 indicators*. 2018; Available from: <https://gateway.euro.who.int/en/datasets/health-2020-indicators/>.

66. WHO Regional Office for Europe. *European health for all database*. 2017 [cited 2017; Available from: <http://data.euro.who.int/hfadb/>.

67. International Labour Organization. *ILOSTAT database* 2019; Available from: <https://www.ilo.org/ilostat/faces/oracle/webcenter/portalapp/pages/statistics/download.jspx;ILOSTATCOOKIE=1VFLoVDmeM0ZBEDXcxwZy16OJBUIuRBeOVOdPAD1LFScdz9ilHsN!1595421183?_adf.ctrl-state=yg4z6hr2m_4&_afrLoop=1026445541041688&_afrWindowMode=0&_afrWindowId=null#!%40%40%3F_afrWindowId%3Dnull%26_afrLoop%3D1026445541041688%26_afrWindowMode%3D0%26_adf.ctrl-state%3D15zgscxpu9_4>.

68. OECD. *OECD Health Statistics 2018*. 2018; Available from: <http://www.oecd.org/els/health-systems/health-data.htm>.

69. World Health Organization. *Health Accounts*. 2018; Available from: <https://www.who.int/health-accounts/en/>.

70. World Health Organization. *Universal health coverage* The Global Health Observatory 2018; Available from: <http://apps.who.int/gho/portal/uhc-overview.jsp>.

71. World Health Organization. *MedMon–WHO Essential Medicines and Health Products Price and Availability Monitoring Mobile Application* 2019; Available from: <https://www.who.int/medicines/areas/policy/monitoring/empmedmon/en/>.

72. United Nations. *World population prospects*. 2017; Available from: <https://population.un.org/wpp/>.

73. WHO Regional Office for Europe and European Observatory on Health Systems and Policies. *Health system reviews (HiT series)* 2018 [cited 2019 10 January]; Available from: <http://www.euro.who.int/en/about-us/partners/observatory/publications/health-system-reviews-hits>.

74. OECD. *OECD reviews of health systems: a series of country reports*. 2018; Available from: <http://www.oecd.org/els/health-systems/reviews-health-systems.htm>.

75. OECD. *Reviews of National Health Care Quality* 2018; Available from: <http://www.oecd.org/health/health-care-quality-reviews.htm>.

76. WHO Regional Office for Europe. *Health systems response to NCDs - country assessments* 2018; Available from: <http://www.euro.who.int/en/health-topics/Health-systems/health-systems-response-to-ncds/publications/country-assessments>.

77. WHO Regional Office for Europe. *Universal health coverage: financial protection country reviews* 2019; Available from: <http://www.euro.who.int/en/health-topics/Health-systems/health-systems-financing/publications/clusters/universal-health-coverage-financial-protection/universal-health-coverage-financial-protection-country-reviews>.

78. WHO Regional Office for Europe. *Publications - Evaluation of the organization and provision of primary care series* 2015; Available from: <http://www.euro.who.int/en/health-topics/Health-systems/primary-health-care/publications/a-z-list-of-all-publications>.

79. World Health Organization, *Noncommunicable disease country profiles 2018*. 2018, World Health Organization Geneva

80. WHO Regional Office for Europe, *Action Plan for Sexual and Reproductive Health: towards achieving the 2030 Agenda for Sustainable Development in Europe – leaving no one behind*. 2016, WHO Regional Office for Europe Copenhagen

81. WHO Regional Office for Europe, *Action plan for the health sector response to HIV in the WHO European Region 2016–2021*. 2017, WHO Regional Office for Europe Copenhagen

82. WHO Regional Office for Europe, *Action plan for the health sector response to viral hepatitis in the WHO European Region 2016–2021*. 2017, WHO Regional Office for Europe Copenhagen

83. WHO Regional Office for Europe, *Action plan for the prevention and control of noncommunicable diseases in the WHO European Region 2016-2025*. 2016, WHO Regional Office for Europe: Copenhagen

84. WHO Regional Office for Europe, *The European Mental Health Action Plan 2013–2020* 2015, WHO Regional Office for Europe Copenhagen

85. WHO Regional Office for Europe, *European Vaccine Action Plan 2015–2020* 2014, WHO Regional Office for Europe Copenhagen

86. World Health Organization, *Global Action Plan for the Prevention and Control of NCDs 2013–2020*. 2013, WHO Geneva.

87. WHO Regional Office for Europe, *Health 2020: a European policy framework supporting action across government and society for health and well-being [Internet]. Copenhagen: WHO Regional Office for Europe*. 2013, WHO Regional Office for Europe: Copenhagen.

88. WHO Regional Office for Europe, *Investing in children: the European child and adolescent health strategy 2015–2020*. 2014, WHO Regional Office for Europe: Copenhagen

89. WHO Regional Office for Europe, *Strategy and action plan for healthy ageing in Europe 2012–2020*. 2012, WHO Regional Office for Europe Copenhagen

90. WHO Regional Office for Europe, *Strategy on women’s health and well-being in the WHO European Region 2017–2021* 2016, WHO Regional Office for Europe Copenhagen

91. United Nations, *Transforming our world: the 2030 Agenda for Sustainable Development* in *Seventieth session Agenda items 15 and 116*. 2015, United Nations New York

92. WHO Regional Office for Europe, *Tuberculosis action plan for the WHO European Region 2016–2020*. 2015, WHO Regional Office for Europe Copenhagen

93. WHO Regional Office for Europe, *Health 2020: A European policy framework supporting action across government and society for health and well-being*. 2013, WHO Regional Office for Europe: Copenhagen.

94. Borgermans, L. and M. Langins, *Strengthening a competent health workforce for the provision of coordinated/integrated health services*. 2015, World Health Organization Regional Office for Europe: Copenhagen.

95. Kringos, D.S., et al., *The European primary care monitor: structure, process and outcome indicators.* BMC Fam Pract, 2010. **11**: p. 81.

96. *Package of essential noncommunicable (PEN) disease interventions for primary health care in low-resource settings*. 2010, World Health Organization: Geneva.

97. *Methodology note*. 2015, Primary Health Care Performance Initiative: Washington, DC.

98. Cashin, C., et al., *Paying for performance in health care: implications for health system performance and accountability*. European Observatory on Health Systems and Policies series. 2014, Maidenhead, Berkshire, England: McGraw Hill Education, Open University Press. xxiii, 312 pages.

99. *Resolution WHA64.6 on health workforce strengthening*, in *Resolutions and decisions, annexes*. 2011, World Health Organization: Geneva.

100. Dolea, C. and World Health Organization., *Increasing access to health workers in remote and rural areas through improved retention : global policy recommendations*. 2010, Geneva: World Health Organization. iii, 72 p.

101. WHO Regional Office for Europe, *Strengthening people-centred health systems in the WHO European Region: framework for action on integrated health services delivery* in *Regional Committee for Europe 66th Session*. 2016, WHO Regional Office for Europe: Copenhagen.

102. Barbazza, E., et al., *Health workforce governance: Processes, tools and actors towards a competent workforce for integratd health srevices delivery.* Health Policy, 2015.

103. *Human resources for health information system : minimum data set for health workforce registry*. 2015, Geneva: World Health Organization. viii, 67 pages.

104. *Resolution WHA69.19 on global strategy on human resources for health: workforce 2030*, in *Resolutions and decisions, annexes*. 2016, World Health Organization: Geneva.

105. Organisation for Economic Co-operation and Development, *Health at a glance*. 2015, Paris: OECD Publishing.

106. *Monitoring the building blocks of health systems: a handbook of indicators and their measurement strategies*. 2010, Geneva: World Health Organization. xii, 92 p.

107. Akturk, Z., et al., *The role of family medicine in undergraduate medical education*, in *The World Book of Family Medicine European Edition*2015, Wonca Europe, the World Organization of National Colleges, Academies and Academic Associations of General Practitioners/Family Physicians.

108. Zarbailov, N., et al., *Strengthening general practice/family medicine in Europe-advice from professionals from 30 European countries.* BMC Fam Pract, 2017. **18**(1): p. 80.

109. *Wonca global standards for postgraduate family medicine education*, in *Wonca Working Party on Education*. 2013, The World Organization of National Colleges Academies and Academic Associations of General Practitioners/Family Physicians.

110. Maier, C.B. and L.H. Aiken, *Task shifting from physicians to nurses in primary care in 39 countries: a cross-country comparative study.* Eur J Public Health, 2016. **26**(6): p. 927-934.

111. Organisation for Economic Co-operation and Development, *Strengthening health information infrastructure for health care quality governance: good practices, new opportunities and data privacy protection challenges*. 2013, OECD Publishing: Paris.

112. Noncommunicable Diseases and Mental Health Cluster and Chronic Diseases and Health Promotion Department, *Preparing a health care workforce for the 21st century. The challenge of chronic conditions.* 2005, World Health Organization.

113. Peterson, C.B., et al., *From innovation to implementation: eHealth in the WHO European Region*. 2016, Copenhagen: WHO Regional Office for Europe. xiii, 98 pages.

114. Government of Scotland, *Making it easy: A health literacy action plan for Scotland*. 2014, Government of Scotland: Edinburgh.

115. United Nations Department of Economic and Social Affairs Statistics Division. *Metadata for indicator 3.b.3 Proportion of Health facilities that have a core set of relevant essential medicines available and affordable on a sustainable basis*. 2018; Available from: <https://unstats.un.org/sdgs/metadata/files/Metadata-03-0B-03.pdf>.

116. Tello, J. and E. Barbazza, *Health services delivery: a concept note*. 2015, World Health Organization Regional Office for Europe: Copenhangen.

117. *People-centred and integrated health services: an overview of the evidence. Interim report*. 2015, World Health Organization: Geneva.

118. *Planning, implementation and assessment: assessment of capacity (sample questionnaire)*, in *Tools for implementing WHO PEN (Package of essential noncommunicable disease interventions)*. 2010, World Health Organization: Geneva.

119. *Framework of indicators and targets for laboratory strengthening under the End TB Strategy*. 2016, World Health Organization: Geneva.

120. Primary Health Care Performance Initiative, *Primary Health Care Performance Initiative: Methodology Note*. 2015, Primary Health Care Performance Initative: Washington, DC.

121. *List of medical devices by health care facility*. 2015, World Health Organization: Geneva.

122. *Noncommunicable diseases global monitoring framework: indicator definitions and specifications*. 2014, World Health Organization: Geneva.

123. Scholz, S., B. Ngoli, and S. Flessa, *Rapid assessment of infrastructure of primary health care facilities - a relevant instrument for health care systems management.* BMC Health Serv Res, 2015. **15**: p. 183.

124. *Assessing national capacity for the prevention and control of noncommunicable diseases: report of the 2015 global survey.* 2016, World Health Organization: Geneva.

125. *Global action plan for the prevention and control of noncommunicable diseases 2013-2020.* 2013, World Health Organization: Geneva.

126. European Centre for Disease Prevention and Control, *Seasonal influenza vaccination in Europe - vaccination recommendations and coverage rates for eight influenza seasons* 2017, ECDC: Stockholm.

127. Rechel, B., et al., *The Soviet legacy in diagnosis and treatment: Implications for population health.* J Public Health Policy, 2011. **32**(3): p. 293-304.

128. Primary Health Care Classification Consortium. *International Classification of Primary Care 2*. 2017 19 December 2017 [cited 2018 19 January]; Available from: <https://class.who-fic.nl/browser.aspx>.

129. Calouste Gulbenkian Foundation and World Health Organization, *Improving access to and appropriate use of medicines for mental disorders*. 2017, World Health Organization: Geneva.

130. *Better noncommunicable disease outcomes: challenges and opportunities for health systems. Assessment Guide*. 2014, World Health Organization Regional Office for Europe: Copenhagen.

131. *HEARTS Technical package for cardiovascular disease management in primary health care: systems for monitoring*. 2018, World Health Organization: Geneva.

132. *Guidelines for treatment of drug-susceptible tuberculosis and patient care, 2017 update*. 2017, World Health Organization: Geneva.

133. *A people-centred model of TB care. Blueprints for EECA countries*. 2017, World Health Organization Regional Office for Europe: Copenhagen.

134. Ferrer, L., *Engaging patients, carers and communities for the provision of coordinated/integrated health services: strategies and tools*. 2015, World Health Organization Regional Office for Europe: Copenhagen.

135. Kickbusch, I., et al., *Health literacy: the solid facts*. 2013, World Health Organization Regional Office for Europe: Copenhagen.

136. Sykes, S., et al., *Understanding critical health literacy: a concept analysis.* BMC Public Health, 2013. **13**: p. 150.

137. Bayliss, E.A., et al., *Supporting self-management for patients with complex medical needs: recommendations of a working group.* Chronic Illn, 2007. **3**(2): p. 167-75.

138. Boult, C. and G.D. Wieland, *Comprehensive primary care for older patients with multiple chronic conditions: "Nobody rushes you through".* JAMA, 2010. **304**(17): p. 1936-43.

139. *Integrated care models: an overview*. 2016, World Health Organization Regional Office for Europe: Copenhagen.

140. Ham, C., *Working together for health: acheivements and challenges in the Kaiser NHS Beacon sites programme.* University of Birmingham Health Services Management Centre policy papers, 2010. **6**.

141. Bielaszka-DuVernay, C., *The 'GRACE' model: in-home assessments lead to better care for dual eligibles.* Health Aff (Millwood), 2011. **30**(3): p. 431-4.

142. Bayliss, E.A., et al., *Processes of care desired by elderly patients with multimorbidities.* Fam Pract, 2008. **25**(4): p. 287-93.

143. Boeckxstaens, P. and P. De Graaf, *Primary care and care for older persons: position paper of the European Forum for Primary Care.* Qual Prim Care, 2011. **19**(6): p. 369-89.

144. Bleijenberg, N., et al., *Exploring the expectations, needs and experiences of general practitioners and nurses towards a proactive and structured care programme for frail older patients: a mixed-methods study.* J Adv Nurs, 2013. **69**(10): p. 2262-73.

145. Boult, C., et al., *The effect of guided care teams on the use of health services: results from a cluster-randomized controlled trial.* Arch Intern Med, 2011. **171**(5): p. 460-6.

146. Bonifas, R., D. Gammonley, and K. Simons, *Gerontological social workers' perceived efficacy for influencing client outcomes.* J Gerontol Soc Work, 2012. **55**(6): p. 519-36.

147. Counsell, S.R., et al., *Geriatric care management for low-income seniors: a randomized controlled trial.* JAMA, 2007. **298**(22): p. 2623-33.

148. Callahan, C.M., et al., *Implementing dementia care models in primary care settings: the aging brain care medical home.* Aging Ment Health, 2011. **15**(1): p. 5-12.

149. Goodwin, N., et al., *Providing integrated care for older people with complex needs : lessons from seven international case studies*. 2014, London: The King's Fund. 28 pages.

150. *Report of the first meeting*. in *Primary Health Care Advisory Group meeting*. 2017. Almaty, Kazakhstan: World Health Organization Regional Office for Europe.

151. Paris, V., M. Devaux and L. Wei, *Health systems institutional characteristics*. 2010, Paris: OECD Publishing.

152. Cheng, S.H. and C.C. Chen, *Effects of continuity of care on medication duplication among the elderly.* Med Care, 2014. **52**(2): p. 149-56.

153. Boeckxstaens, P., et al., *A practice-based analysis of combinations of diseases in patients aged 65 or older in primary care.* BMC Fam Pract, 2014. **15**: p. 159.

154. *Medical equipment maintenance programme overview. WHO medical device technical series*. 2011, World Health Organization: Geneva.

155. *Patient engagement*, in *Technical series on safer primary care*. 2016, World Health Organization: Geneva.

156. *Influenza vaccination*. 2017 [cited 2017 26 October]; Available from: <http://www.euro.who.int/en/health-topics/communicable-diseases/influenza/vaccination>.

157. *Action plan for the prevention and control of noncommunicable diseases in the WHO European Region 2016–2025*. 2016, World Health Organization Regional Office for Europe: Copenhagen.

158. Tobacco Free Initiative. *Quitting tobacco: what kind of support is available*. 2017 [cited 2017 October 26]; Available from: <http://www.who.int/tobacco/quitting/background/en/index2.html>.

159. *The WHO STEPwise approach to noncommunicable disease risk factor surveillance. Manual*. 2017, World Health Organization: Copenhagen.

160. Organisation for Economic Co-operation and Development, *Health Statistics. Definitions, sources and methods. Cervical cancer screening, survey data and programme data*. 2017, OECD Publishing: Paris.

161. *Global tuberculosis report 2017*. 2017, World Health Organization: Geneva.

162. Institute for Health Metrics and Evaluation, *Data visualizations*. 2013, Institute for Health Metrics and Evaluation: Seattle.

163. *Revision of Automated real-time nucleic acid ampli cation technology for rapid and simultaneous detec- tion of tuberculosis and rifampicin resistance: Xpert MTB/RIF system. Policy statement.* 2013, World Health Organization: Geneva.

164. *The use of loop-mediated isothermal amplification (TB-LAMP) for the diagnosis of pulmonary tuberculosis: policy guideliens*. 2016, World Health Organization: Geneva.

165. *Mental health action plan: 2013-2020*. 2013, World Health Organization: Geneva.

166. *WHO recommendations on: postnatal care of the mother and newborn*. 2013, World Health Organization: Geneva.

167. *WHO recommendations on mental health - duration of antidepressant treatment*. 2012, World Health Organization: Geneva.

168. Kringos, D., et al., *Building primary care in a changing Europe*, in *Observatory Studies Series*. 2015, European Observatory on Health Systems and Policies, a partnership hosted by WHO: United Kingdom.

169. Evans, D.B., J. Hsu, and T. Boerma, *Universal health coverage and universal access.* Bull World Health Organ, 2013. **91**(8): p. 546-546A.

170. OECD/EU, *Health at a glance: Europe*. 2016, Paris: OECD Publishing.

171. Global Health Workforce Alliance. *What do we mean by availability, accessibility, acceptability and quality (AAAQ) of the health workforce?* [cited 2017 November 1]; Available from: <http://www.who.int/workforcealliance/media/qa/04/en/>.

172. Bruckert, E., et al., *Assessment of cardiovascular risk in primary care patients in France.* Arch Cardiovasc Dis, 2011. **104**(6-7): p. 381-7.

173. Barrios, V., et al., *Cardiovascular risk profile and risk stratification of the hypertensive population attended by general practitioners and specialists in Spain. The CONTROLRISK study.* J Hum Hypertens, 2007. **21**(6): p. 479-85.

174. McIntyre, D. and J. Kutzin, *Health Financing Guidance no 1. Health financing country diagnostic: a foundation for national strategy development*. 2016, World Health Organization: Geneva.

175. World Health Organization, *Ensuring balance in national policies on controlled substances: guidance for availability and accessibility of controlled medicines*. 2011, World Health Organization: Geneva.

176. WHO Regional Office for Europe, *Antimicrobial medicines consumption data 2011-2014*. 2017, WHO Regional Office for Europe: Copenhagen

177. Health and Social Care Alliance Scotland. *Professional resources: where people need support. My condition, my terms, my life.* 2016 [cited 2016 17 August 2016]; Available from: <http://www.myconditionmylife.org>.

178. Mackenbach, J.P. and M. McKee, *Successes and failures of health policy in Europe : four decades of divergent trends and converging challenges*. European observatory on health systems and policies series. 2013, Maidenhead, Berkshire: McGraw Hill Education : Open University Press. xix, 371 pages.

179. OECD, *Health at a Glance: Europe 2018*. 2018, OECD: Paris

180. *Roadmap to prevent and combat drug-resistant tuberculosis. The consolidated action plan to prevent and combat multi drug- and extensively drug-resistant tuberculosis in the WHO European Region, 2011-2015*. 2011, World Health Organization Regional Office for Europe: Copenhagen.

181. Balicer, R.D., et al., *Reducing health disparities: strategy planning and implementation in Israel's largest health care organization.* Health services research, 2011. **46**(4): p. 1281-1299.

182. Minicozzi, P., et al., *Quality analysis of population-based information on cancer stage at diagnosis across Europe, with presentation of stage-specific cancer survival estimates: A EUROCARE-5 study.* Eur J Cancer, 2017. **84**: p. 335-353.

183. International Narcotics Control Board, *Report 2016. Estimated World Requriements for 2017. Statistics for 2015*. 2016, United Nations Publications: New York.

184. *Pan-Canadian primary health care indicators: pan-Canadian primary health care indicator development project, report 1*. 2006, Ottawa: Canadian Institute for Health Information, Institut canadien d'information sur la santé.

185. *Global status report on alcohol and health*. 2014, Geneva: World Health Organization. xiv, 376 pages.

186. Rehm, J., et al., *Global burden of disease and injury and economic cost attributable to alcohol use and alcohol-use disorders.* Lancet, 2009. **373**(9682): p. 2223-33.

187. World Health Organization Statistical Information System, *Indicator definitions and metadata*. 2017, World Health Organization: Geneva.

188. *Worldwide trends in diabetes since 1980: a pooled analysis of 751 population-based studies with 4.4 million participants.* Lancet, 2016. **387**(10027): p. 1513-30.

189. Roglic, G. and World Health Organization, *Global health report on diabetes: 2016*. 2016, World Health Organization Geneva.

190. Eurostat. *Amenable and preventable deaths statistics*. Statistics Explained 2017 [cited 2018 17 January]; Available from: <http://ec.europa.eu/eurostat/statistics-explained/index.php/Amenable_and_preventable_deaths_statistics>.

191. *Fact sheets on sustainable development goals: health targets. Noncommunicable diseases*. 2017, World Health Organization Regional Office for Europe: Copenhagen.

192. *National Health Workforce Accounts: a handbook. Glossary*. 2017, World Health Organization: Geneva.

193. Care, C.f.C. *Personalised care and support planning handbook*. 2015; Available from: <https://www.england.nhs.uk/wp-content/uploads/2015/01/pers-care-guid-core-guid.pdf>.

194. Colombo, F., et al., *The impact of caring on family carers*, in *Help wanted? Providing and paying for long-term care*. 2011, OECD Publishing: Paris. p. 85-120.

195. Department of Statistics of the International Labour Organization, *International Standard Classification of Occupations ISCO-08*. 2012, International Labour Office: Geneva.

196. Directorate-General for employment, s.a.a.i. *Generalist medical practitioners. European skills/competences, qualifications and occupations*. 2017 [cited 2018 22 January]; Available from: <http://data.europa.eu/esco/isco/C2211>.

197. Directorate-General for employment, s.a.a.i. *Health associate professionals. European skills/competences, qualifications and occupations*. 2017 [cited 2018 22 January]; Available from: <http://data.europa.eu/esco/isco/C32>.

198. Directorate-General for employment, s.a.a.i. *Health professionals. European skills/compentences, qualifications and occupations*. 2017 [cited 2018 22 January]; Available from: <http://data.europa.eu/esco/isco/C22>.

199. Kyratsis, Y., et al., *Health Systems in transition: professional identity work in the context of shifting institutional logics.* Academy of Management Journal, 2016.

200. *Nurse practitioner/advanced practice nurse: definition and characteristics*. Nursing Matters 2009 [cited 2018 17 January]; Available from: <http://www.icn.ch/images/stories/documents/publications/fact_sheets/1b_FS-NP_APN.pdf>.

201. Directorate-General for employment, s.a.a.i. *Paramedical practitioners. European skills/competences, qualifications and occupations*. 2017 [cited 2018 22 January]; Available from: <http://data.europa.eu/esco/isco/C224>.

202. Directorate-General for employment, s.a.a.i. *Medical doctors. European skills/competences, qualifications and occupations*. 2017 [cited 2018 22 January]; Available from: <http://data.europa.eu/esco/isco/C221>.

203. OECD, *OECD Health Data 2001: a comparative analysis of 30 countries* 2001, OECD Paris.

204. Bjegovic-Mikanovic, V., et al., *Policy Summary 10: Addressing needs in the public health workforce in Europe*, E.O.o.H.S.a. Policies, Editor. 2014, World Health Organization Regional Office for Europe: Copenhagen.

205. Directorate-General for employment, s.a.a.i. *Specialist medical practitioners. European skills/competences, qualifications and occupations*. 2017 [cited 2018 22 January]; Available from: <http://data.europa.eu/esco/isco/C2212>.

206. Organisation for Economic Co-operation and Development, World Health Organization, and Statistical Office of the European Communities, *A system of health accounts 2011*. 2011 ed. 2011, Paris, Luxembourg, Geneva: OECD; Eurostat; World Health Organization. 521 p.

207. WHO European Centre for Primary Health Care, *Roadmap to monitoring health services delivery in the WHO European Region*. 2017, World Health Organization Regional Office for Europe Copenhagen.

208. *A glossary of terms for community health care and services for older persons*. 2004, World Health Organization: Geneva.

209. *WHO health systems strengthening glossary*. 2011, World Health Organization: Geneva.

210. Starfield, B., *Basic concepts in population health and health care.* Journal of Epidemiology & Community Health, 2001. **55**: p. 452-454.

211. *Strengthening people-centred health systems in the WHO European Region: framework for action on integrated health services delivery*. in *WHO Regional Committe for Europe 66th session*. 2016. Copenhagen.

212. *Glossary of terms. The European Framework for Action on Integrated Health Services Delivery*. 2016, World Health Organization Regional Office for Europe: Copenhagen.

213. Ettelt, S.e.a., *Health care outside hospital: accessing generalist and specialist care in eight countries.* 2006, WHO Regional Office for Europe: Copenhagen.

214. Rooney, A.L. and P.R. van Ostenberg, *Licensure, accreditation, and certification: approaches to health services quality. Quality assurance methodology refinement series*. 1999, USAID: Bethesda, MD.

215. Barbazza, E. and J. Tello, *A review of health governance: Definitions, dimensions and tools to govern* Health Policy 2014. **116**: p. 1-11.

216. European Observatory on Health Systems and Policies and Asia Pacific Observatory on Health Systems and Policies, *Health systems in transition. Template for authors.* 2016, World Health Organization: Geneva.

217. *Assessing health services delivery performance with hospitalizations for ambulatory care sensitive conditions*. 2016, World Health Organization Regional Office for Europe: Copenhagen.

218. Last, J.M., *A dictionary of public health*. 2007, Oxford ; New York: Oxford University Press. viii, 407 p.

219. World Health Organization, *Hearts: technical pacakge for cardiovascular disease management in primary health care*. 2016, World Health Organization: Geneva.

220. *Modern health care delivery systems, care coordination and the role of hospitals. Compiled report of the workshop organized by the Beligum Federal Public Health Service and WHO Europe, held in Brussels, Belgium, 21-22 November 2011, and the internal WHO expert meeting on roadmap developmetn, held in Copenhagen, Denmark, 12 January 2012.* 2012, World Health Organization Regional Office for Europe: Copenhagen.

221. Longest, B. and G. Young *Coordination and communication*, in *Health care management : organization, design, and behavior. Delmar series in health services administration*, S.M. Shortell and A.D. Kaluzny, Editors. 2000, Delmar/Thomson Learning: Albany, N.Y. p. 210-243.

222. Smith, J.E., *Case management: a literature review.* Canadian journal of nursing administration, 1998. **11**(2): p. 93-109.

223. Medical Surgical Nursing Certification Board. *Care Coordination and Transition Mangement (CCTM) vs Case Management*. [cited 2018 July 8]; Available from: <https://www.msncb.org/sites/default/files/CCTM_vs_CM.pdf>.

224. *Clinical Practice Guidelines: Directions for a New Program*, in *Clinical Practice Guidelines: Directions for a New Program*, M.J. Field and K.N. Lohr, Editors. 1990: Washington (DC).

225. Organisation for Economic Co-operation and Development, *Health systems characteristics survey 2016. Glossary*. 2016, OECD Publishing: Paris.

226. World Health Organization. *Q&As on hypertension*. 2015 [cited 2018 August 3]; Available from: <http://www.who.int/features/qa/82/en/>.

227. *Primary Care Evaluation Tool*. 2010, World Health Organization Regional Office for Europe: Copenhagen.

228. Veillard, J.H., et al., *Health system stewardship of National Health Ministries in the WHO European region: concepts, functions and assessment framework.* Health Policy, 2011. **103**(2-3): p. 191-9.

229. World Health Organization. *WHO Model List of Essential Medicines*. 2018 [cited 2018 23 July]; Available from: [www.who.int/selection_medicines/list/en/](file:///Users/ericabarbazza/Downloads/www.who.int/selection_medicines/list/en).

230. Starfield, B., *Primary care: concept, evaluation, and policy*. 1992, London: Oxford University Press.

231. Allen, J., et al., *The European definition of general practice/family medicine*. 2011, WONCA Europe - The World Organization of National Colleges, Academies and Academic Associations of General Practitioners/Family Physicians.

232. Wellcentive, P. *What is population health management?* . 2018; Available from: <https://www.wellcentive.com/what-is-population-health-management/>.

233. Department of Child Adolescent Health and Development, *Orientation programme on adolescent health for health-care providers*. World Health Organization: Geneva.

234. Kutzin, J., *Health financing policy: a guide for decision-makers*. 2008, World Health Organization Regional Office for Europe: Copenhagen.

235. Agency for Healthcare Research and Quality, *Practice facilitation handbook: creating quality improvement teams and QI plans*. 2013, Agency for Healthcare Research and Quality: Rockville, MD.

236. *Integrated health services delivery networks: concepts, policy options and a road map for implementation in the Americas. Renewing primary health care in the Americas Series No.4*. 2011, Pan American health Organization: Washington, DC.

237. *Everybody business: strengthening health systems to improve health outcomes: WHO's framework for action.* 2007, Geneva: World Health Organization.

238. Atun, R., *What are the advantages and disadvantages of restructuring a health care system to be more focused on primary care services?* 2004, World Health Organization Regional Office for Europe. Health Evidence Network: Copenhagen.

239. *The world health report 2000 - Health systems: improving performance*. 2000, World Health Organization: Geneva.

240. United Nations Educational Scientific and Cultural Organization (UNESCO), *International Standard Classification of Education (ISCED) 2011*. 2012, UNESCO Institute for Statistics: Montreal, Quebec Canada.

241. Struckmann, V., et al., *How to strengthen financing mechanisms to promote care for people with multimorbidity in Europe?* 2016, ICARE4EU project.

242. World Health Organization. *Health financing for universal coverage; provider payment mechanisms*. 2015 [cited 2018 23 June]; Available from: <http://www.who.int/health_financing/topics/purchasing/payment-mechanisms/en/>.

243. *Quality of care : a process for making strategic choices in health systems*. 2006, World Health Organization: Geneva. p. viii, 38 pages.

244. World Health Organization. *Management for health services delivery; managemetn of health facilities: referral systems*. 2008 [cited 2018 24 July]; Available from: [www.who.int/management/facility/referral/en/](file:///Users/ericabarbazza/Downloads/www.who.int/management/facility/referral/en).

245. OECD, *Digital economy outlook 2017*. 2017, OECD Paris

246. Barnes, R., *Health Impact Assessment. Glossary of terms used*. World Health Organization: Copenhagen.
